# Supplementary figures and images for: Spatiotemporal distribution of caudal-type homeobox proteins during development of the hindgut and anorectum in human embryos (part 1 of 2)
Source: PeerJ. 2016 Mar 24;4:e1771. doi: 10.7717/peerj.1771 (PMC4811170; doi:10.7717/peerj.1771)

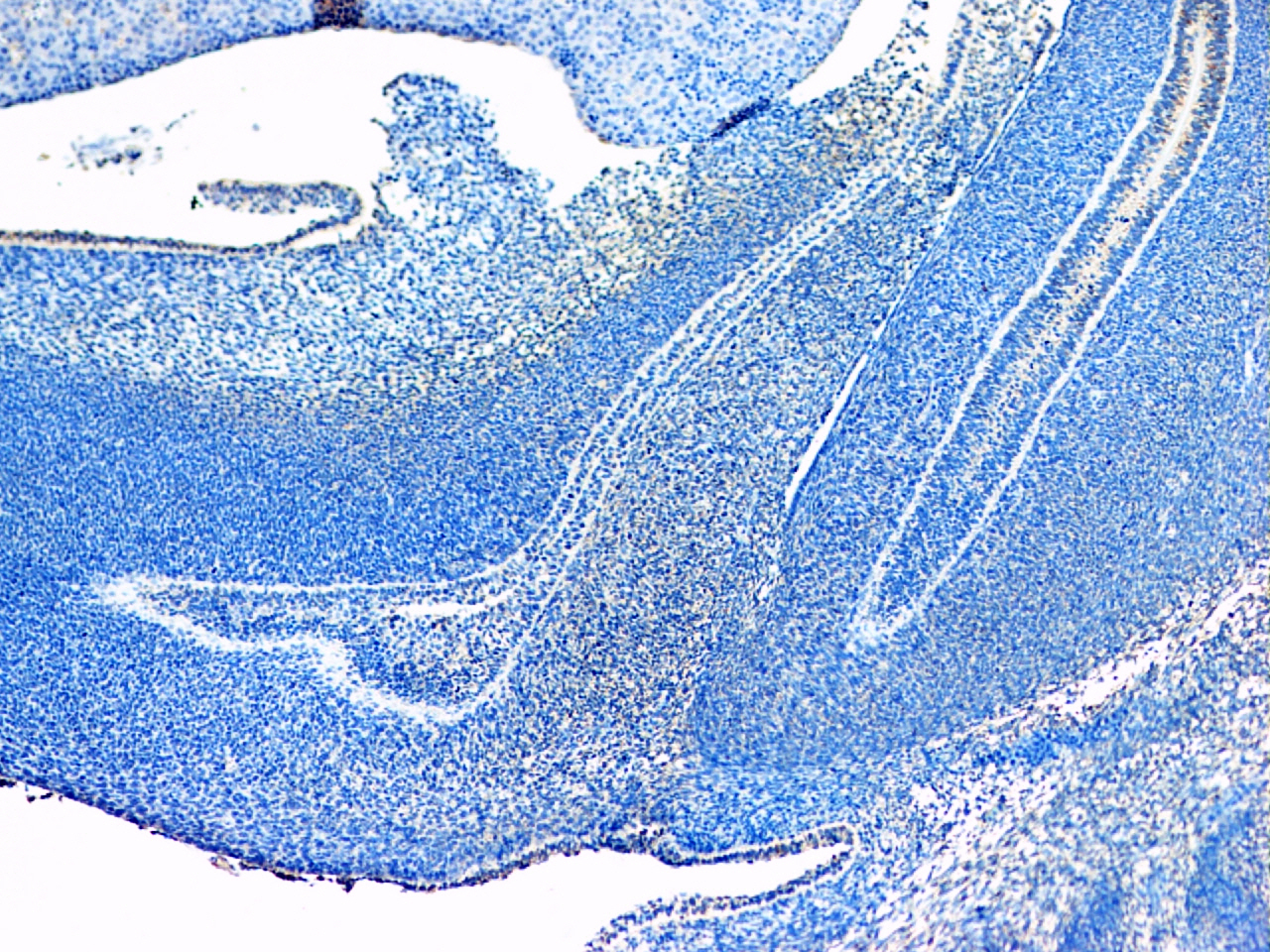

Supplement: Supplemental Information 1 [file peerj-04-1771-s001.zip › 1/c1-1-13 100╡≈╒√.jpg]

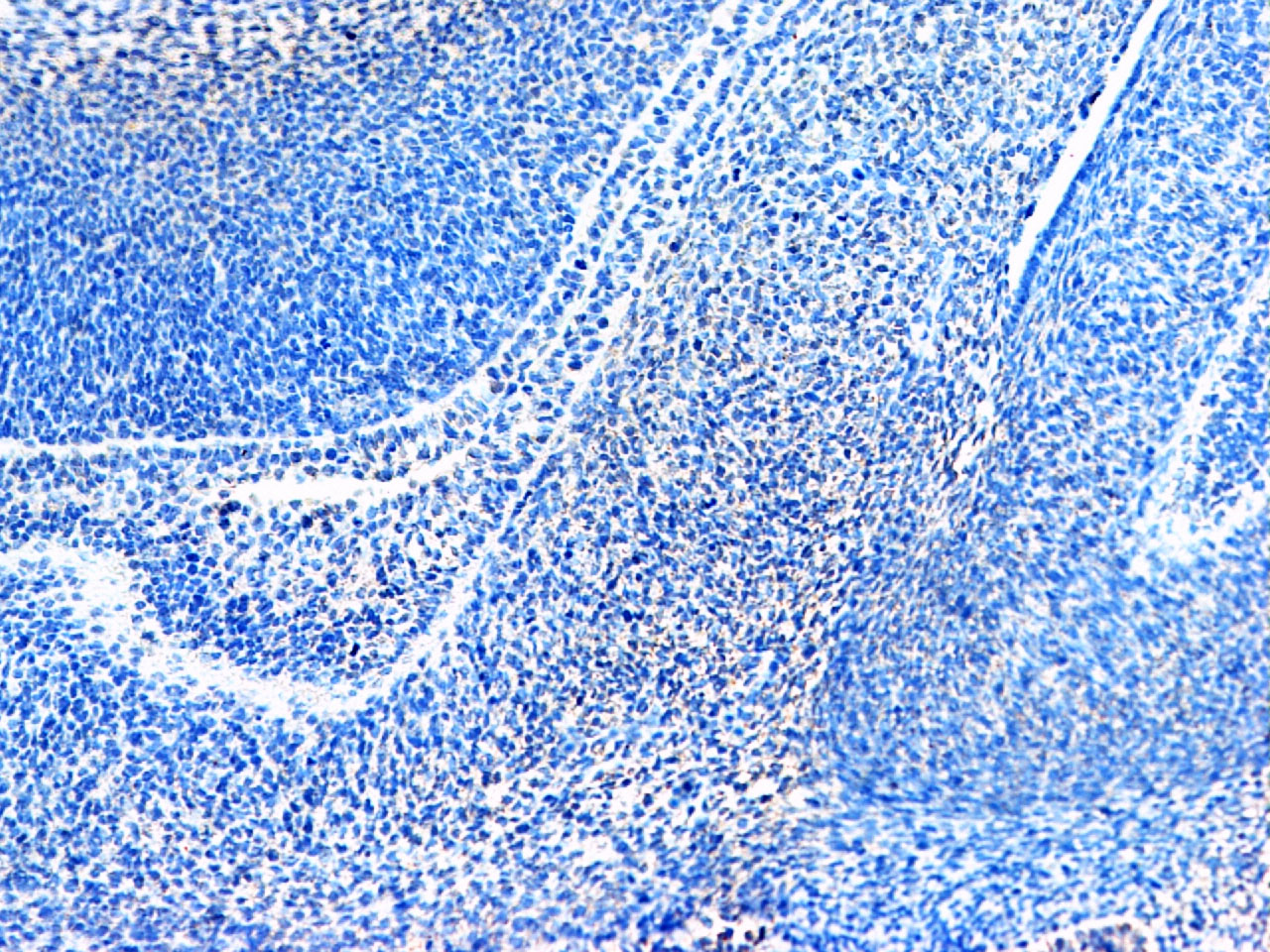

Supplement: Supplemental Information 1 [file peerj-04-1771-s001.zip › 1/c1-1-13 200╡≈╒√.jpg]

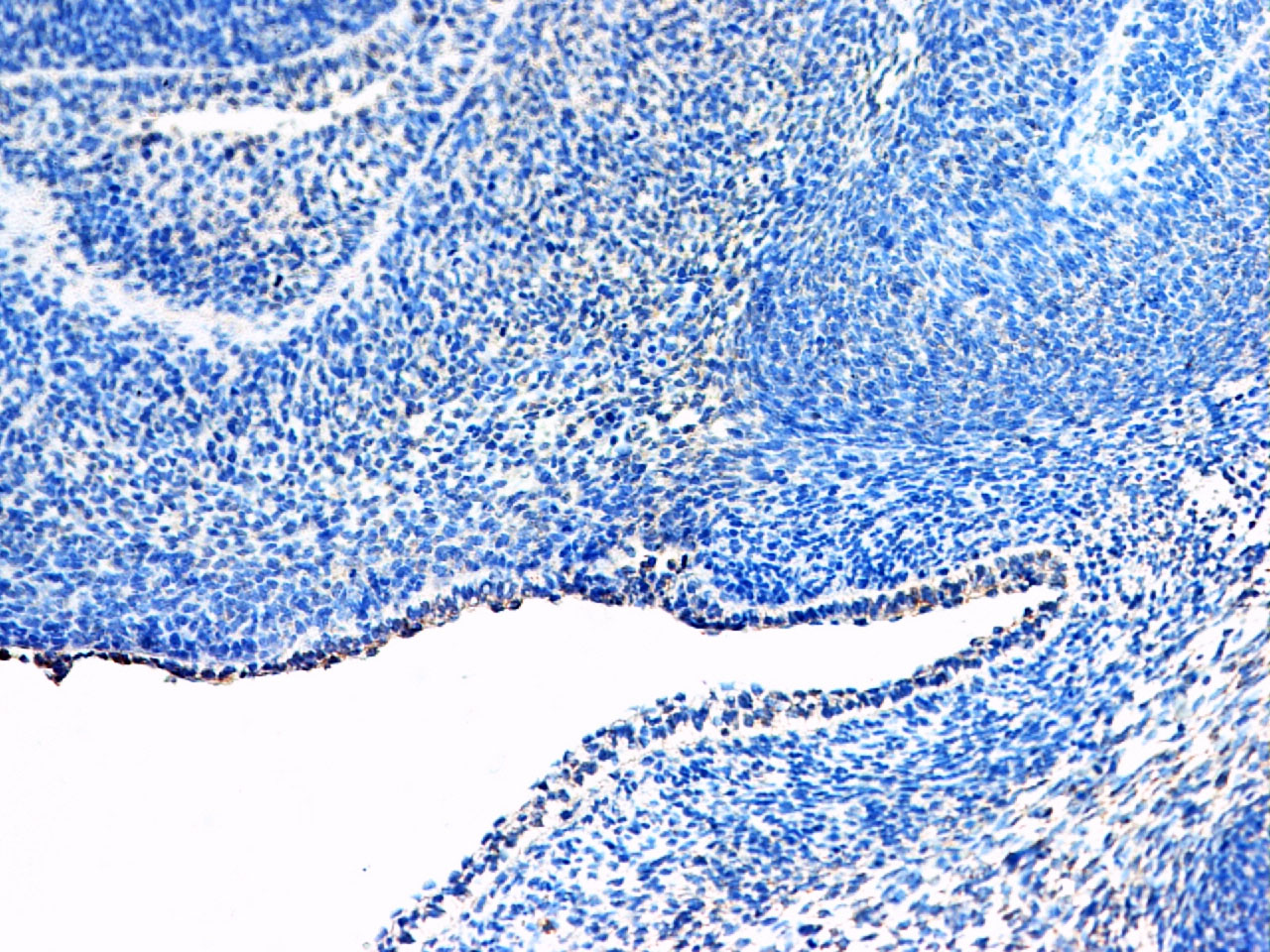

Supplement: Supplemental Information 1 [file peerj-04-1771-s001.zip › 1/c1-1-13 400í»╡≈╒√.jpg]

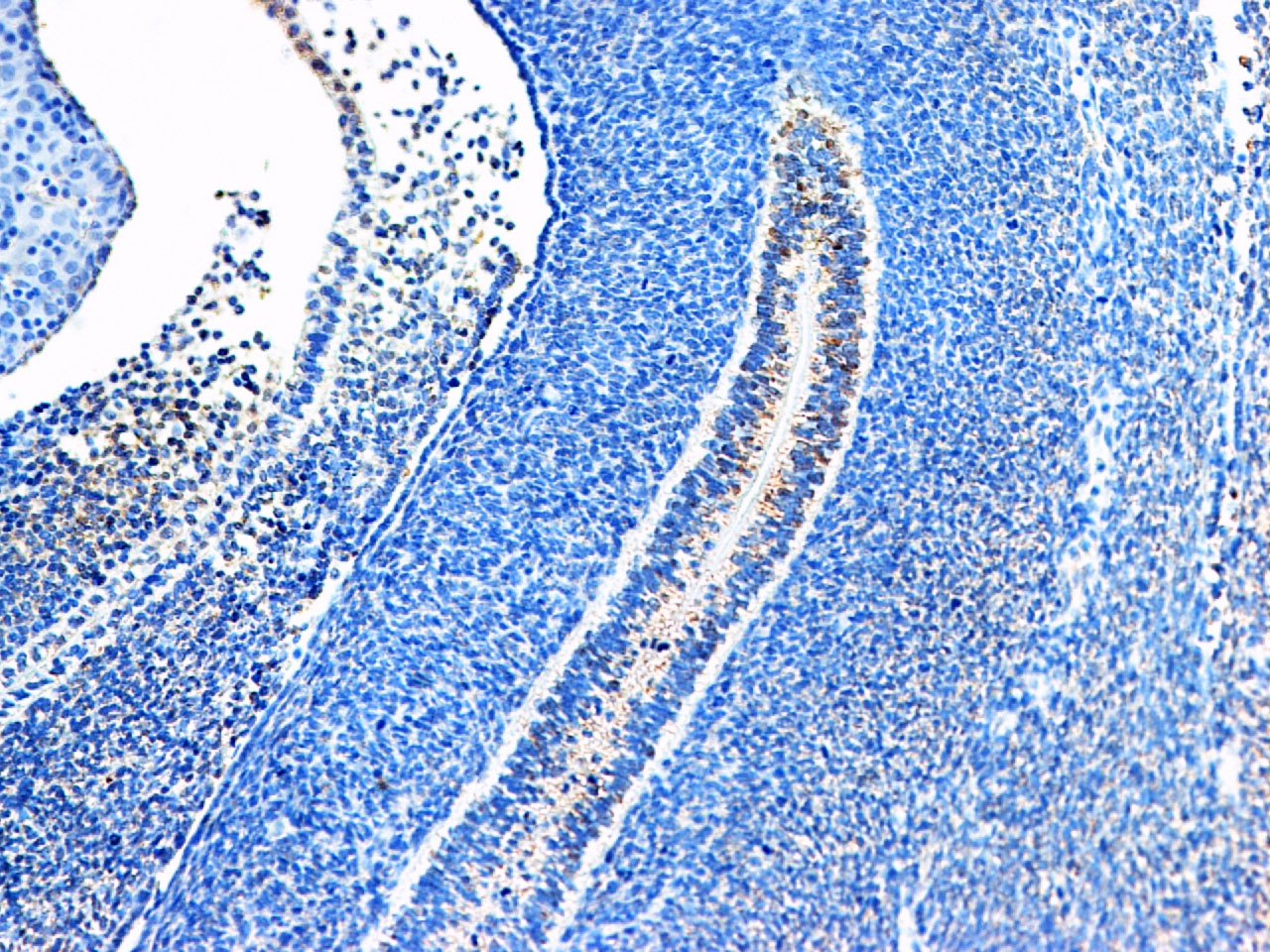

Supplement: Supplemental Information 1 [file peerj-04-1771-s001.zip › 1/c1-1-13 400╡≈╒√.jpg]

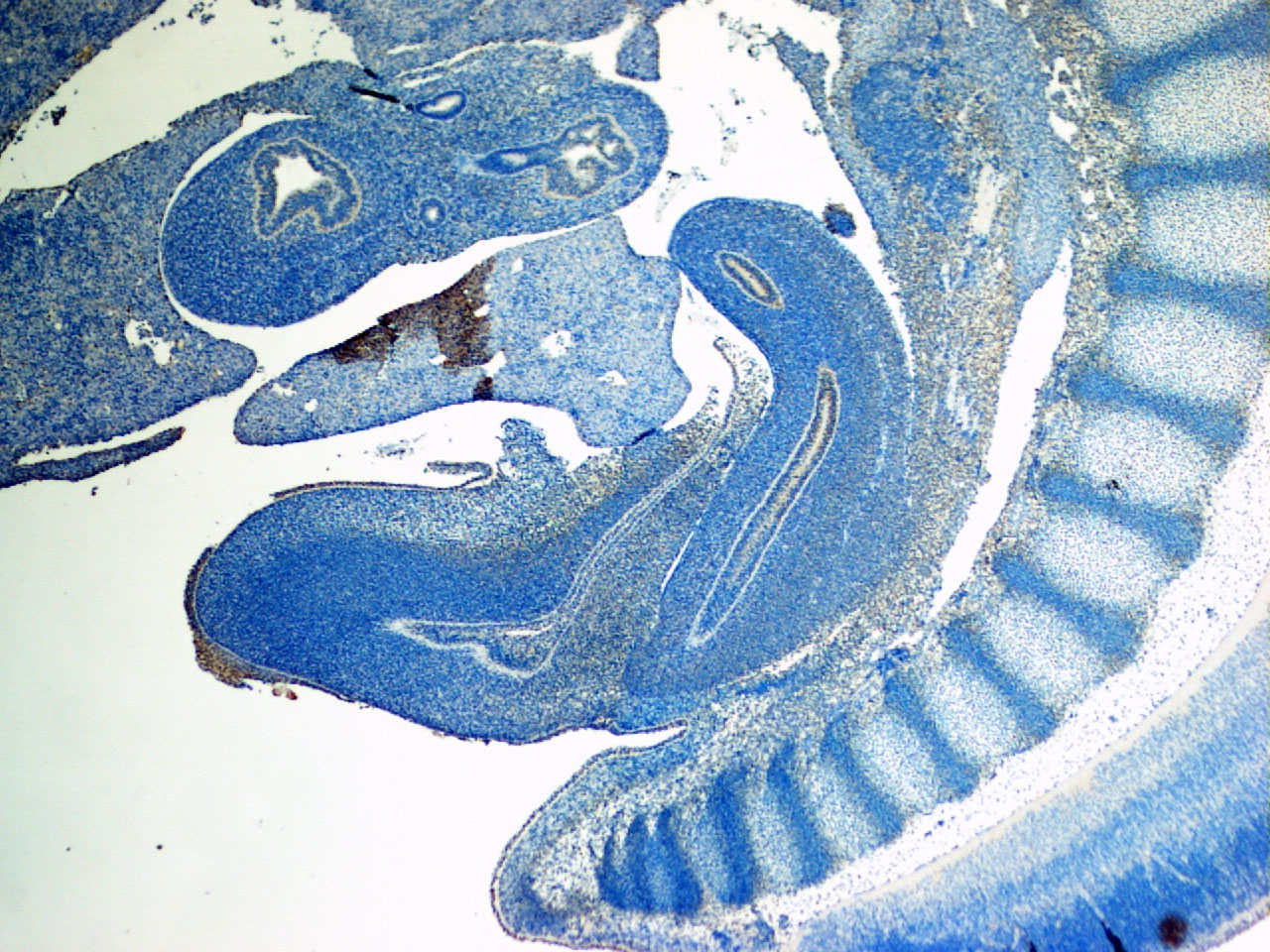

Supplement: Supplemental Information 1 [file peerj-04-1771-s001.zip › 1/c1-1-13 40╡≈╒√.jpg]

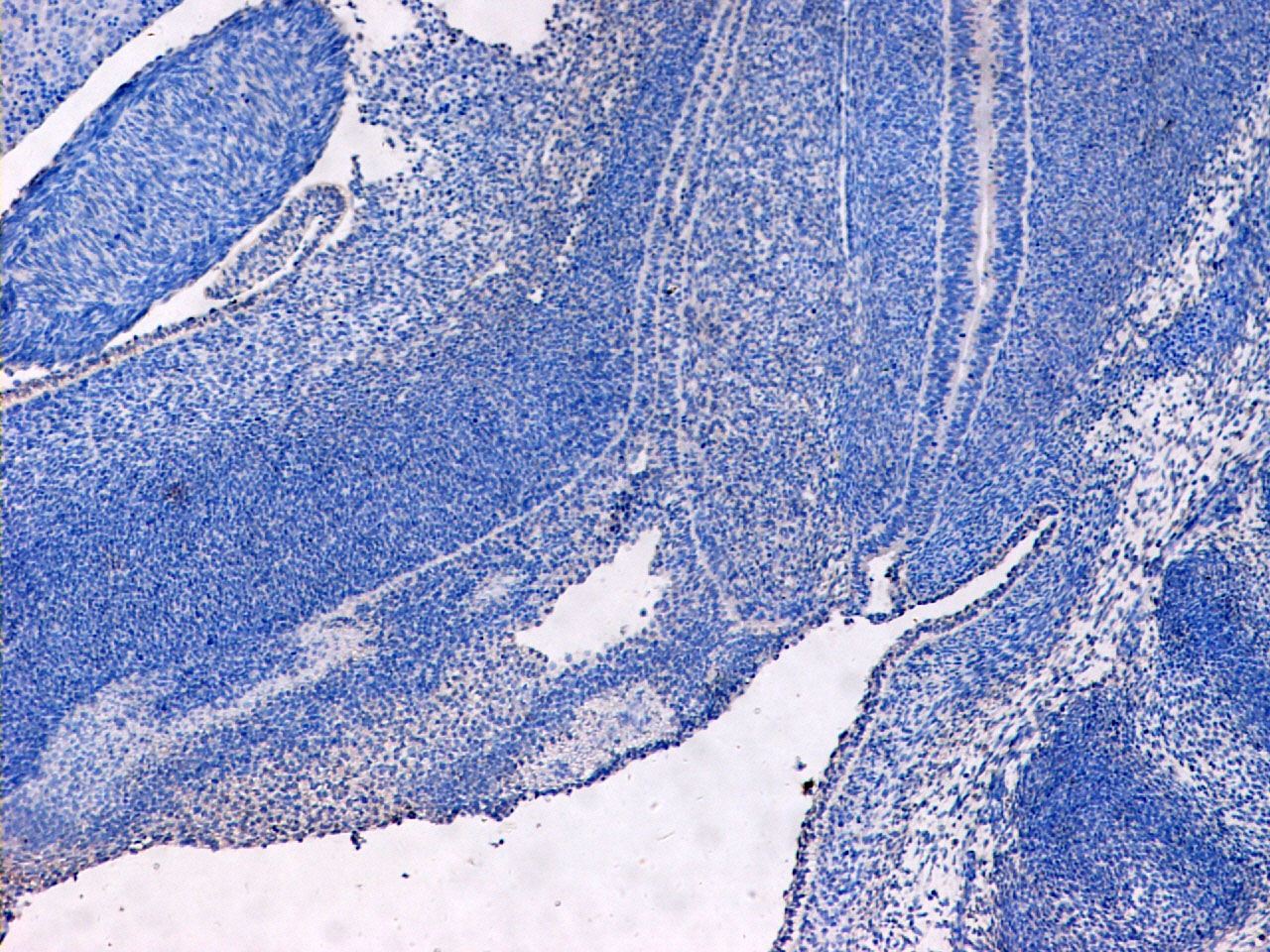

Supplement: Supplemental Information 1 [file peerj-04-1771-s001.zip › 1/C1-1-9 100▒╢'╡≈╒√.jpg]

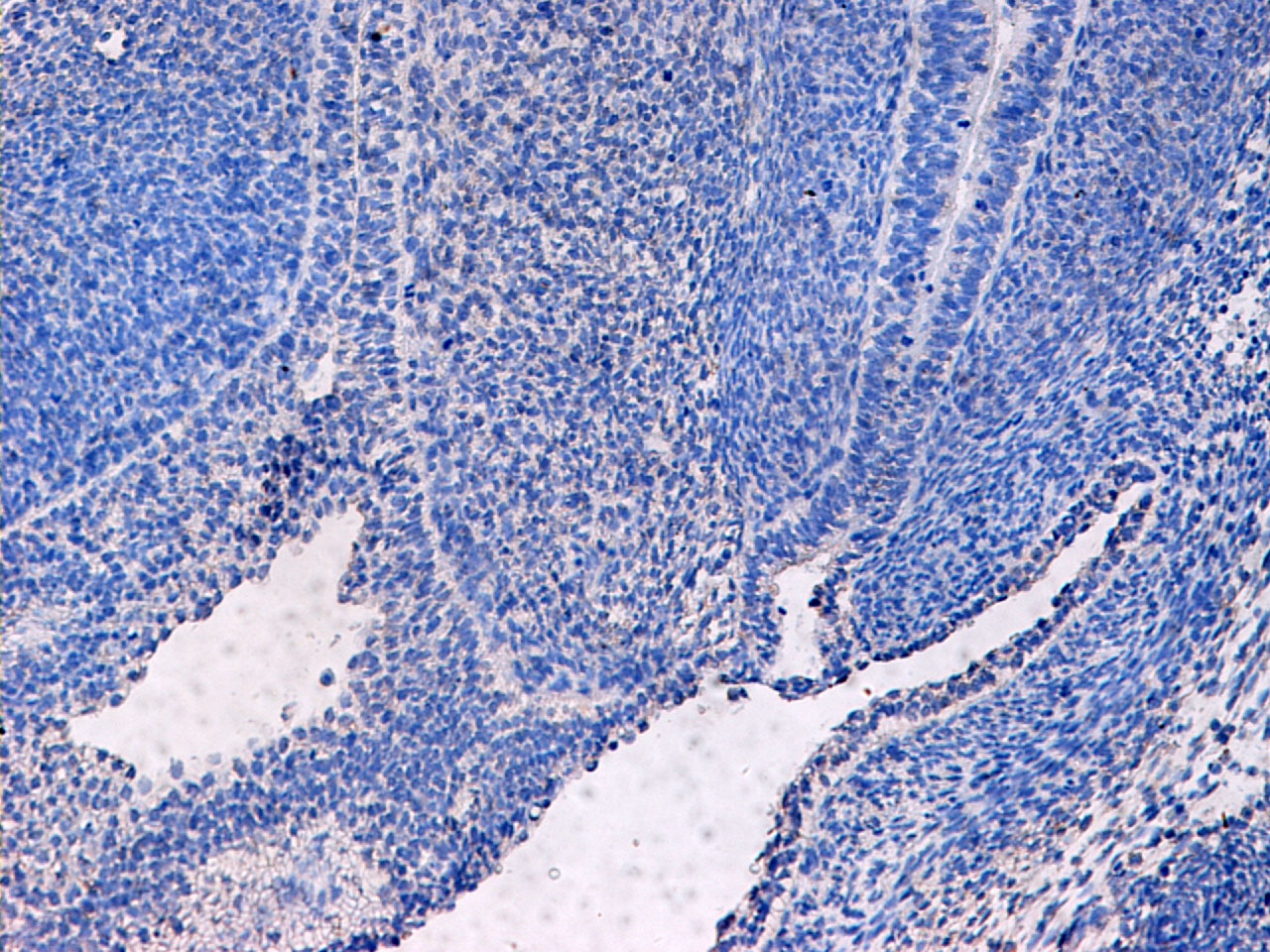

Supplement: Supplemental Information 1 [file peerj-04-1771-s001.zip › 1/C1-1-9 200▒╢╡≈╒√.jpg]

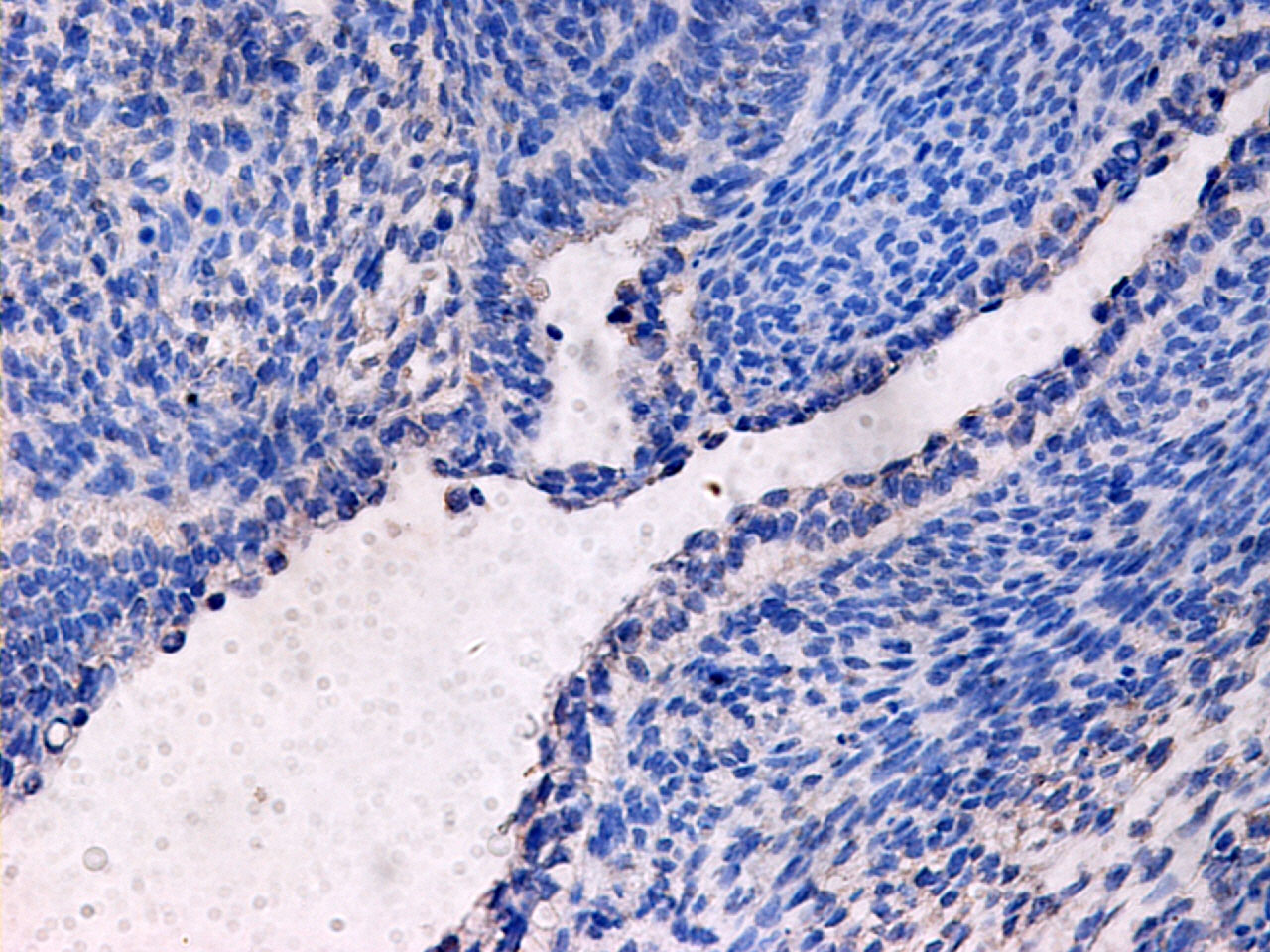

Supplement: Supplemental Information 1 [file peerj-04-1771-s001.zip › 1/C1-1-9 400▒╢╡≈╒√.jpg]

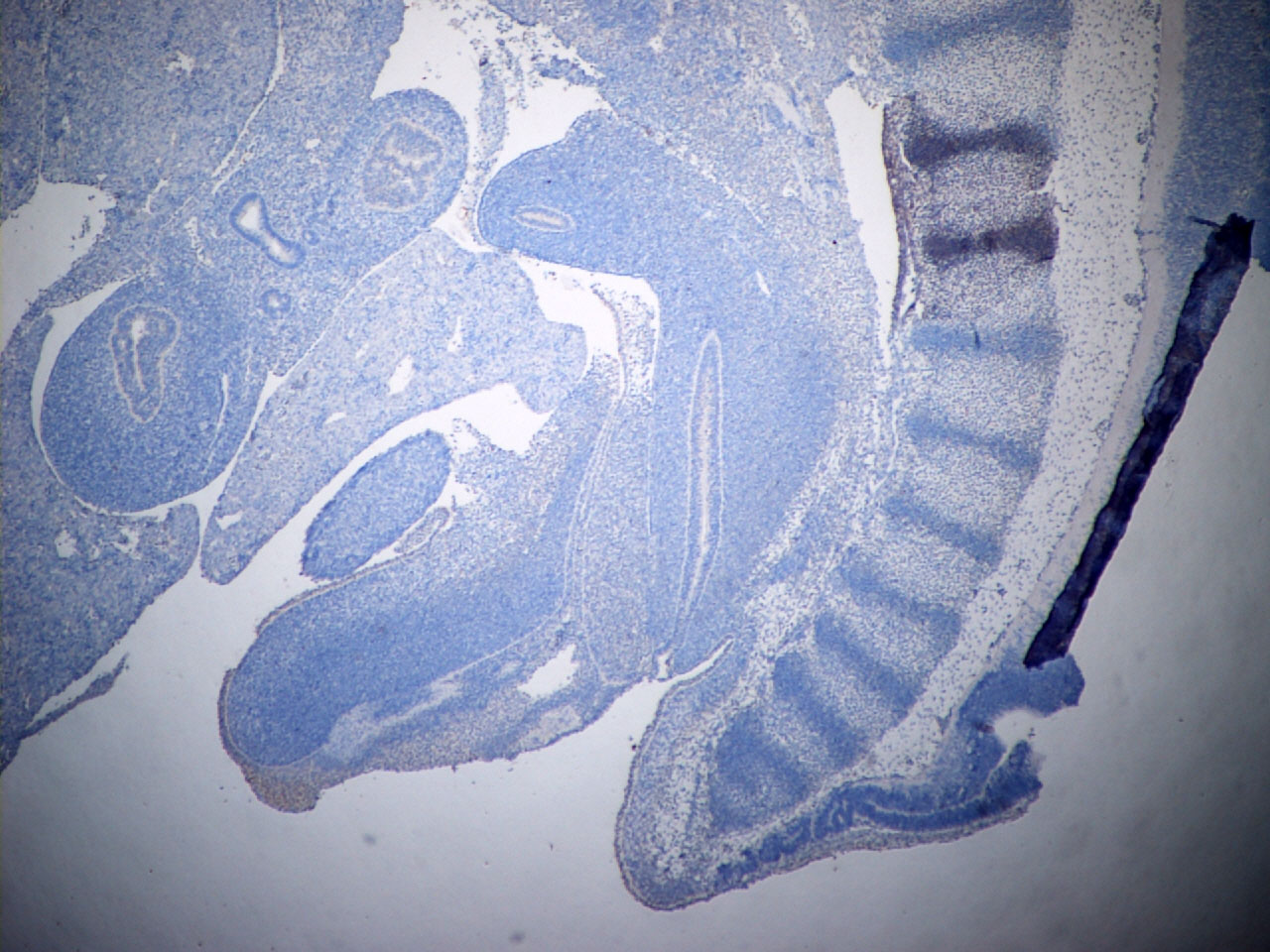

Supplement: Supplemental Information 1 [file peerj-04-1771-s001.zip › 1/C1-1-9 40▒╢╡≈╒√.jpg]

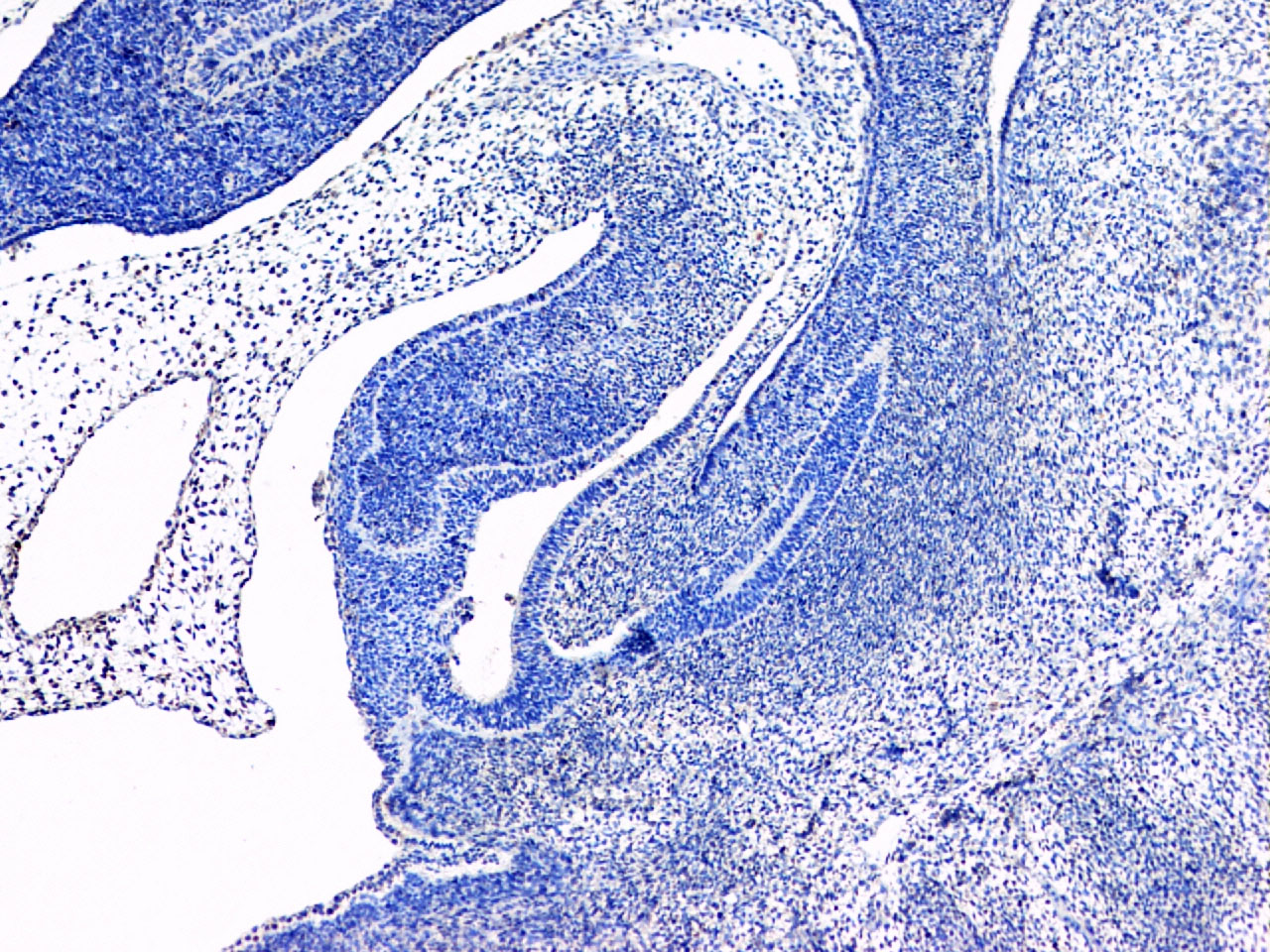

Supplement: Supplemental Information 1 [file peerj-04-1771-s001.zip › 1/c1-11-11 100╡≈╒√.jpg]

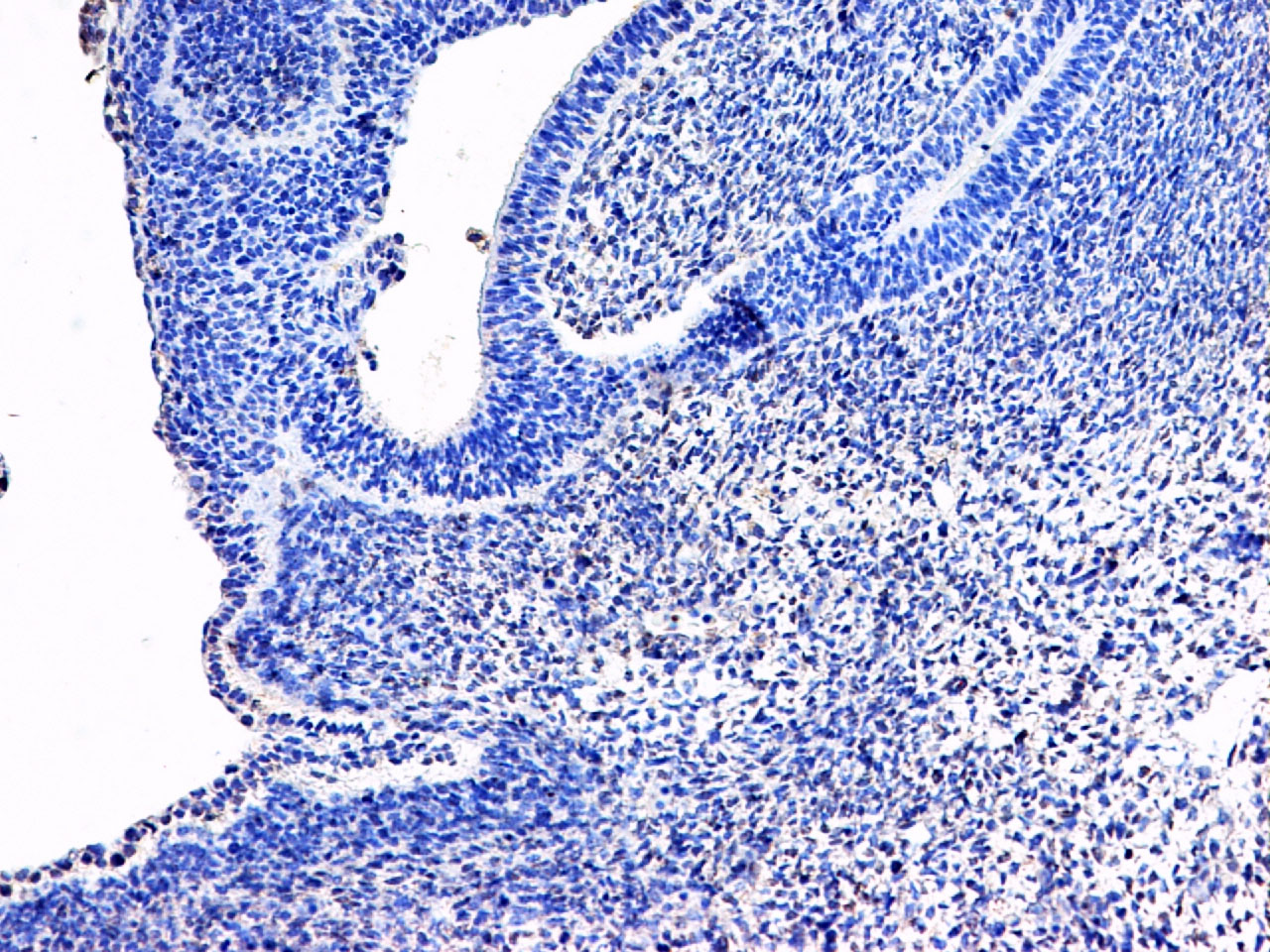

Supplement: Supplemental Information 1 [file peerj-04-1771-s001.zip › 1/c1-11-11 200'╡≈╒√.jpg]

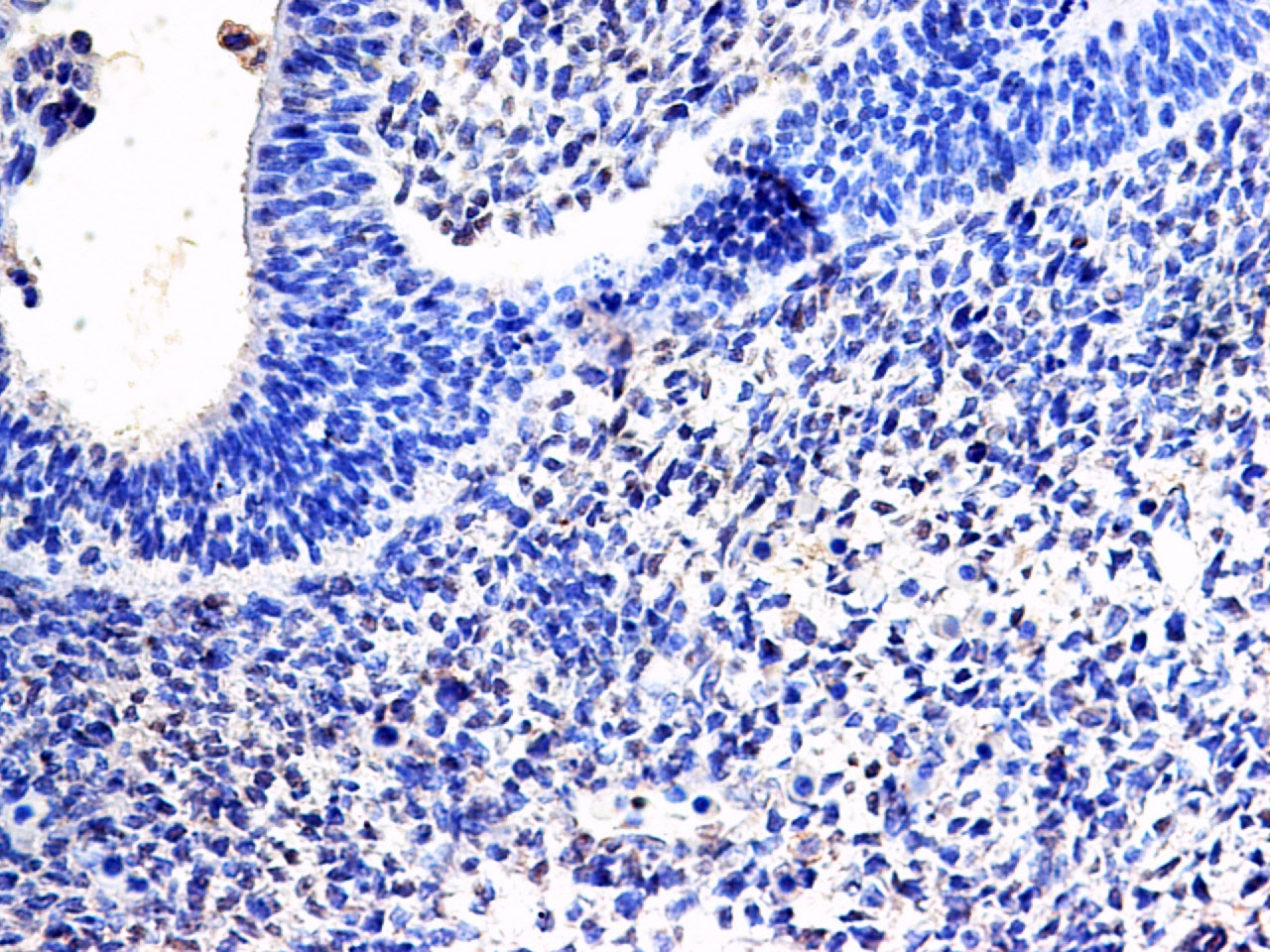

Supplement: Supplemental Information 1 [file peerj-04-1771-s001.zip › 1/c1-11-11 400╡≈╒√.jpg]

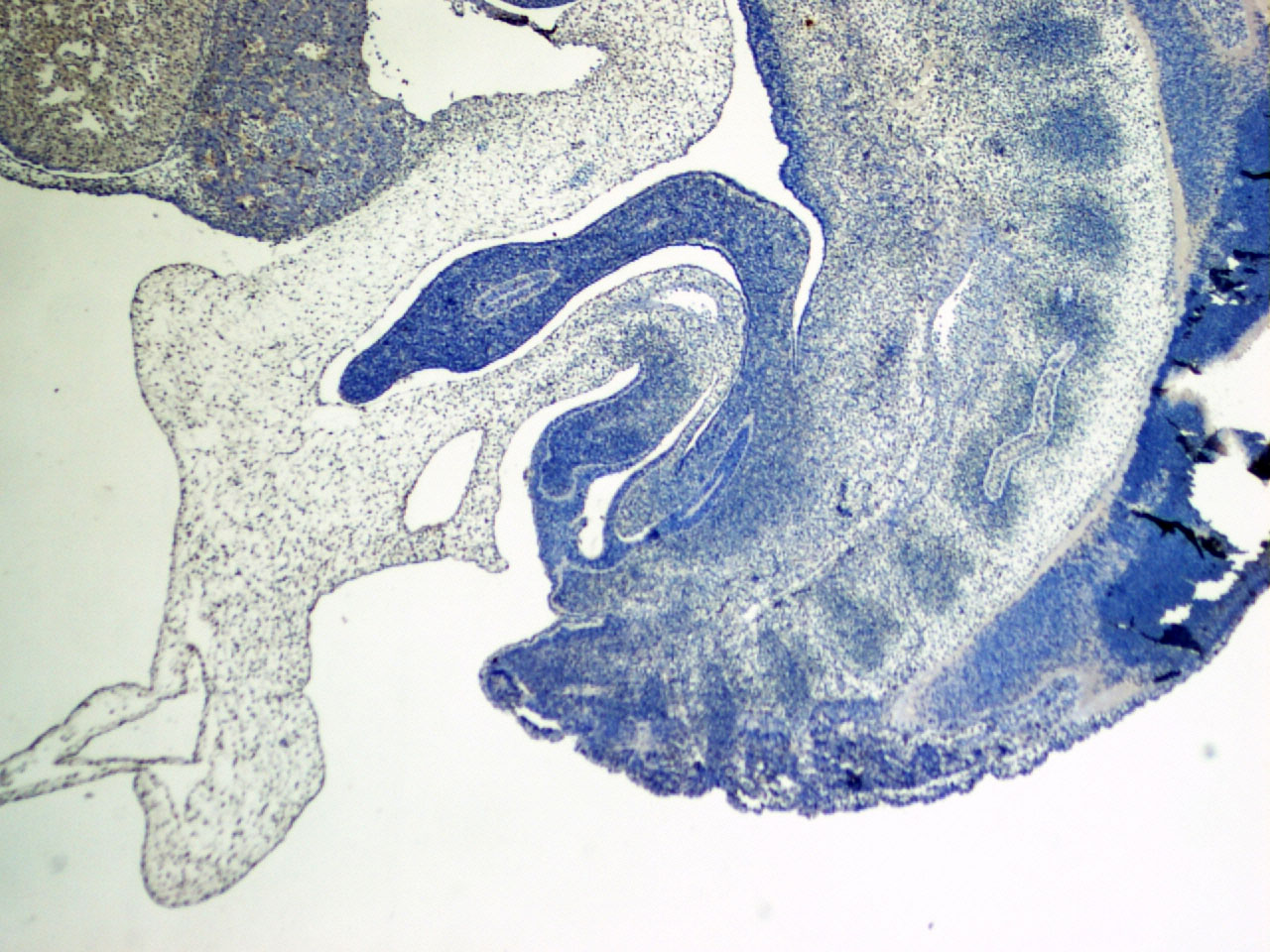

Supplement: Supplemental Information 1 [file peerj-04-1771-s001.zip › 1/c1-11-11 40╡≈╒√.jpg]

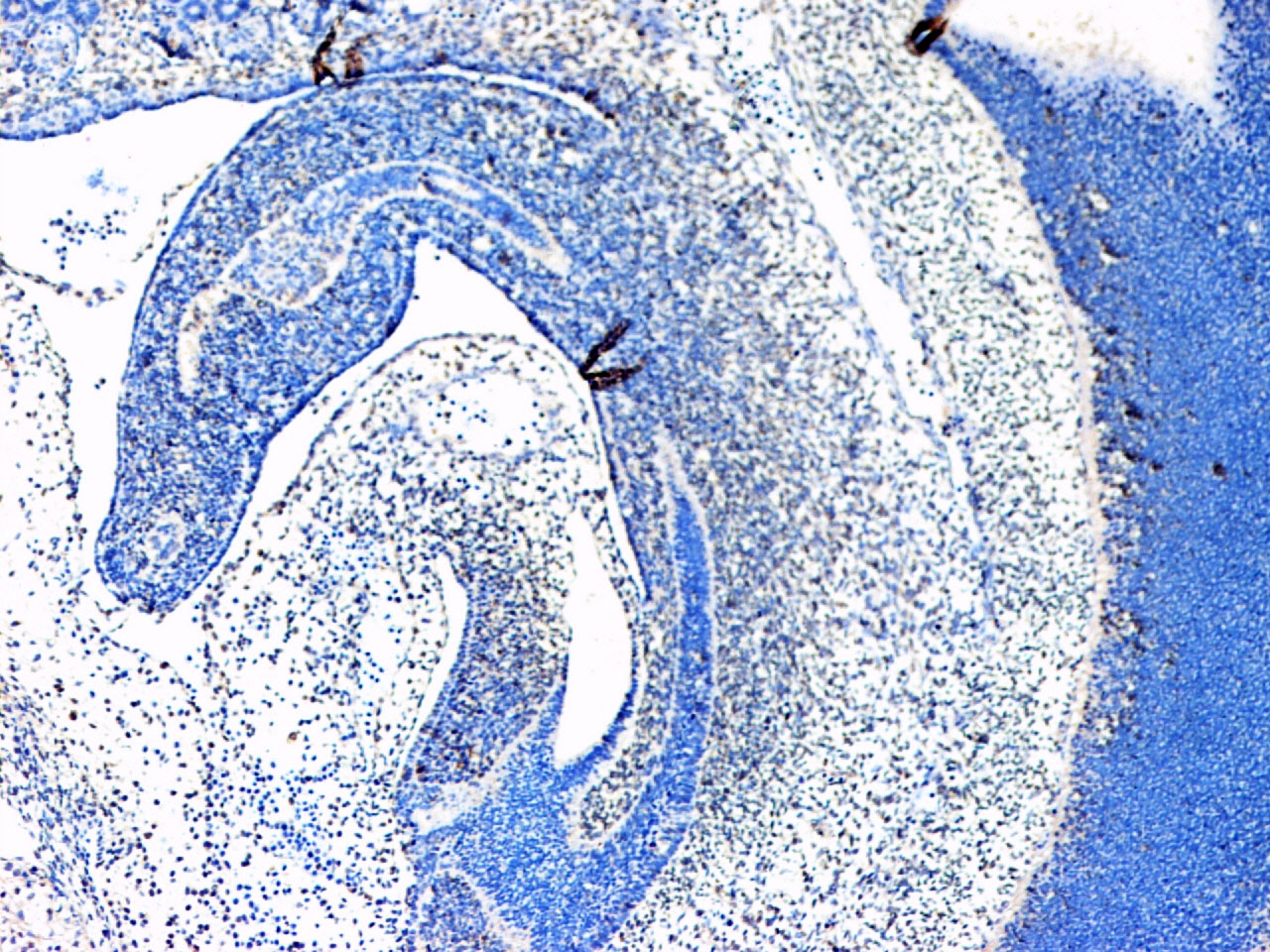

Supplement: Supplemental Information 1 [file peerj-04-1771-s001.zip › 1/c1-13-5 100╡≈╒√.jpg]

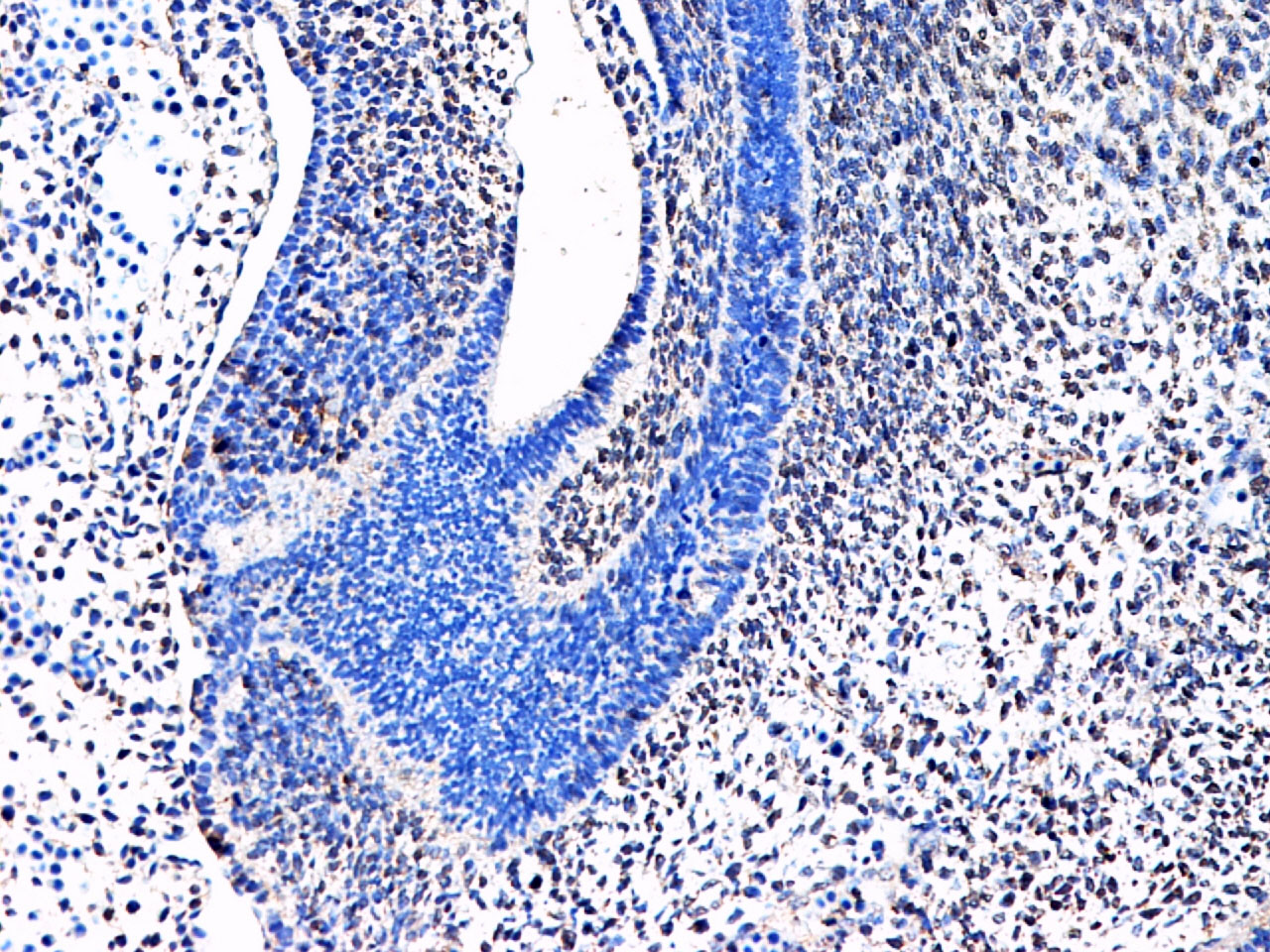

Supplement: Supplemental Information 1 [file peerj-04-1771-s001.zip › 1/c1-13-5 200╡≈╒√.jpg]

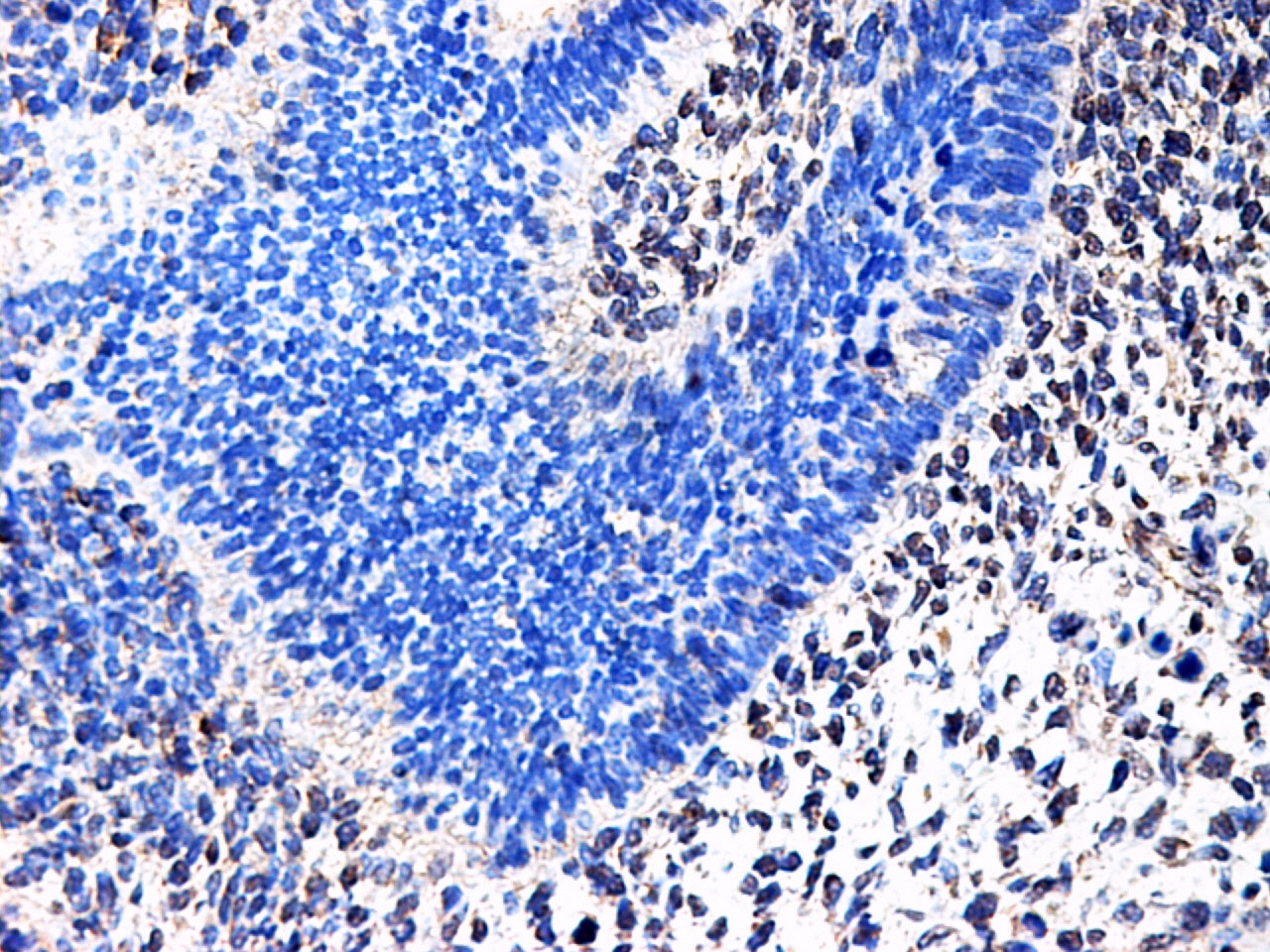

Supplement: Supplemental Information 1 [file peerj-04-1771-s001.zip › 1/c1-13-5 400╡≈╒√.jpg]

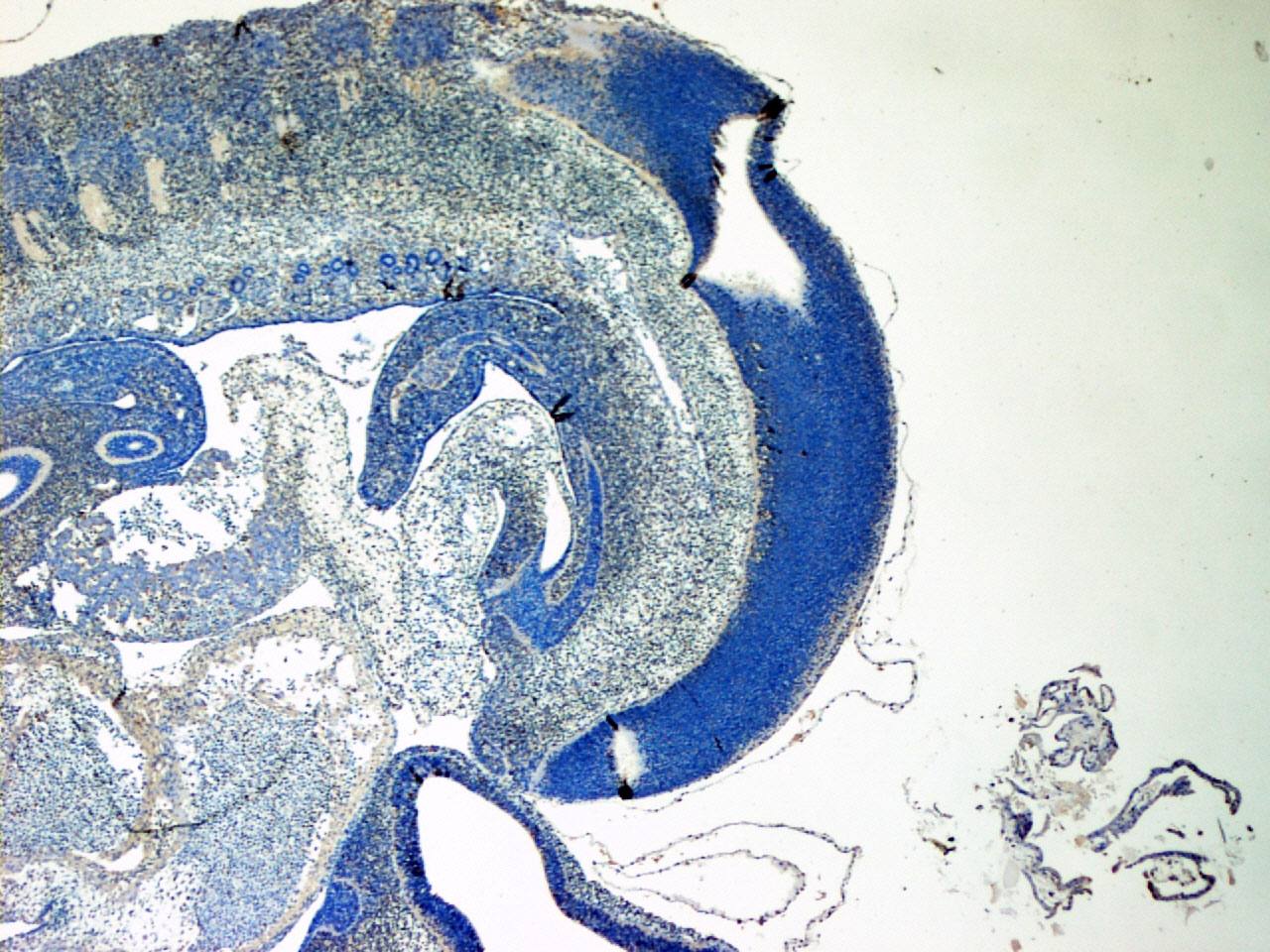

Supplement: Supplemental Information 1 [file peerj-04-1771-s001.zip › 1/c1-13-5 40╡≈╒√.jpg]

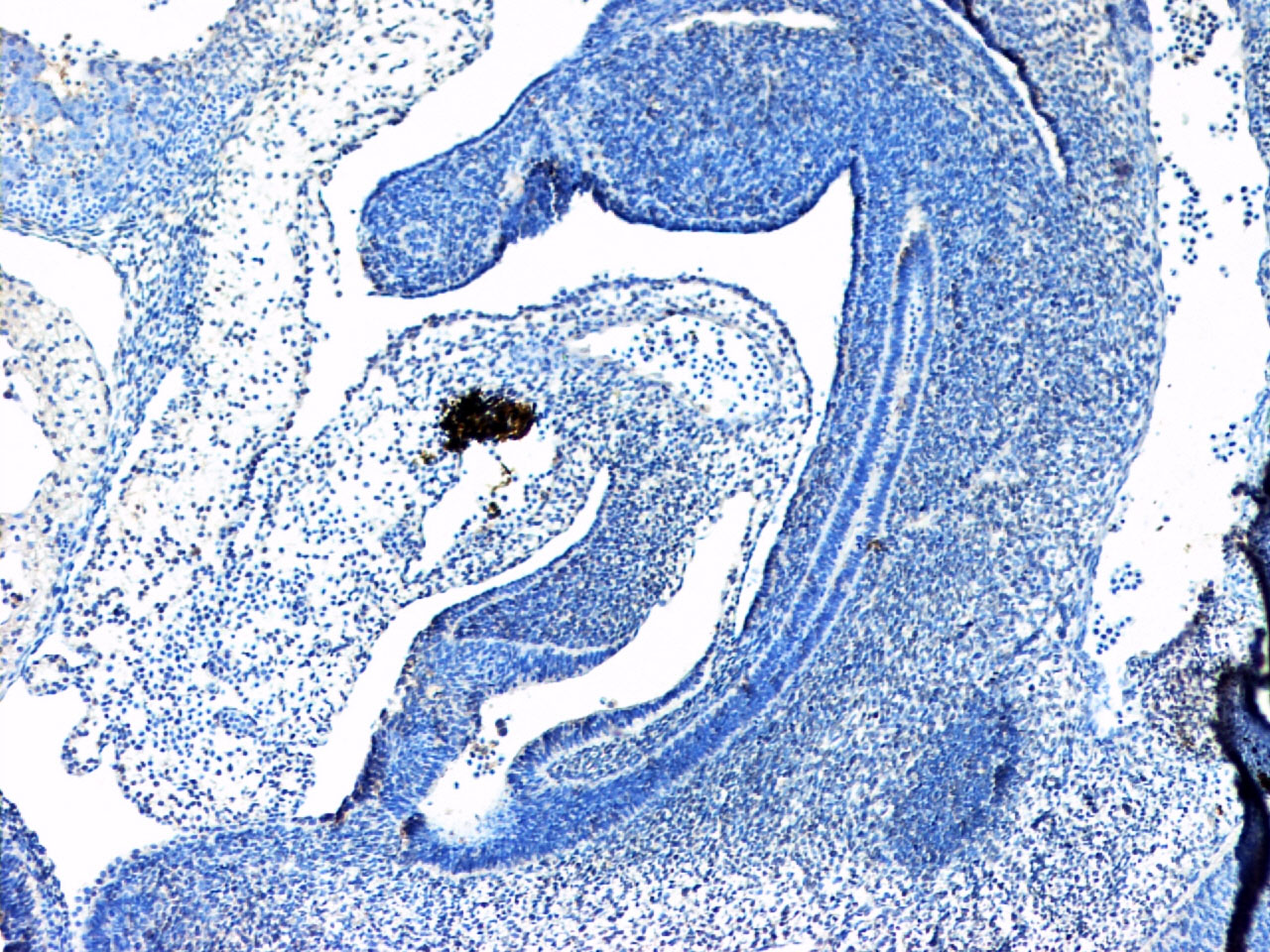

Supplement: Supplemental Information 1 [file peerj-04-1771-s001.zip › 1/c1-13-6 100╡≈╒√.jpg]

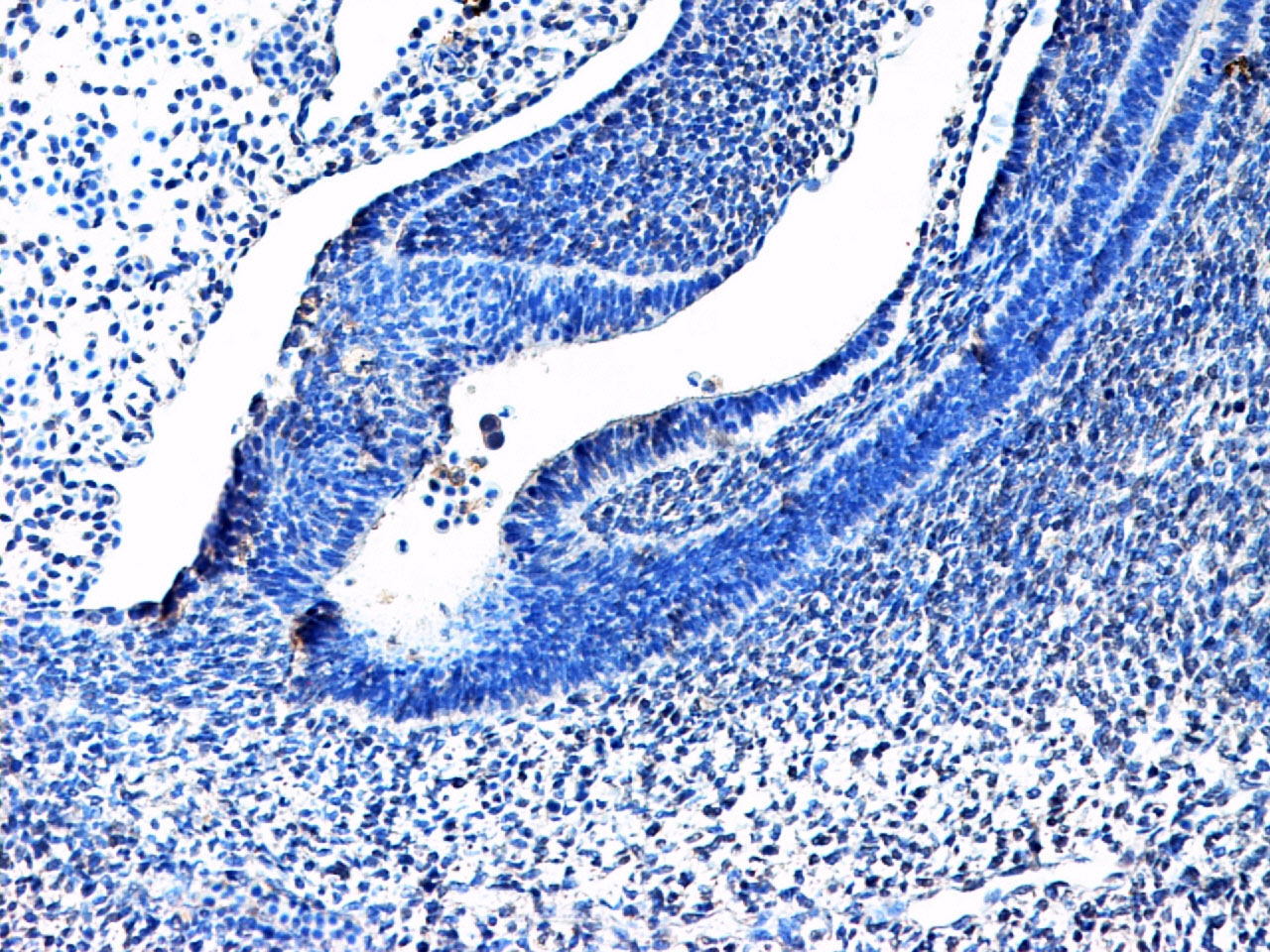

Supplement: Supplemental Information 1 [file peerj-04-1771-s001.zip › 1/c1-13-6 200╡≈╒√.jpg]

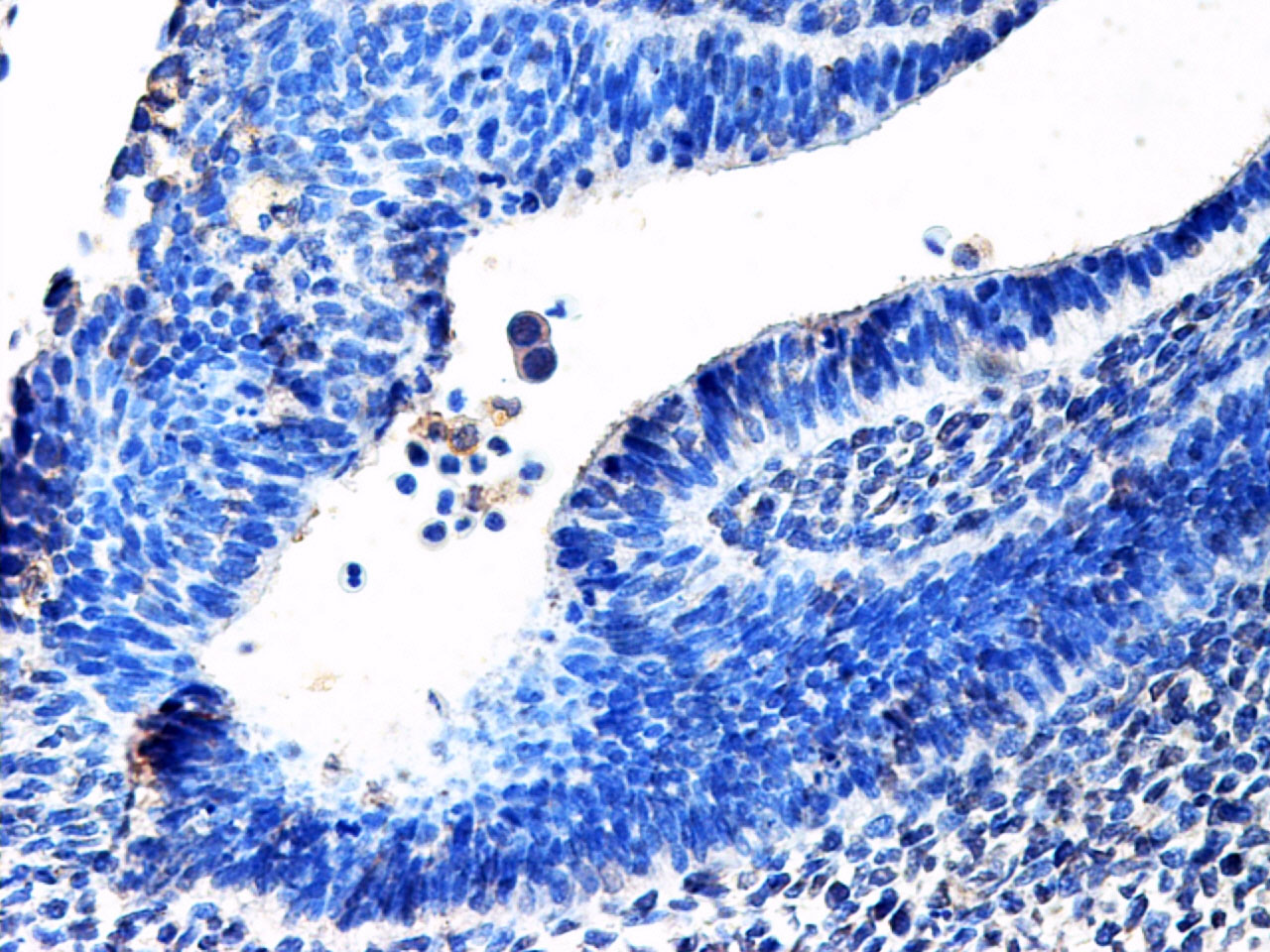

Supplement: Supplemental Information 1 [file peerj-04-1771-s001.zip › 1/c1-13-6 400'╡≈╒√.jpg]

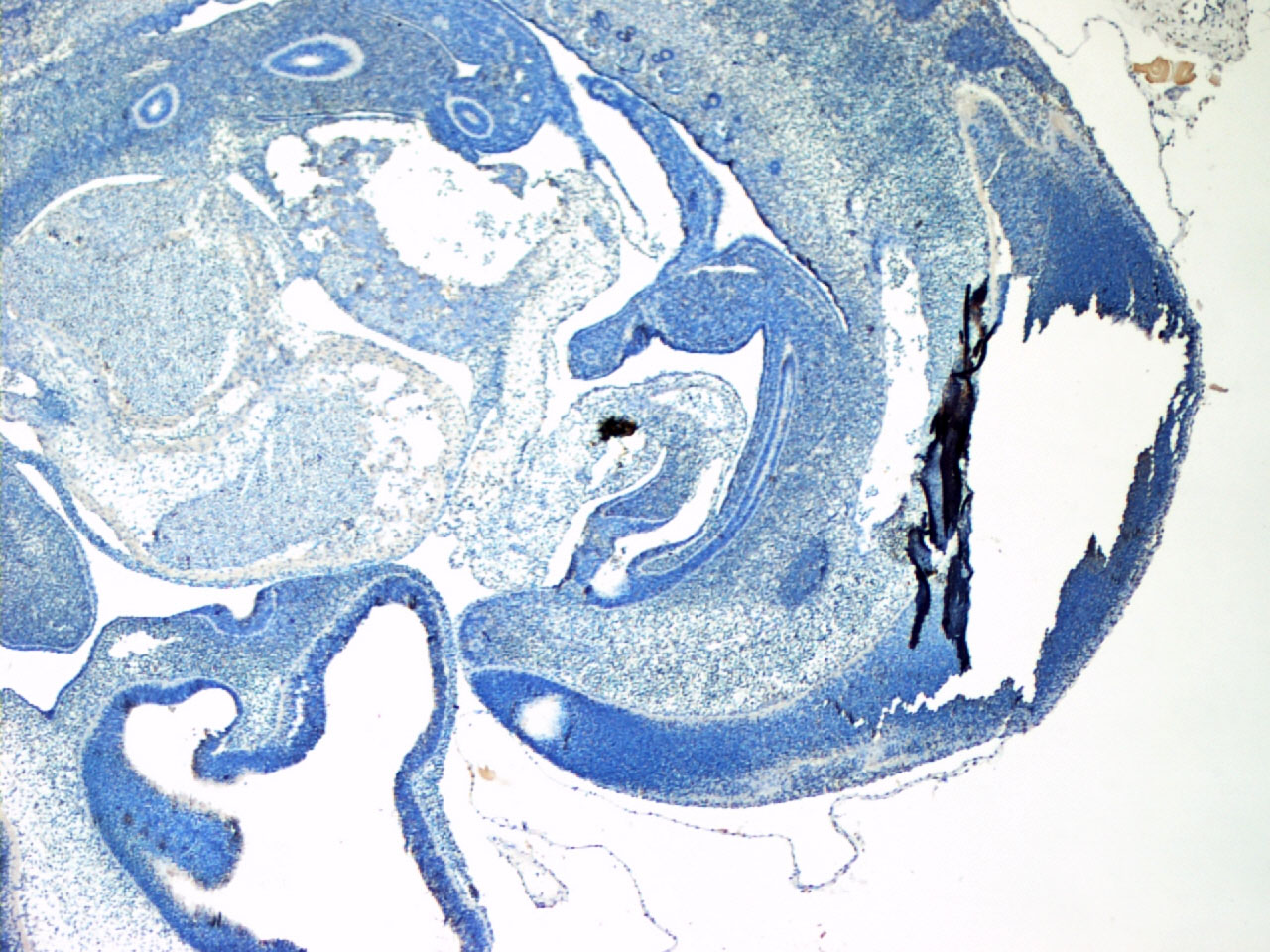

Supplement: Supplemental Information 1 [file peerj-04-1771-s001.zip › 1/c1-13-6 40╡≈╒√.jpg]

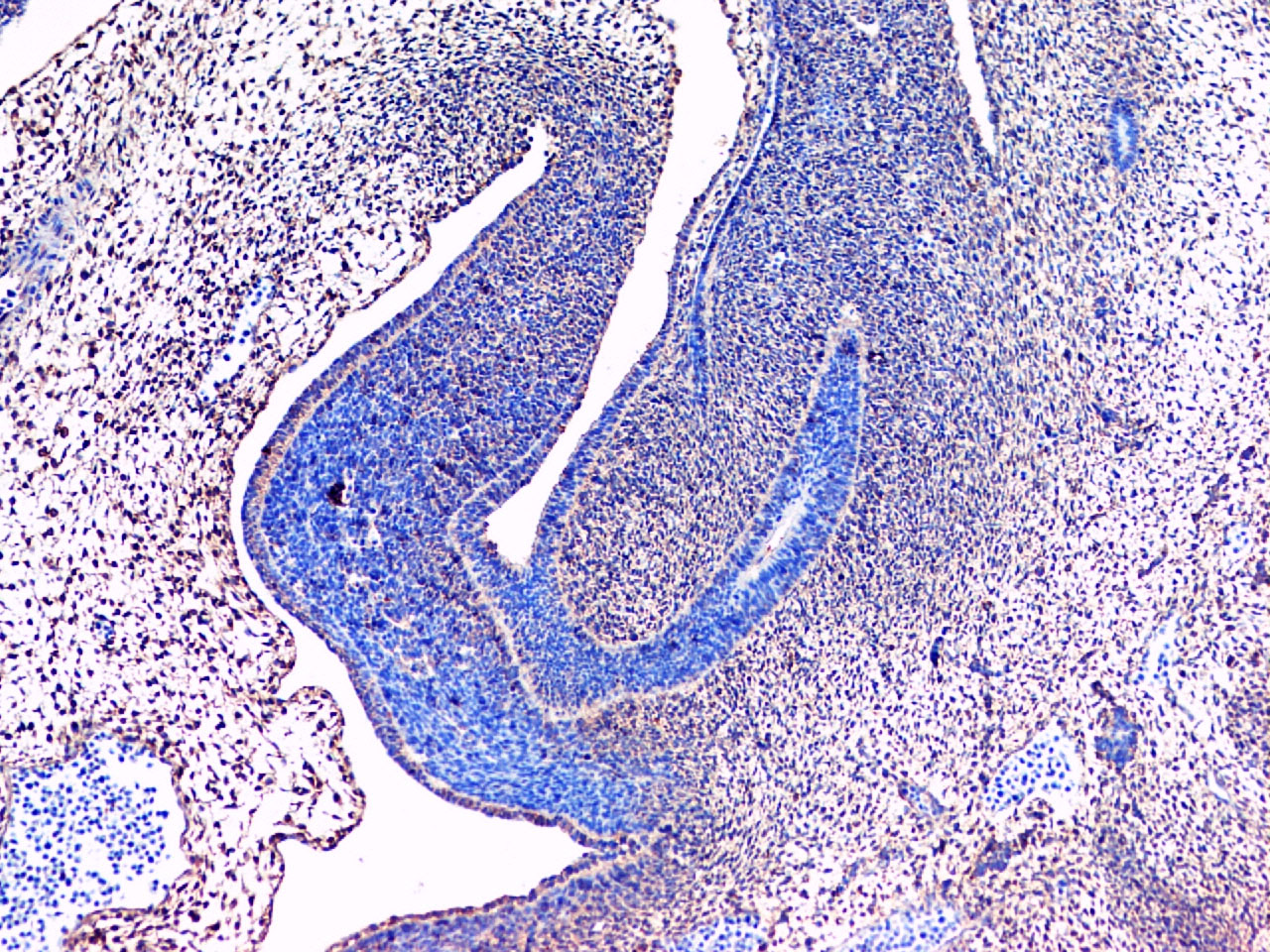

Supplement: Supplemental Information 1 [file peerj-04-1771-s001.zip › 1/c1-25-10 100╡≈╒√.jpg]

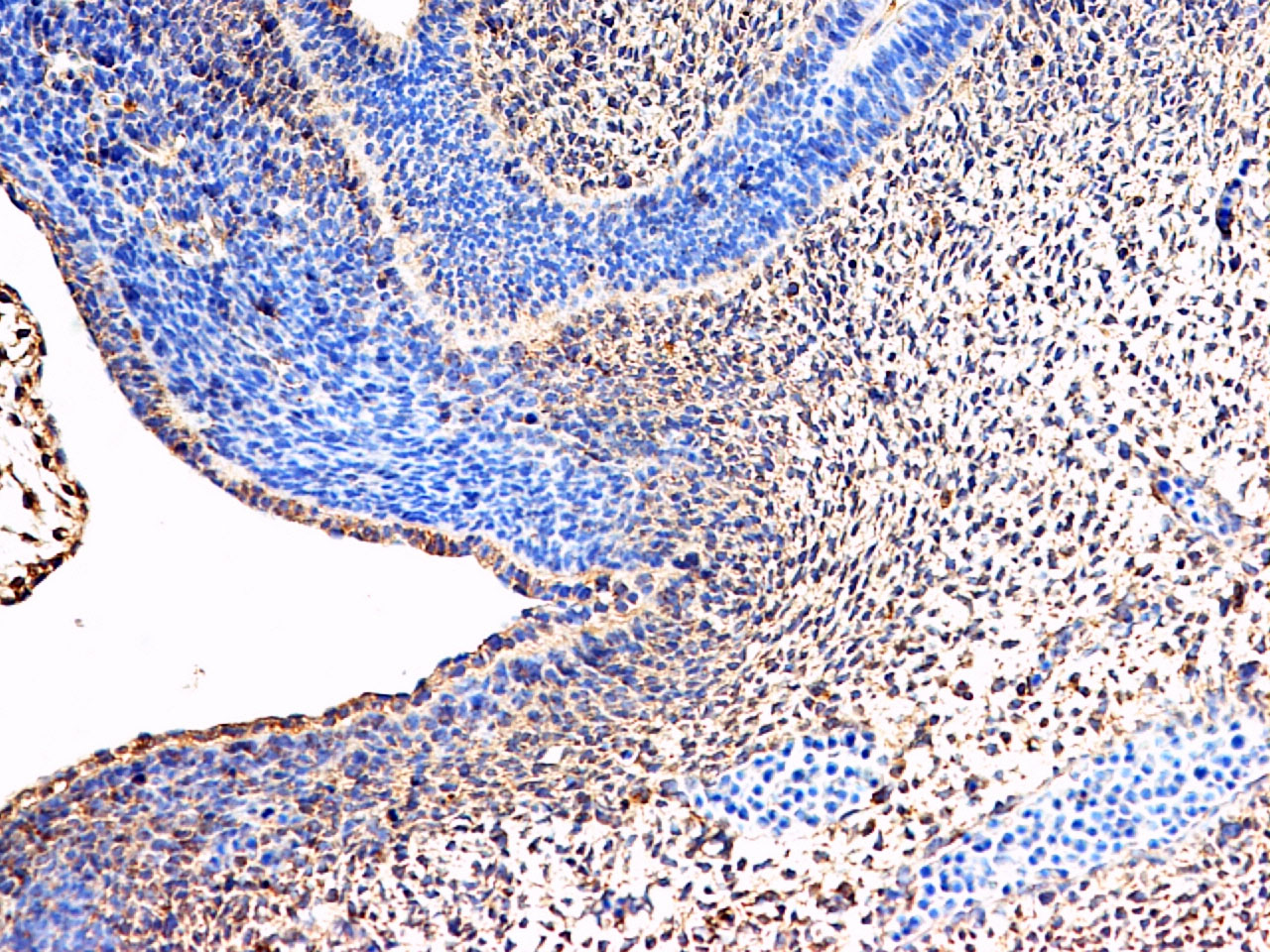

Supplement: Supplemental Information 1 [file peerj-04-1771-s001.zip › 1/c1-25-10 200'╡≈╒√.jpg]

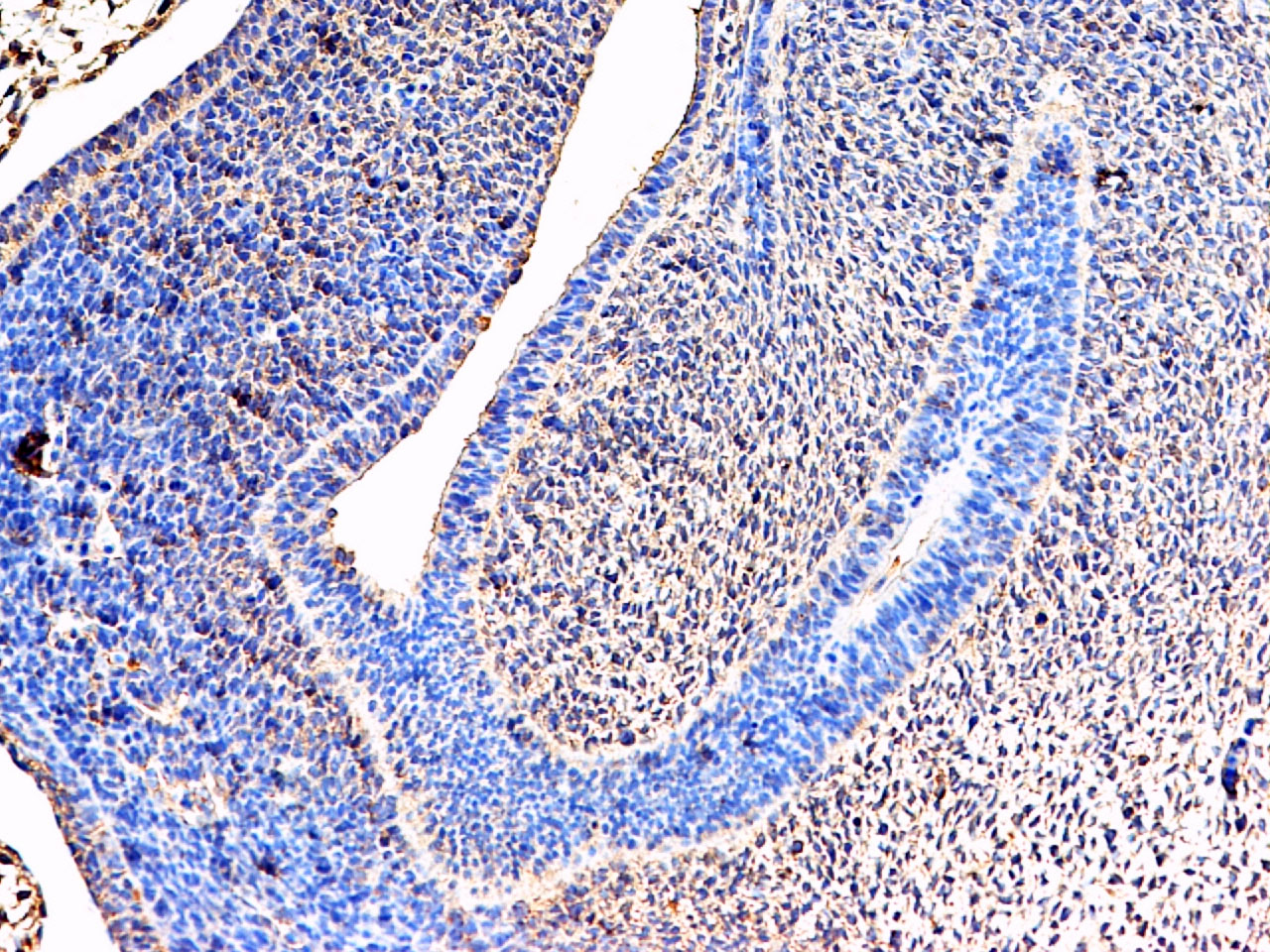

Supplement: Supplemental Information 1 [file peerj-04-1771-s001.zip › 1/c1-25-10 200╡≈╒√.jpg]

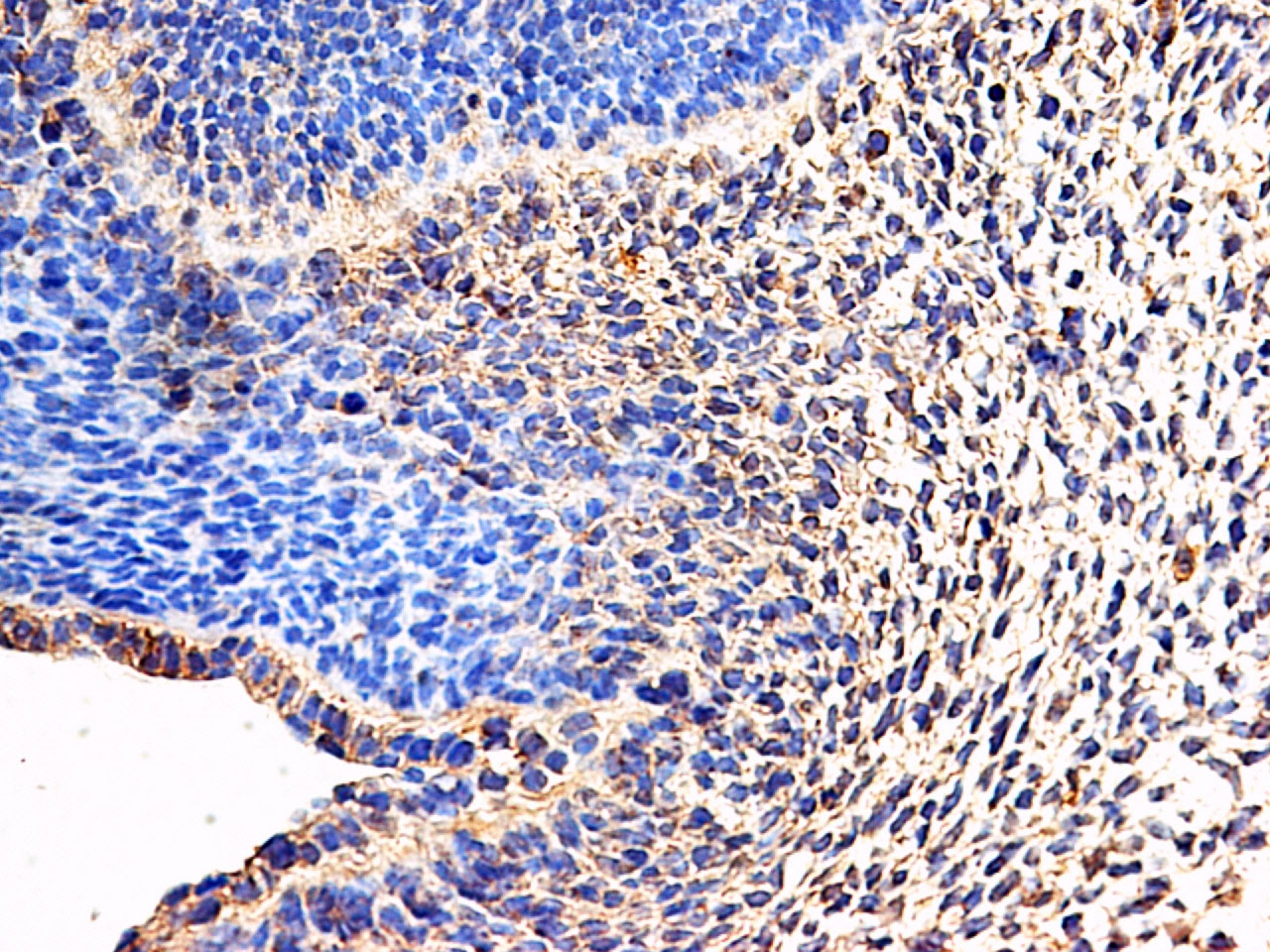

Supplement: Supplemental Information 1 [file peerj-04-1771-s001.zip › 1/c1-25-10 400╡≈╒√.jpg]

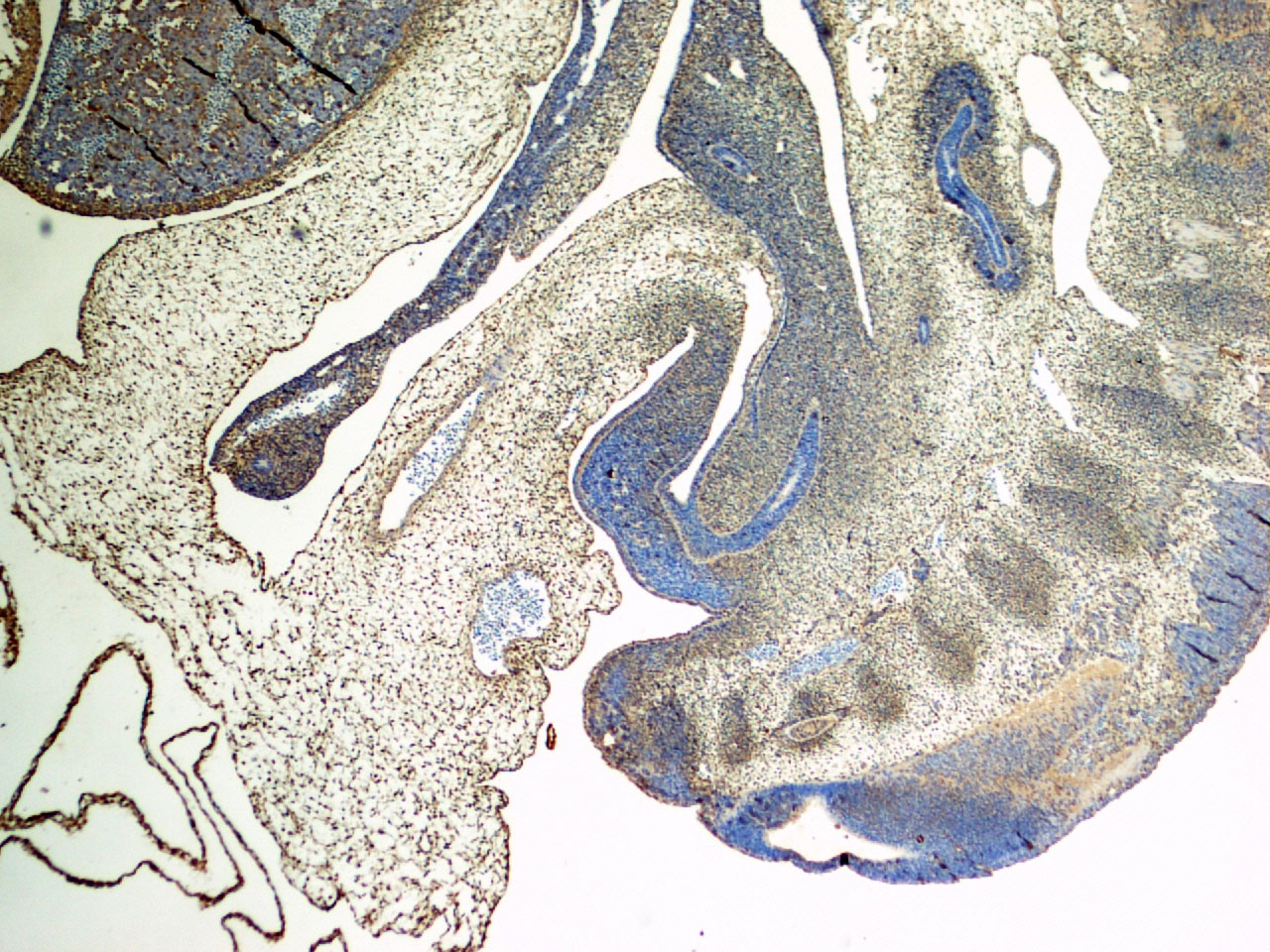

Supplement: Supplemental Information 1 [file peerj-04-1771-s001.zip › 1/c1-25-10 40╡≈╒√.jpg]

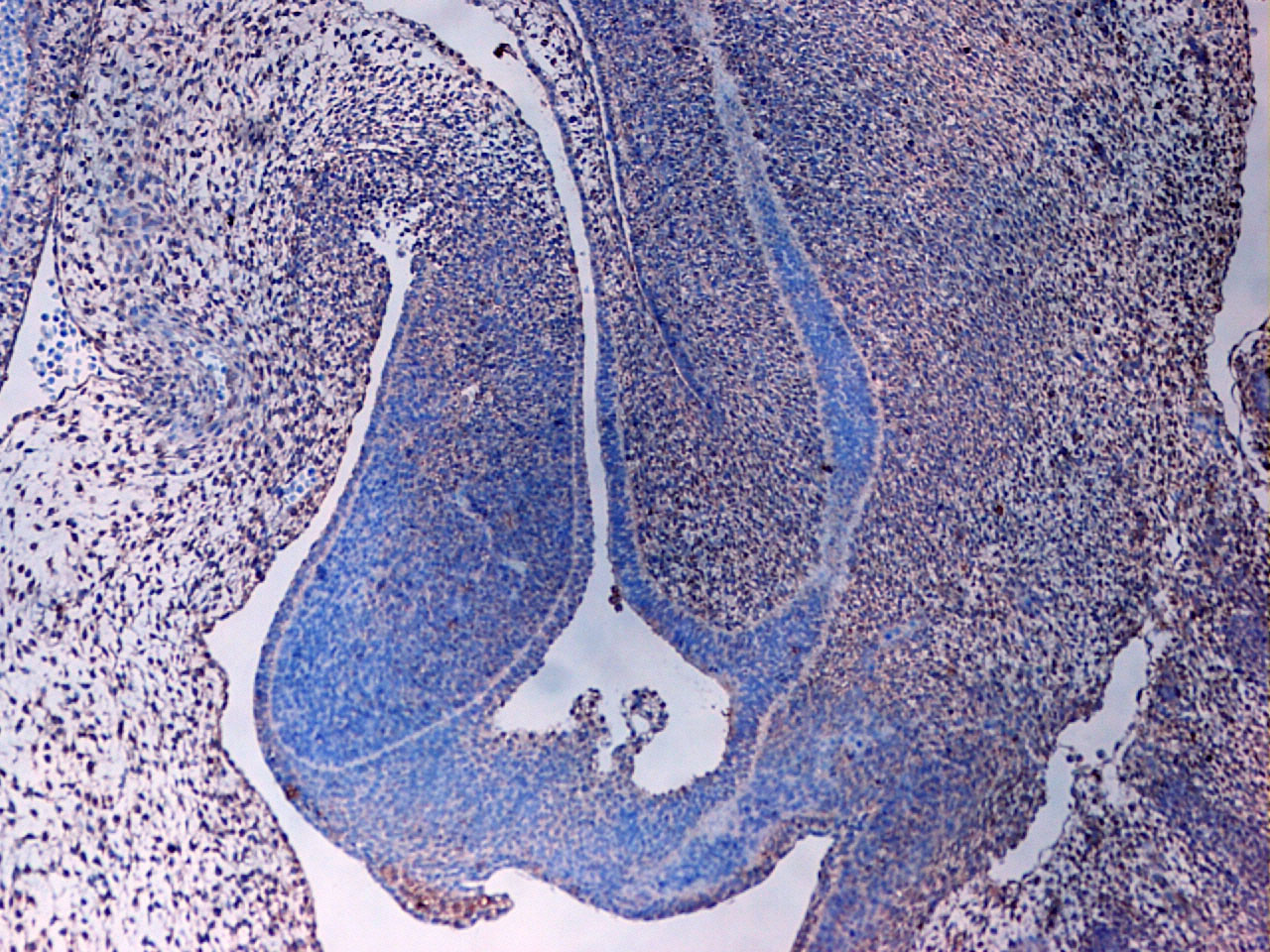

Supplement: Supplemental Information 1 [file peerj-04-1771-s001.zip › 1/C1-37-14 100▒╢╡≈╒√'.jpg]

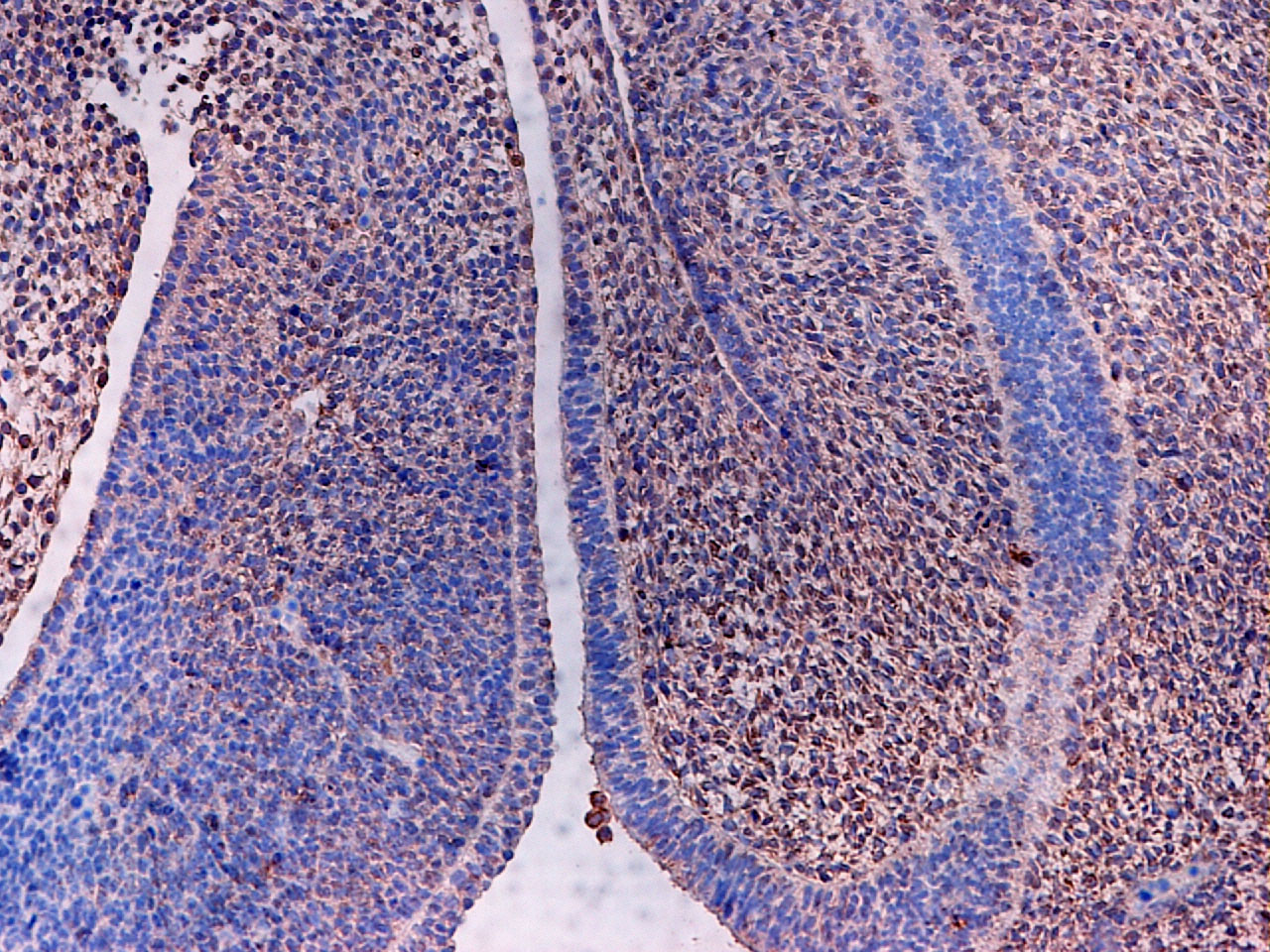

Supplement: Supplemental Information 1 [file peerj-04-1771-s001.zip › 1/C1-37-14 200▒╢'╡≈╒√.jpg]

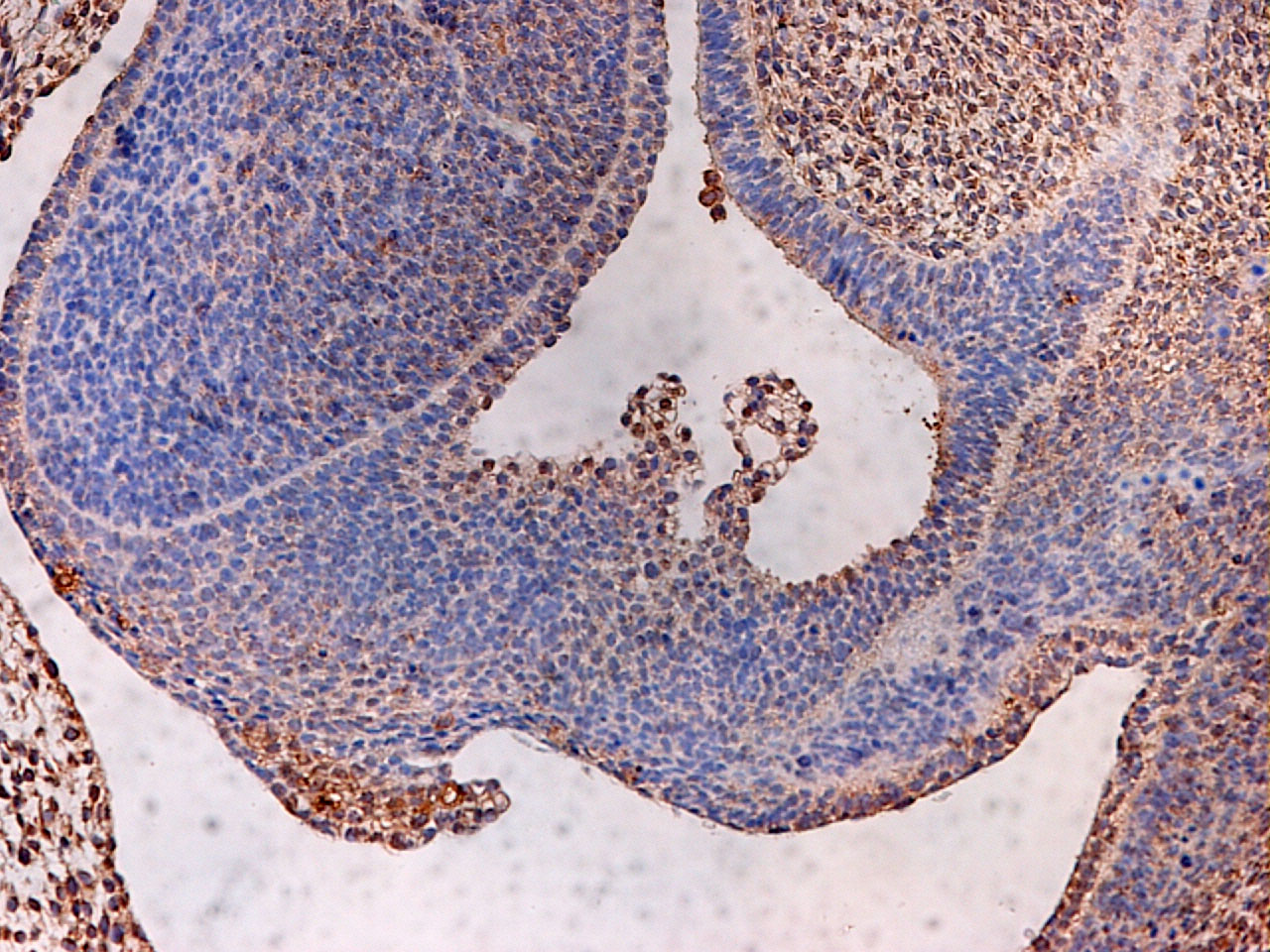

Supplement: Supplemental Information 1 [file peerj-04-1771-s001.zip › 1/C1-37-14 200▒╢╡≈╒√.jpg]

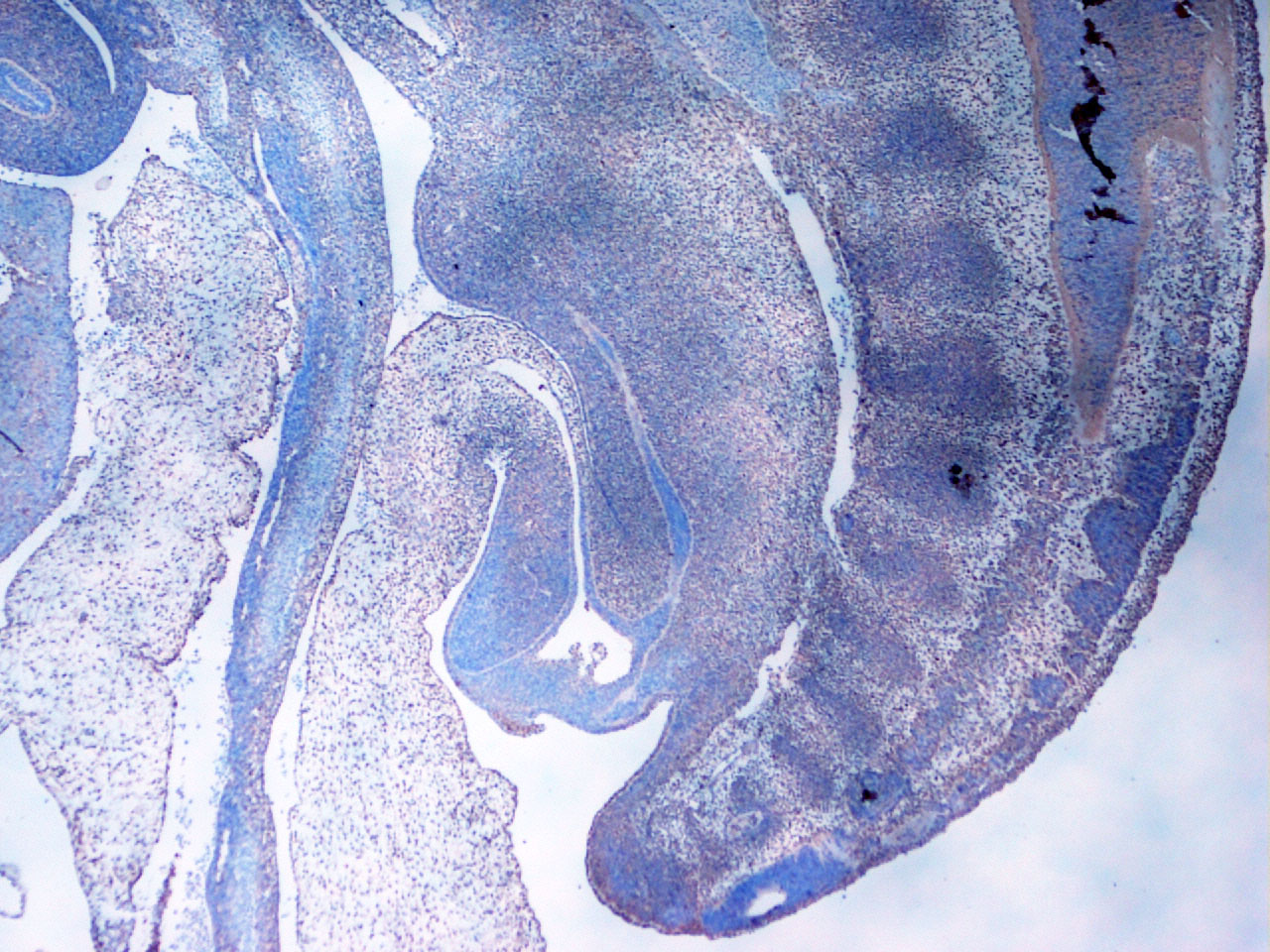

Supplement: Supplemental Information 1 [file peerj-04-1771-s001.zip › 1/C1-37-14 40▒╢╡≈╒√'.jpg]

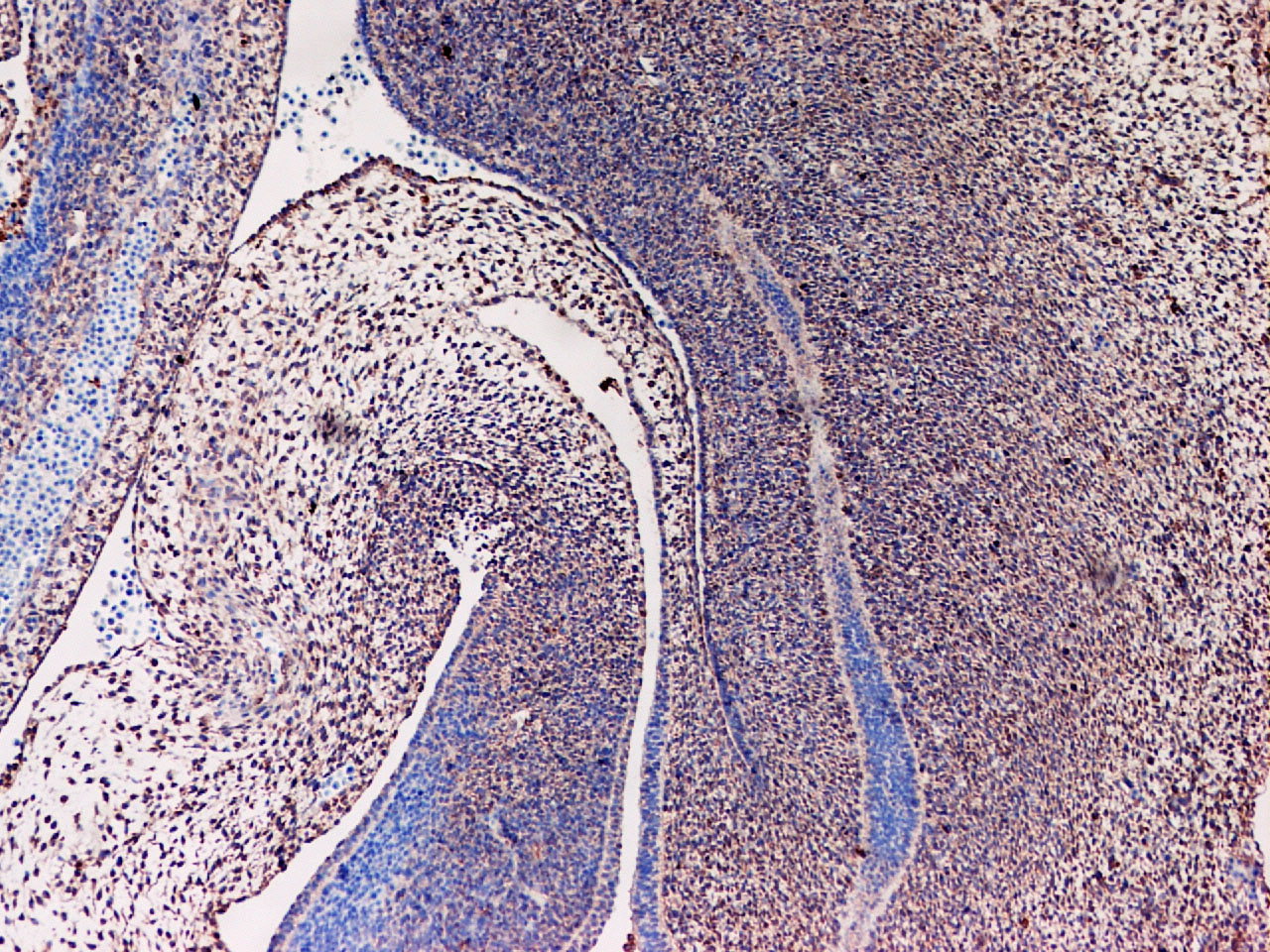

Supplement: Supplemental Information 1 [file peerj-04-1771-s001.zip › 1/c1-37-14-100▒╢'╡≈╒√.jpg]

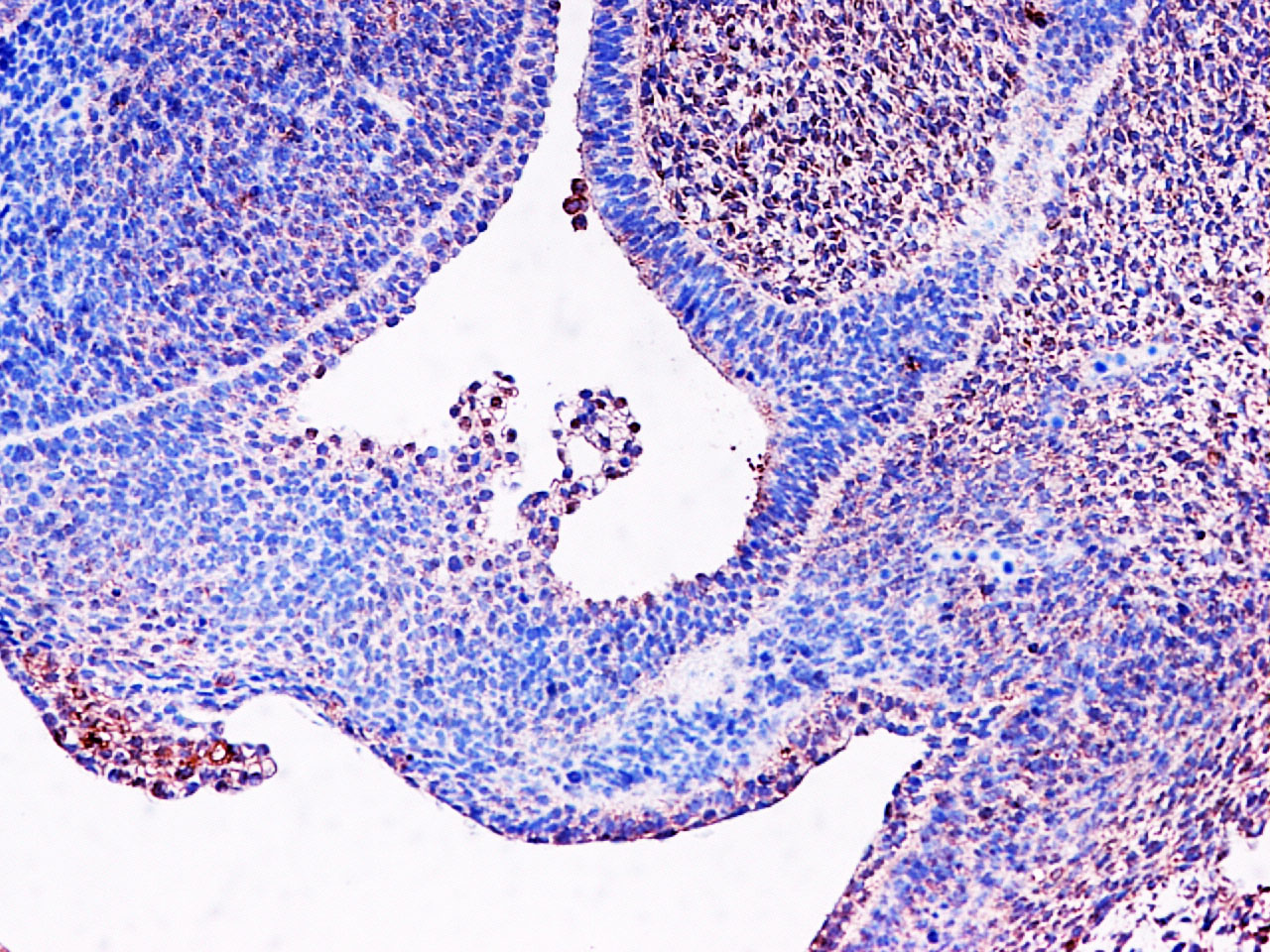

Supplement: Supplemental Information 1 [file peerj-04-1771-s001.zip › 1/c1-37-14-200▒╢'╡≈╒√.jpg]

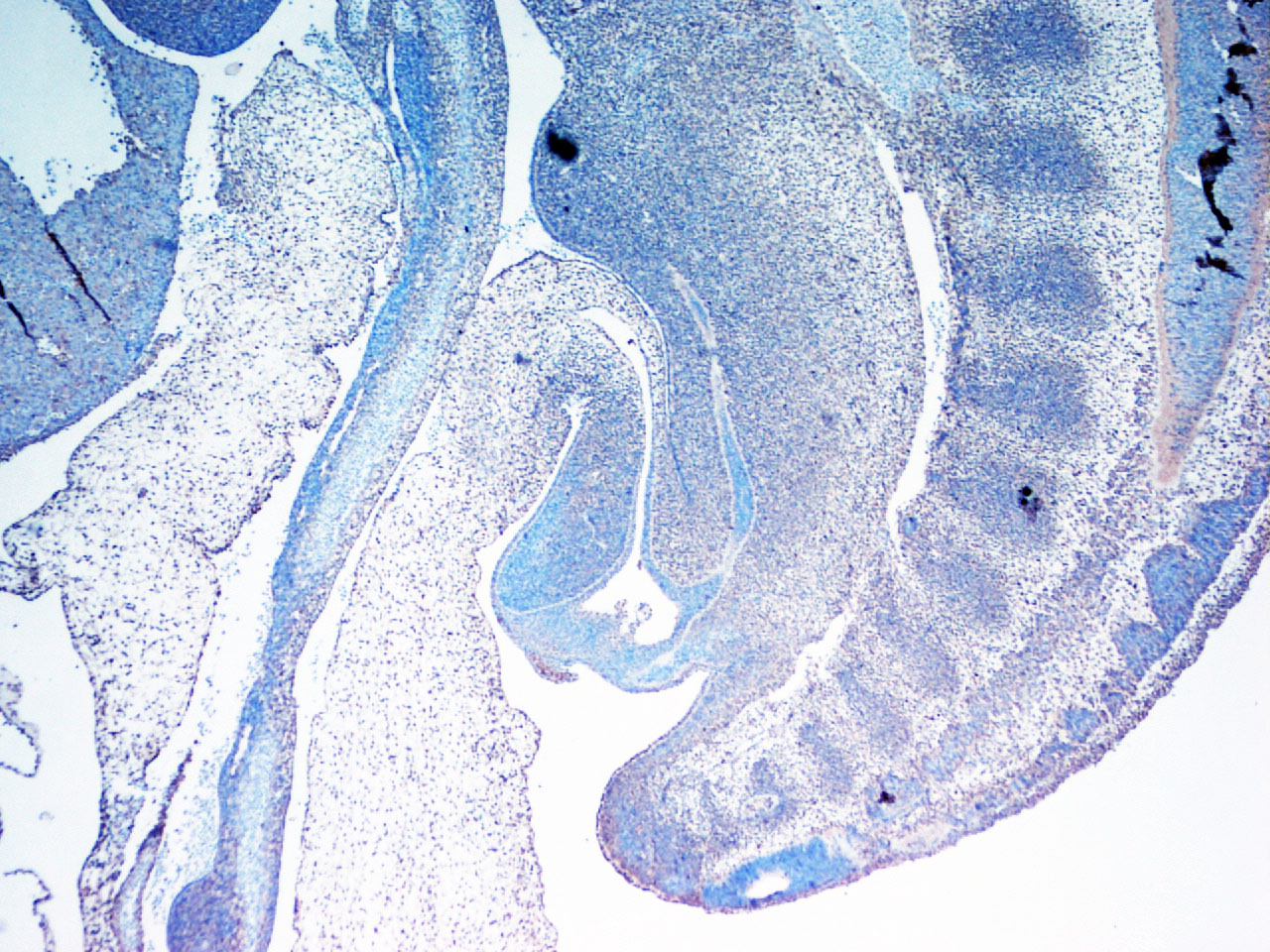

Supplement: Supplemental Information 1 [file peerj-04-1771-s001.zip › 1/c1-37-14-40▒╢╡≈╒√.jpg]

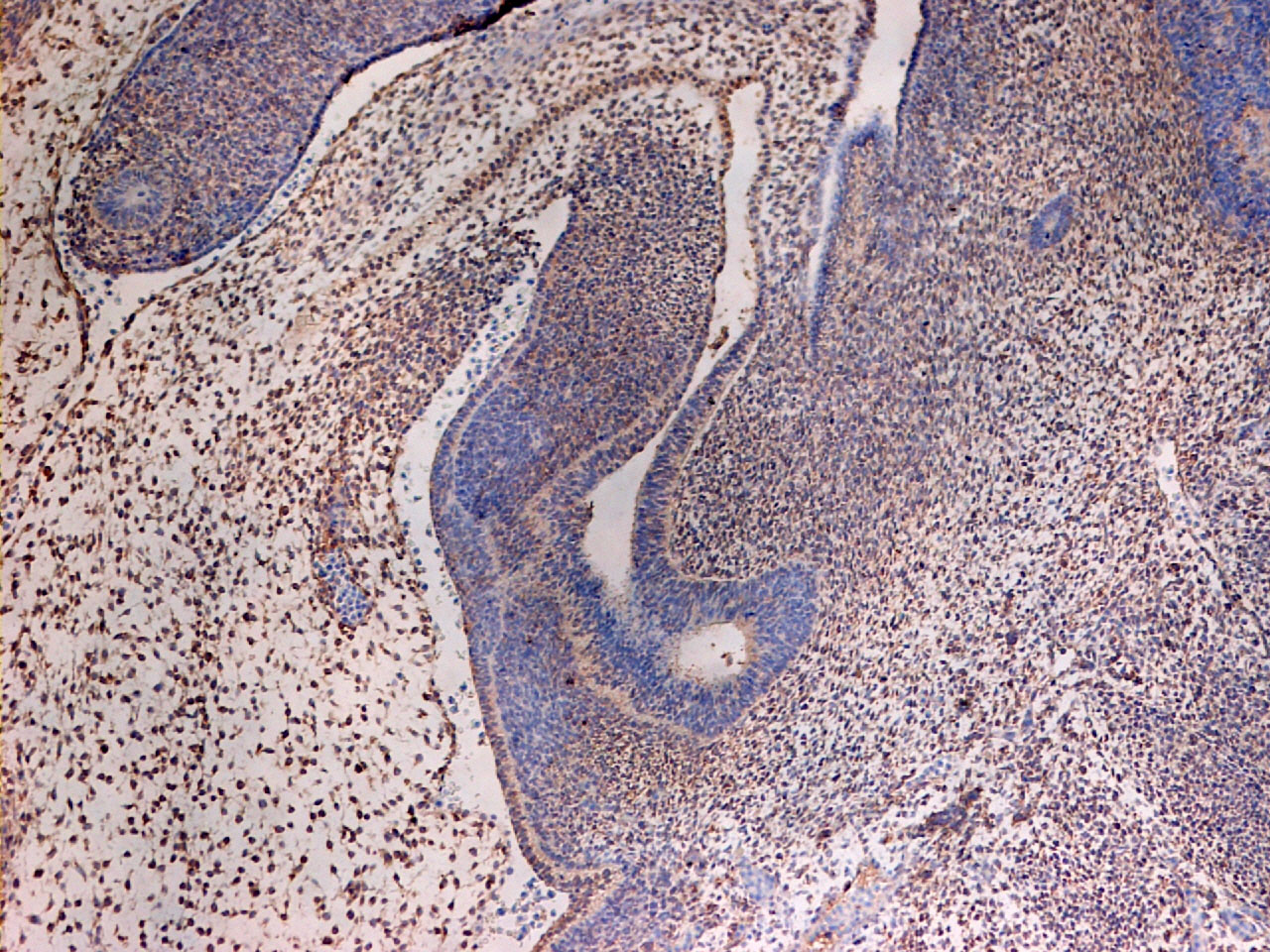

Supplement: Supplemental Information 1 [file peerj-04-1771-s001.zip › 1/c1-38-11 100▒╢╡≈╒√.jpg]

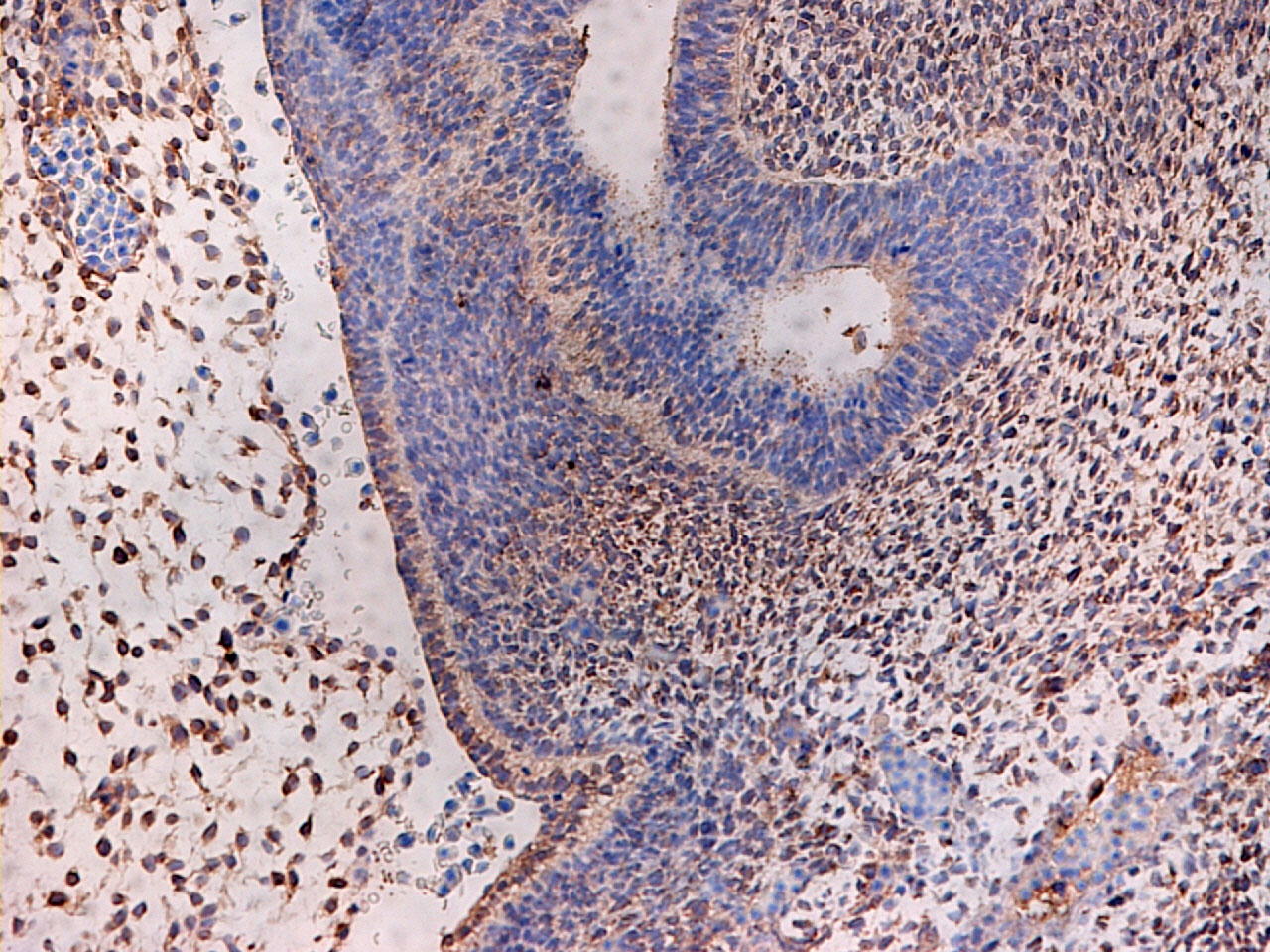

Supplement: Supplemental Information 1 [file peerj-04-1771-s001.zip › 1/c1-38-11 200▒╢'╡≈╒√.jpg]

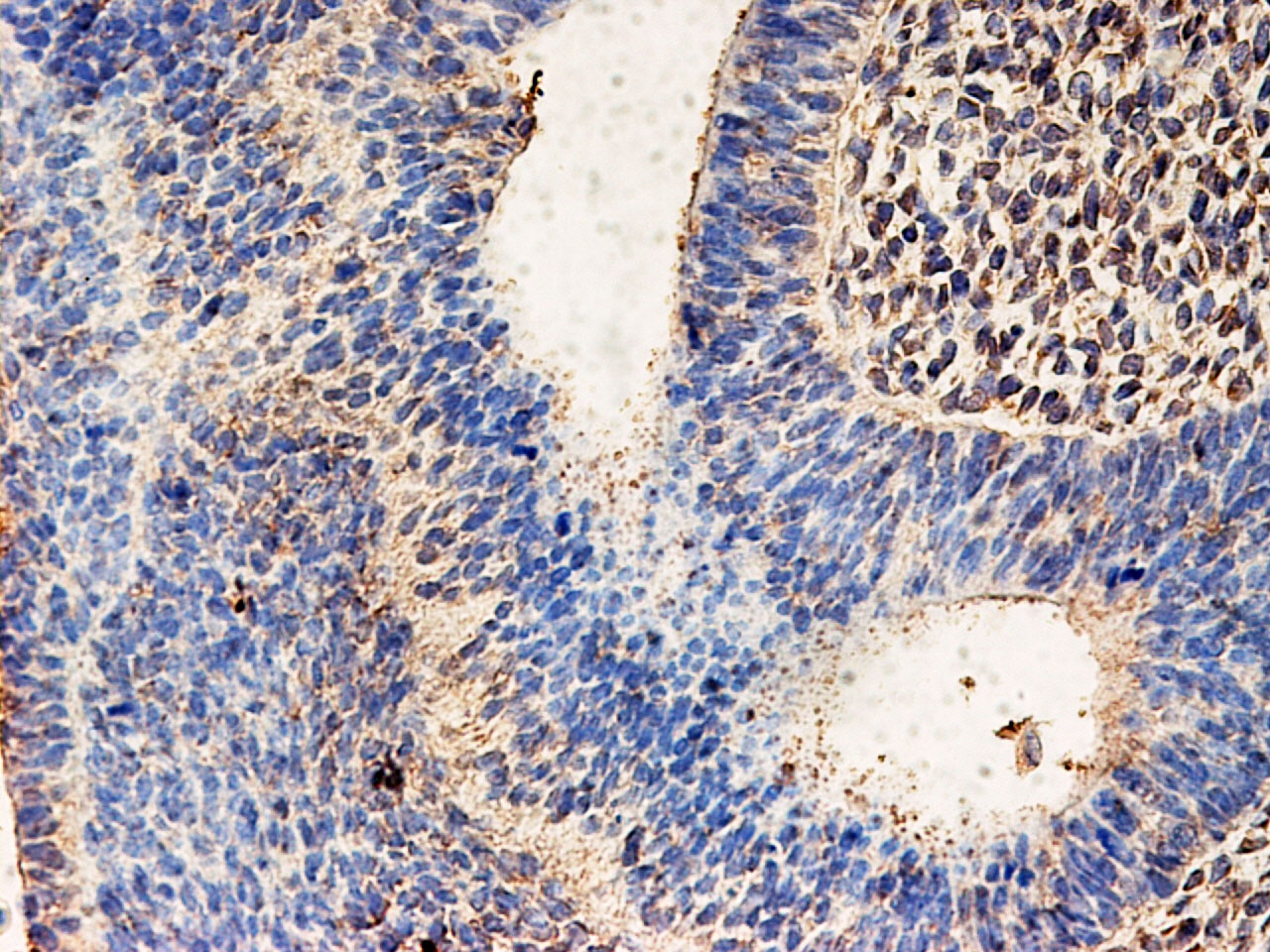

Supplement: Supplemental Information 1 [file peerj-04-1771-s001.zip › 1/c1-38-11-400▒╢'╡≈╒√.jpg]

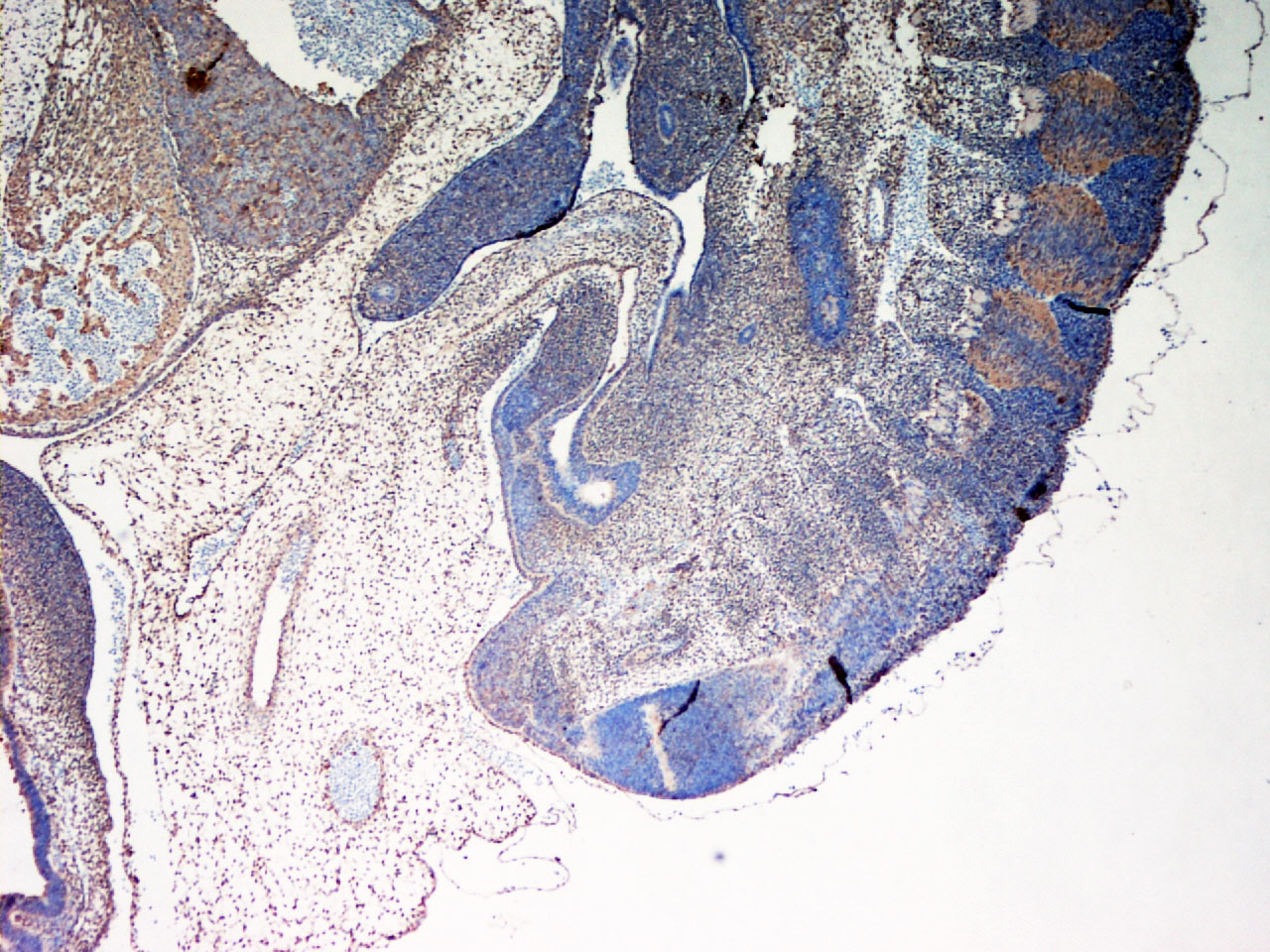

Supplement: Supplemental Information 1 [file peerj-04-1771-s001.zip › 1/c1-38-11-40▒╢╡≈╒√.jpg]

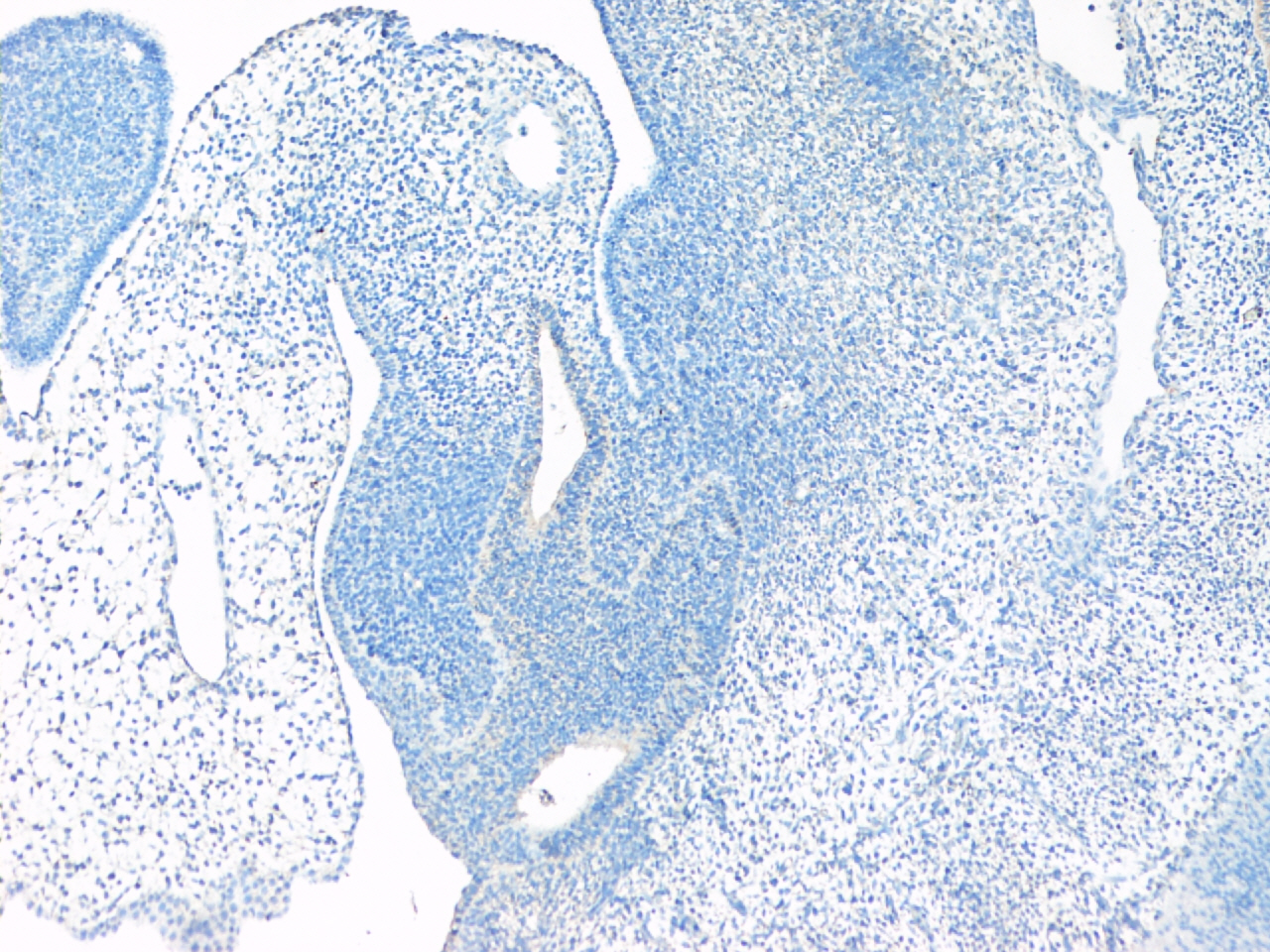

Supplement: Supplemental Information 1 [file peerj-04-1771-s001.zip › 1/c1-4-1 100.jpg]

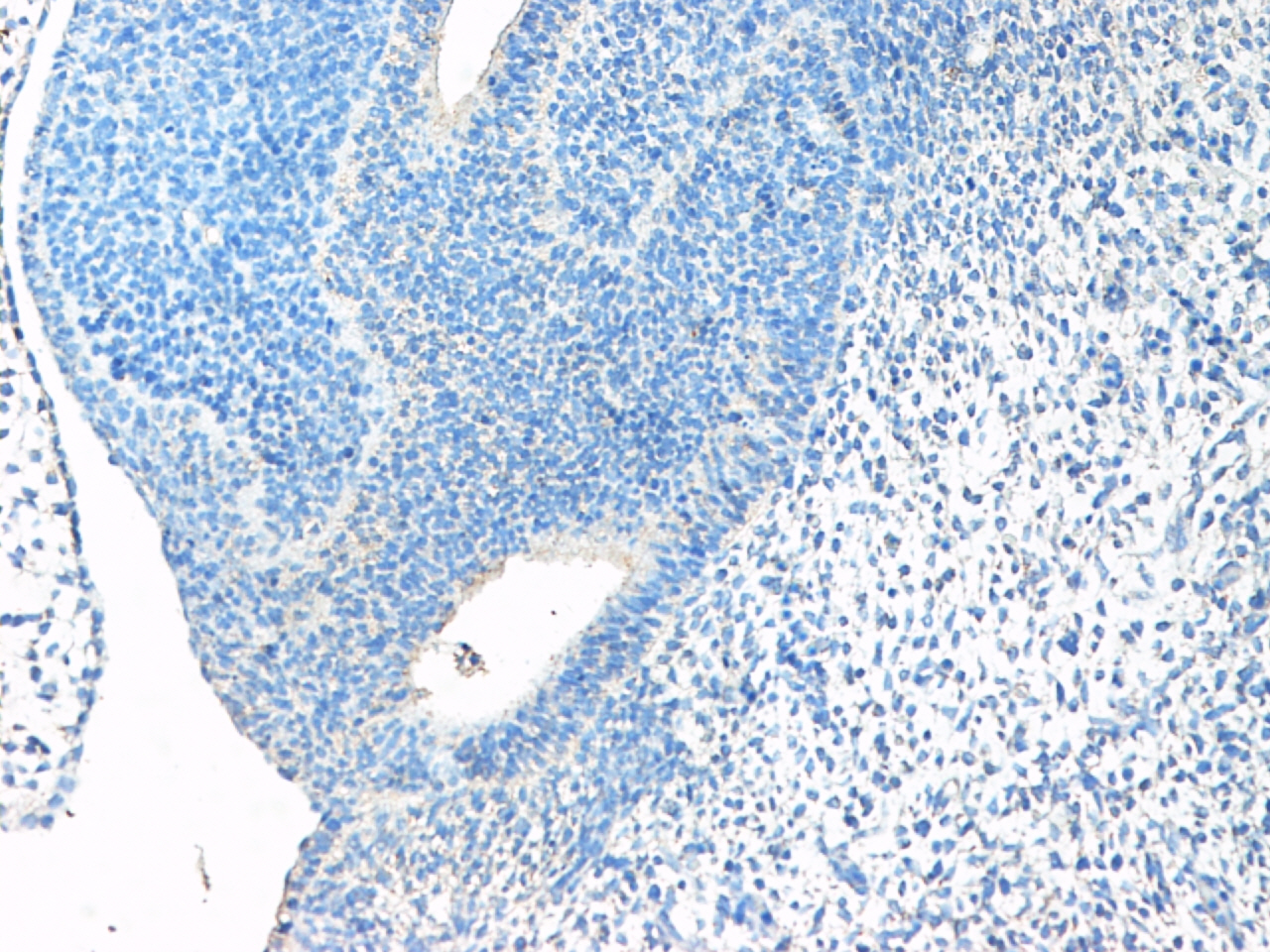

Supplement: Supplemental Information 1 [file peerj-04-1771-s001.zip › 1/c1-4-1 200.jpg]

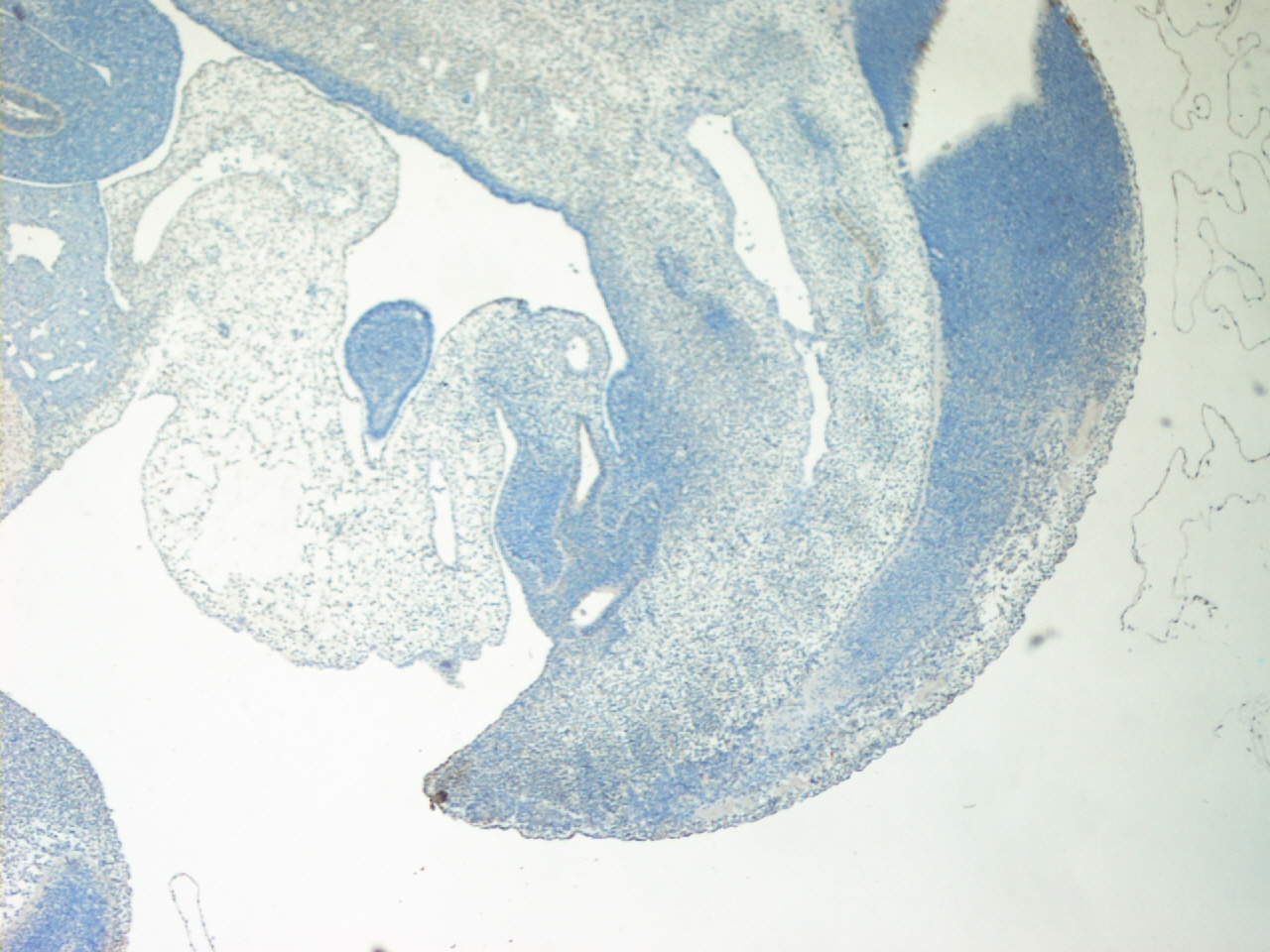

Supplement: Supplemental Information 1 [file peerj-04-1771-s001.zip › 1/c1-4-1 40.jpg]

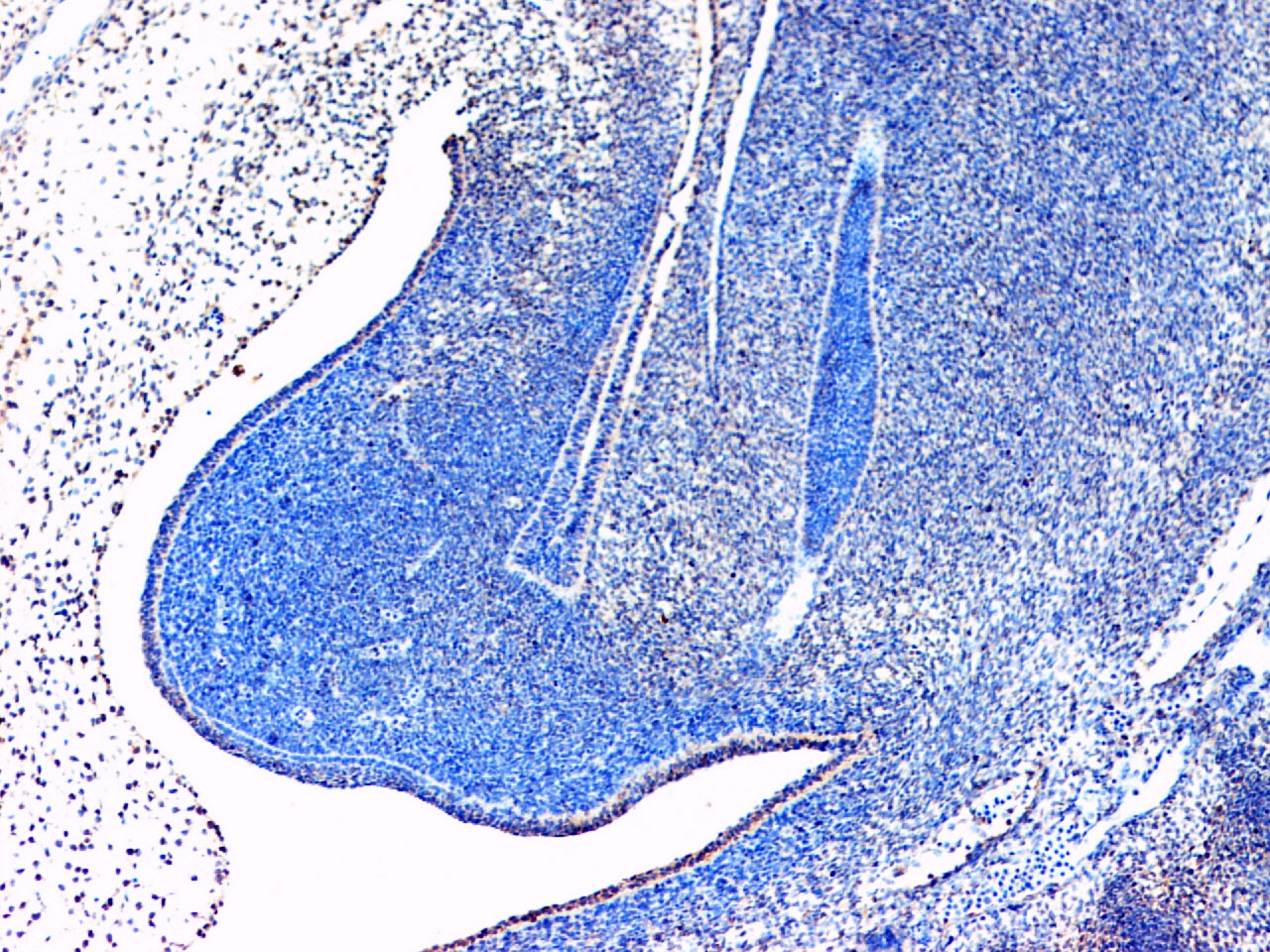

Supplement: Supplemental Information 1 [file peerj-04-1771-s001.zip › 1/c1-8-1 100╡≈╒√.jpg]

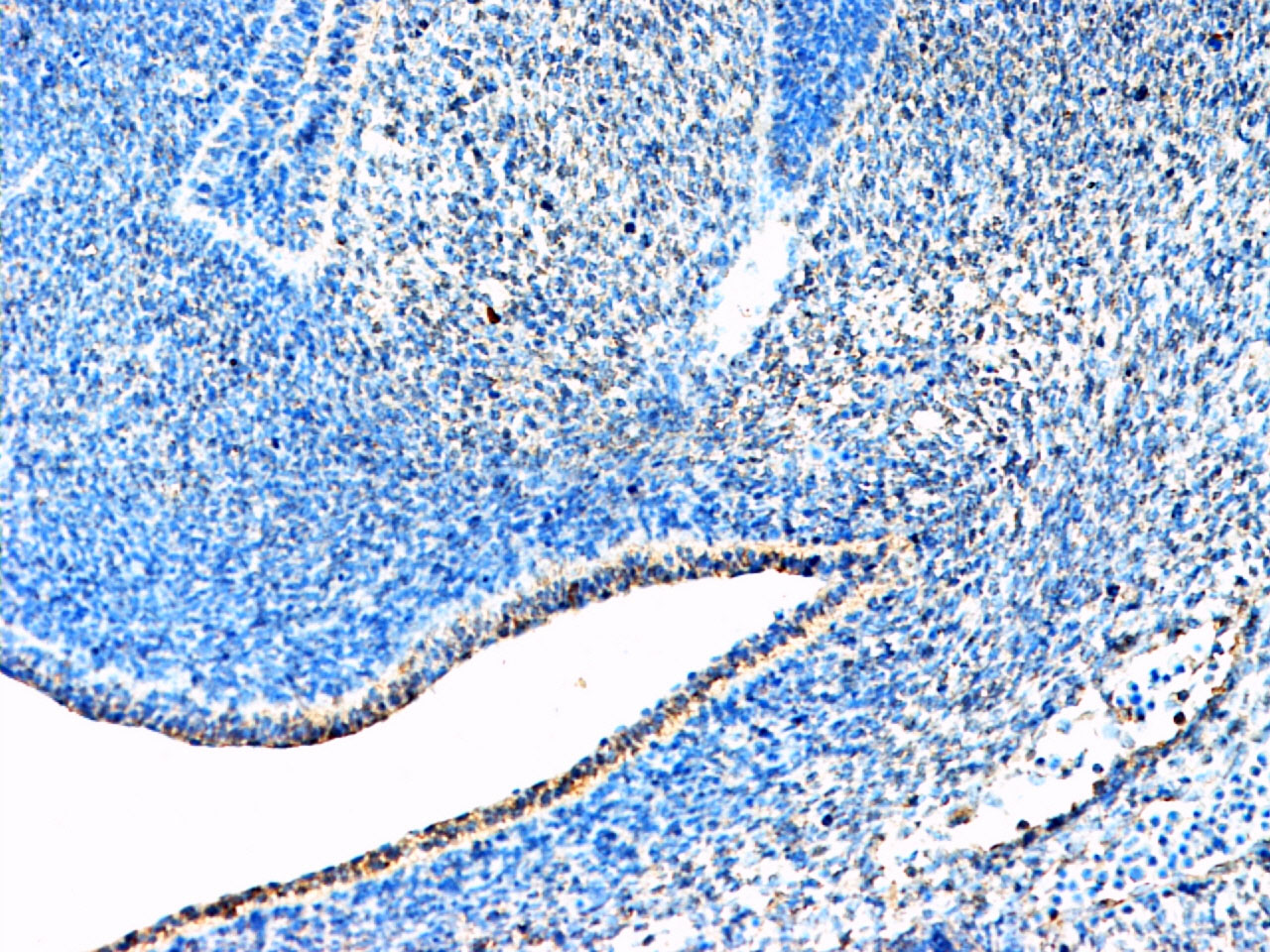

Supplement: Supplemental Information 1 [file peerj-04-1771-s001.zip › 1/c1-8-1 200'╡≈╒√.jpg]

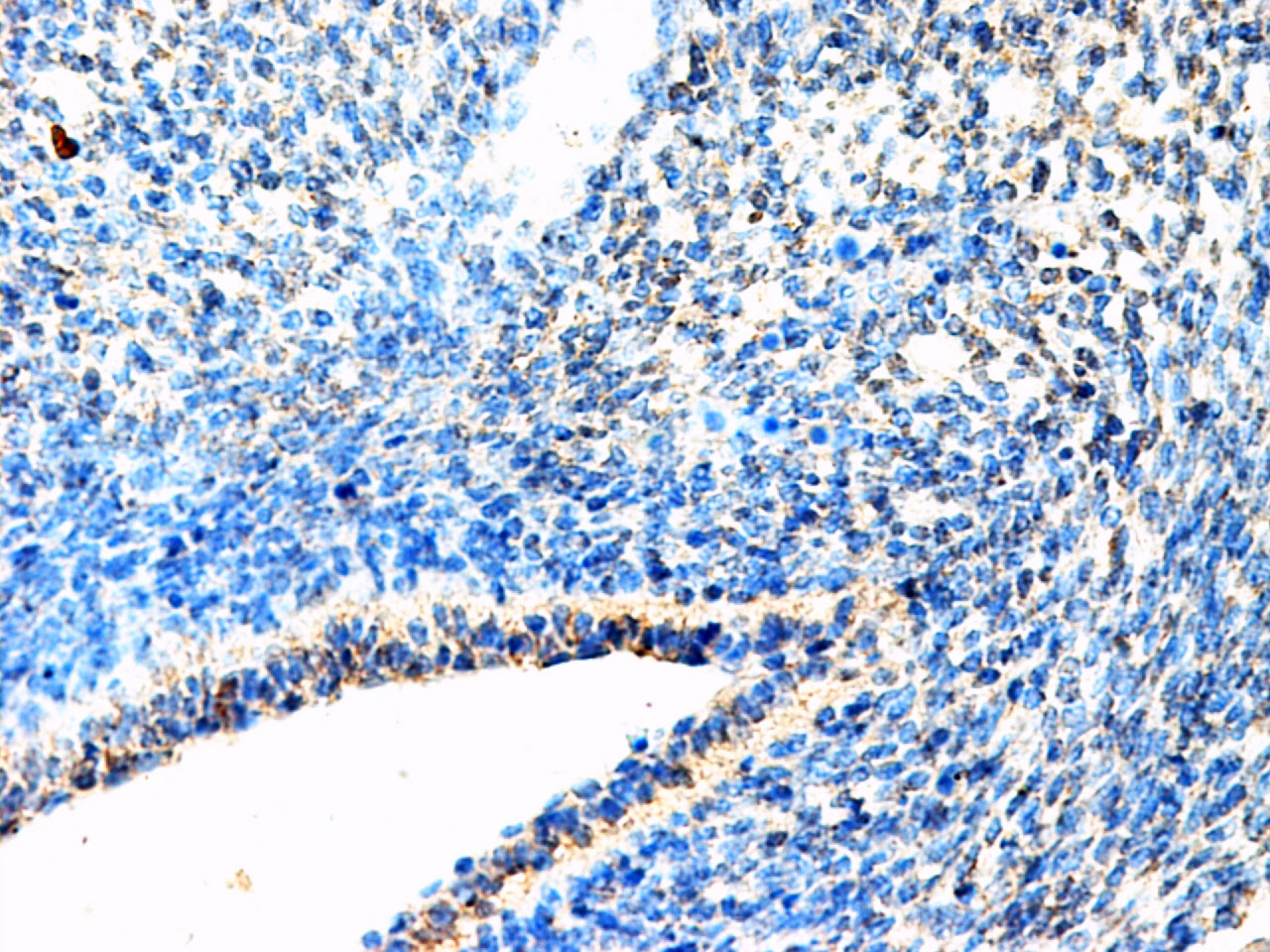

Supplement: Supplemental Information 1 [file peerj-04-1771-s001.zip › 1/c1-8-1 400'╡≈╒√.jpg]

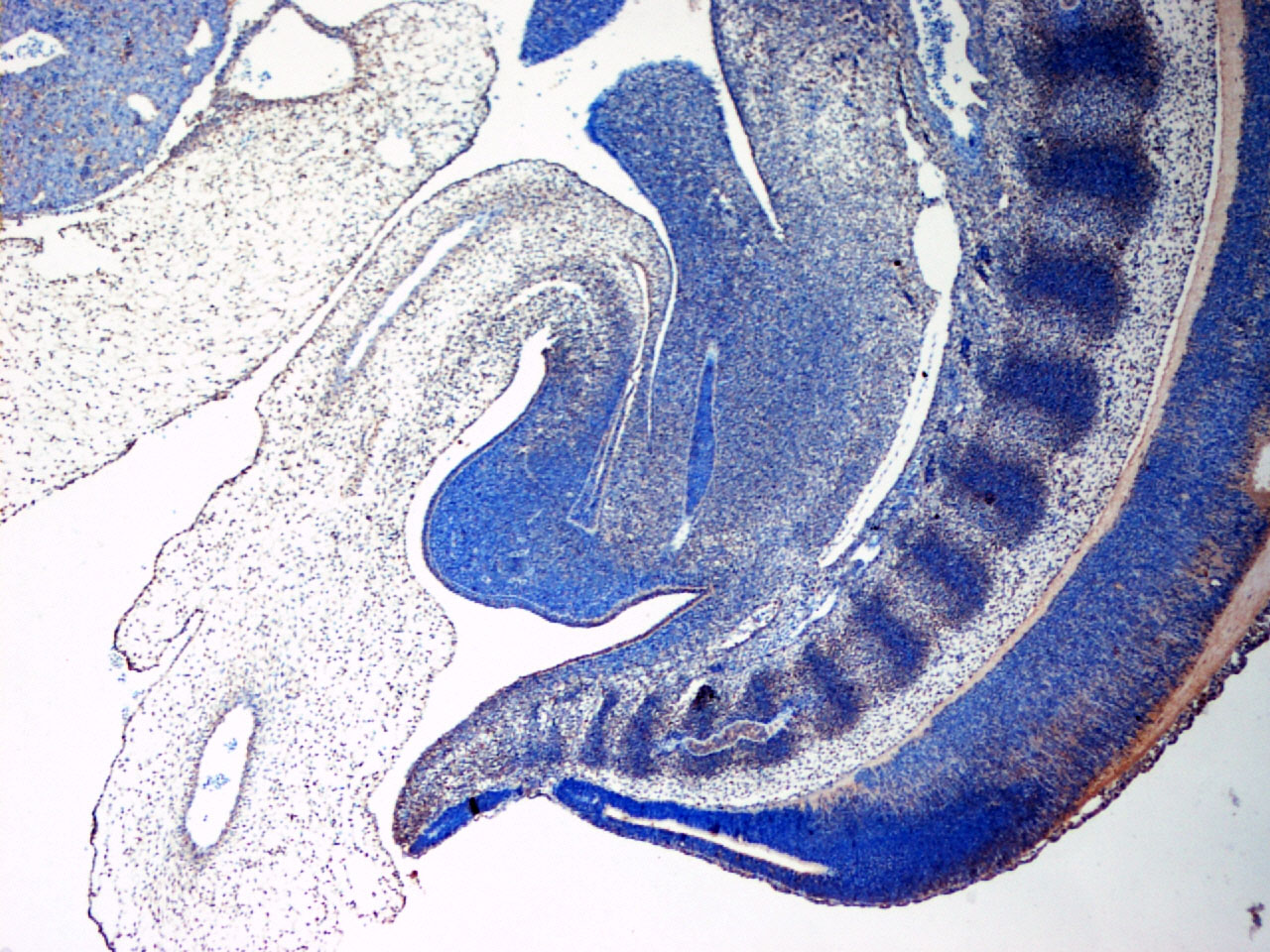

Supplement: Supplemental Information 1 [file peerj-04-1771-s001.zip › 1/c1-8-1 40╡≈╒√.jpg]

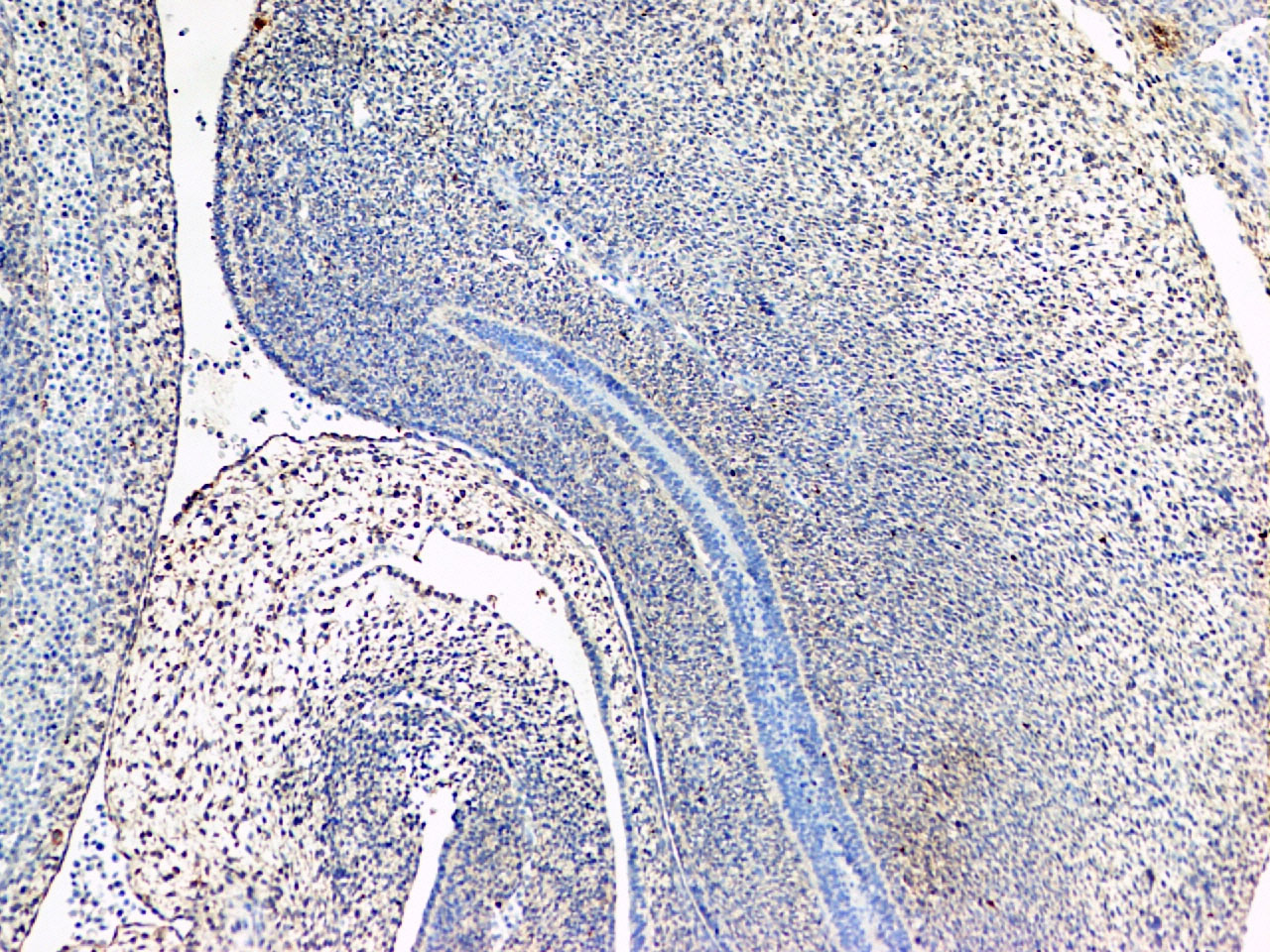

Supplement: Supplemental Information 2 [file peerj-04-1771-s002.zip › 2/c2-37-10 100'╡≈╒√.jpg]

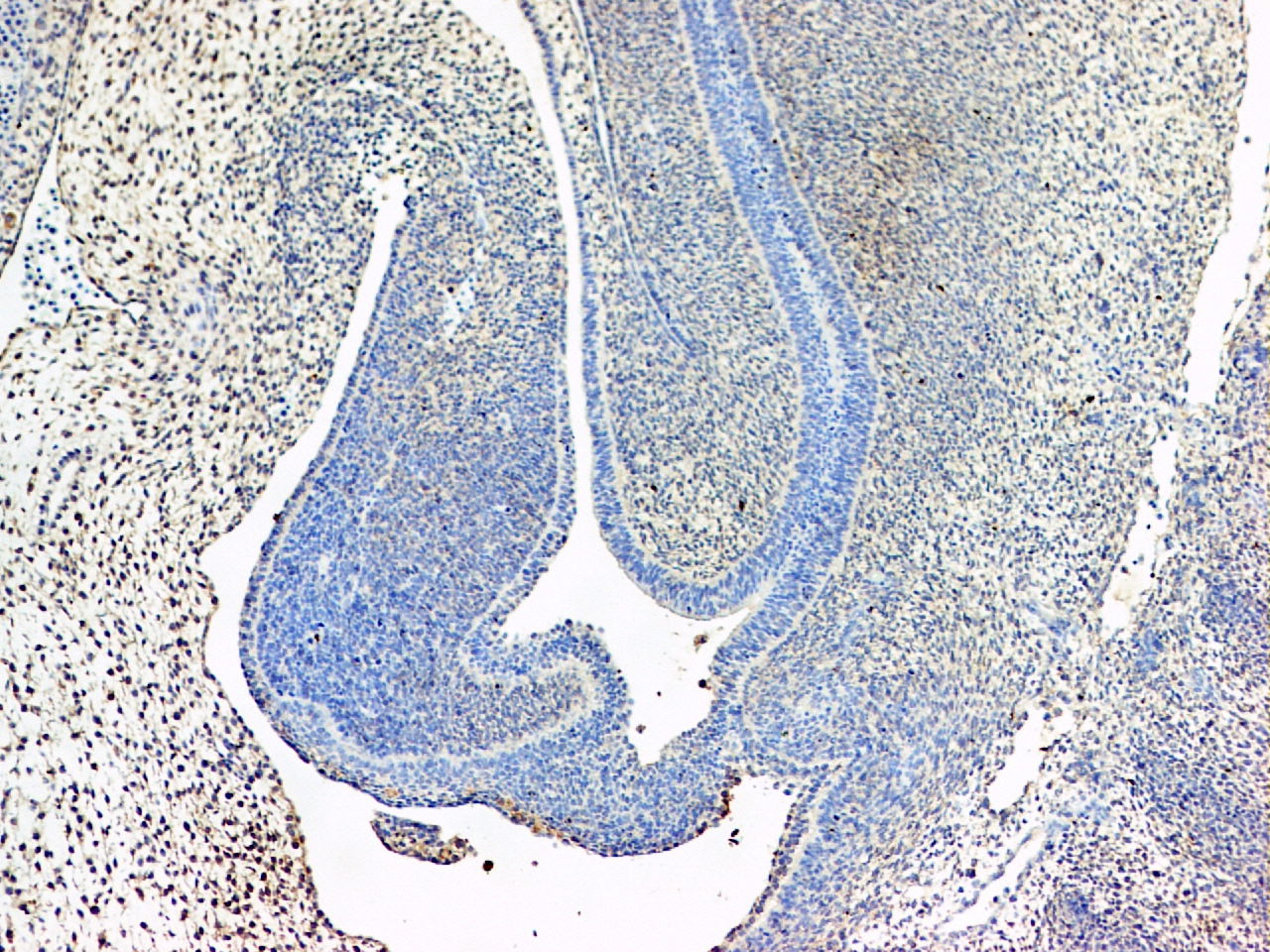

Supplement: Supplemental Information 2 [file peerj-04-1771-s002.zip › 2/c2-37-10 100╡≈╒√.jpg]

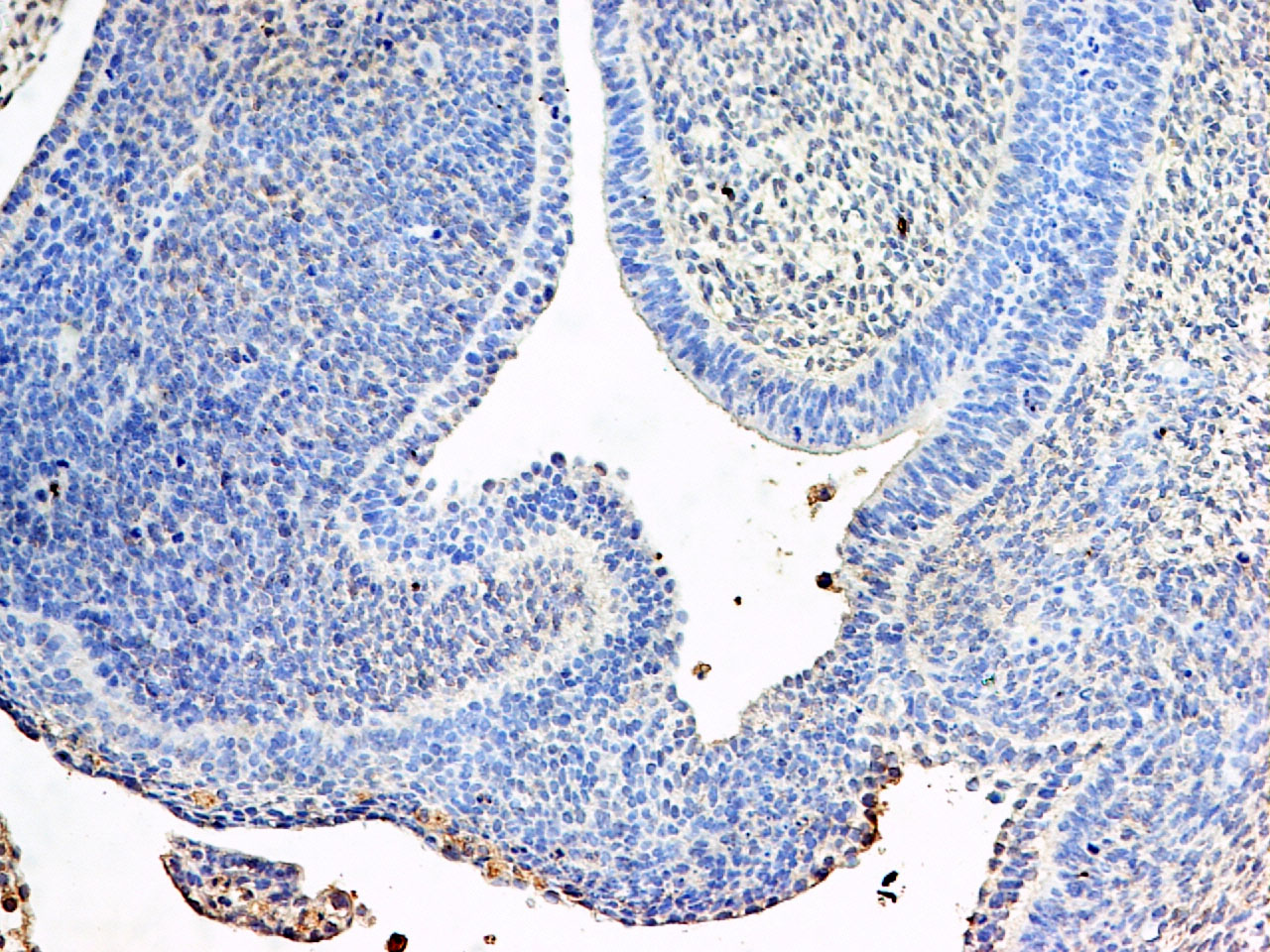

Supplement: Supplemental Information 2 [file peerj-04-1771-s002.zip › 2/c2-37-10 200''╡≈╒√.jpg]

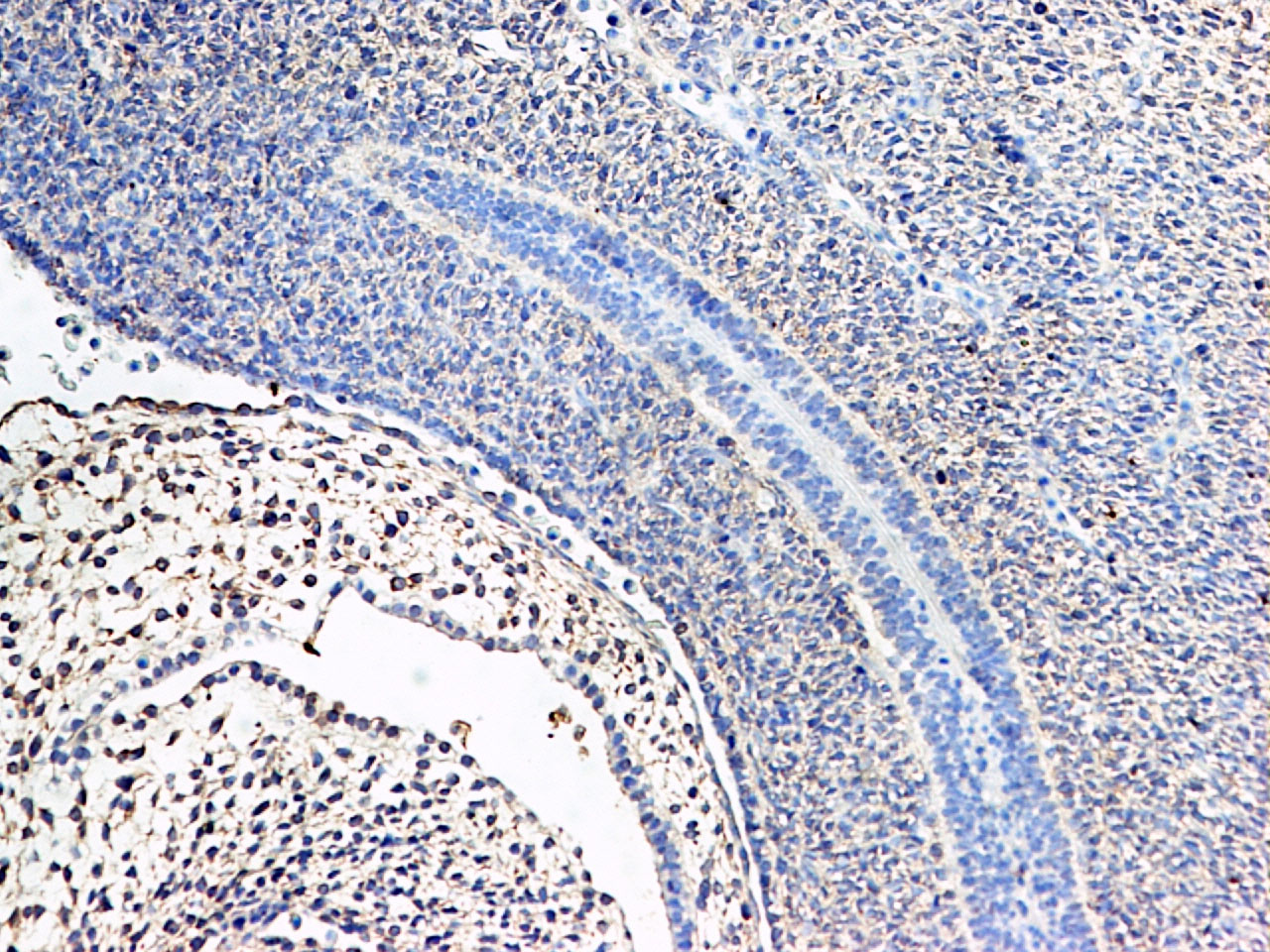

Supplement: Supplemental Information 2 [file peerj-04-1771-s002.zip › 2/c2-37-10 200╡≈╒√.jpg]

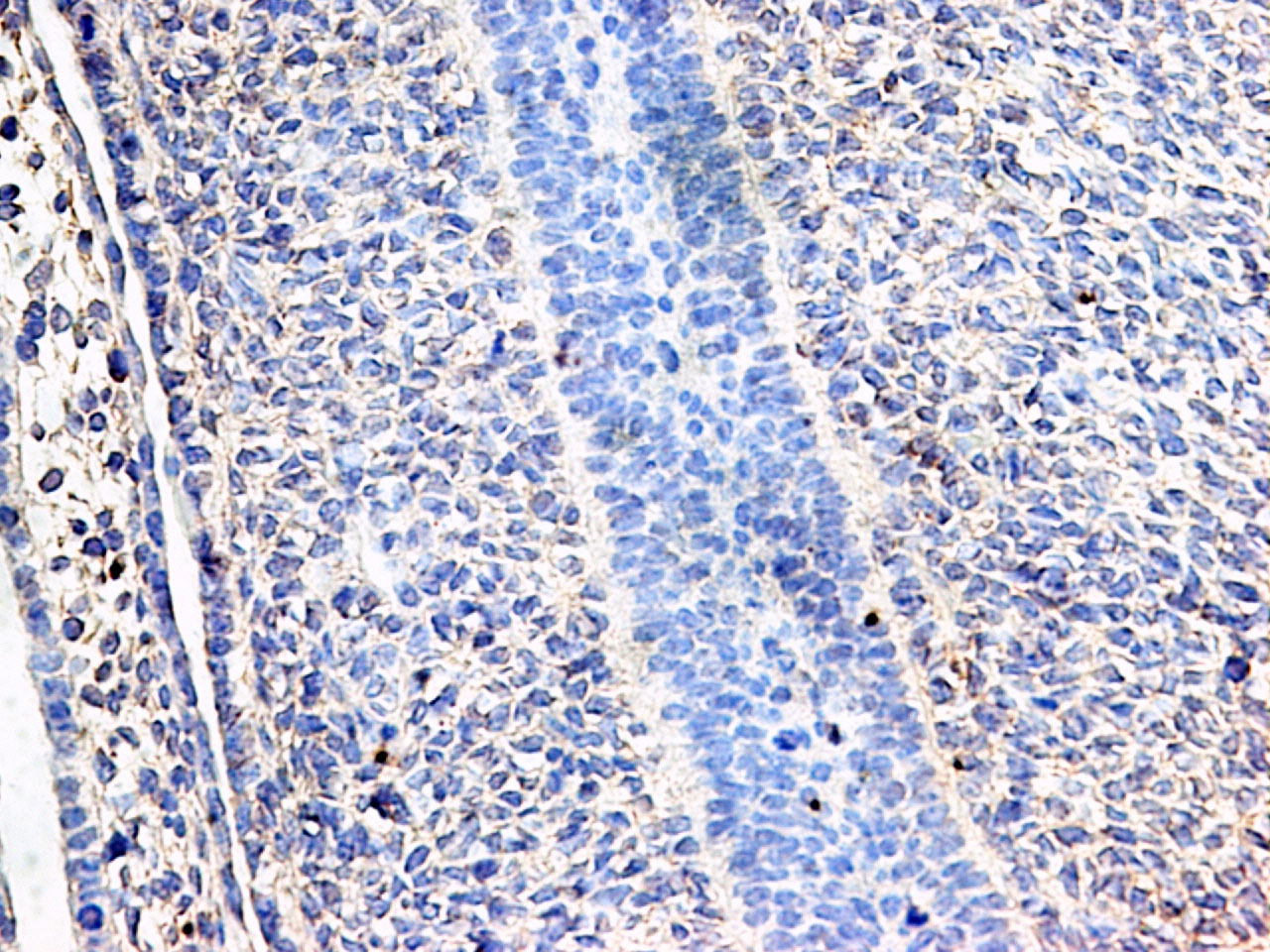

Supplement: Supplemental Information 2 [file peerj-04-1771-s002.zip › 2/c2-37-10 400╡≈╒√.jpg]

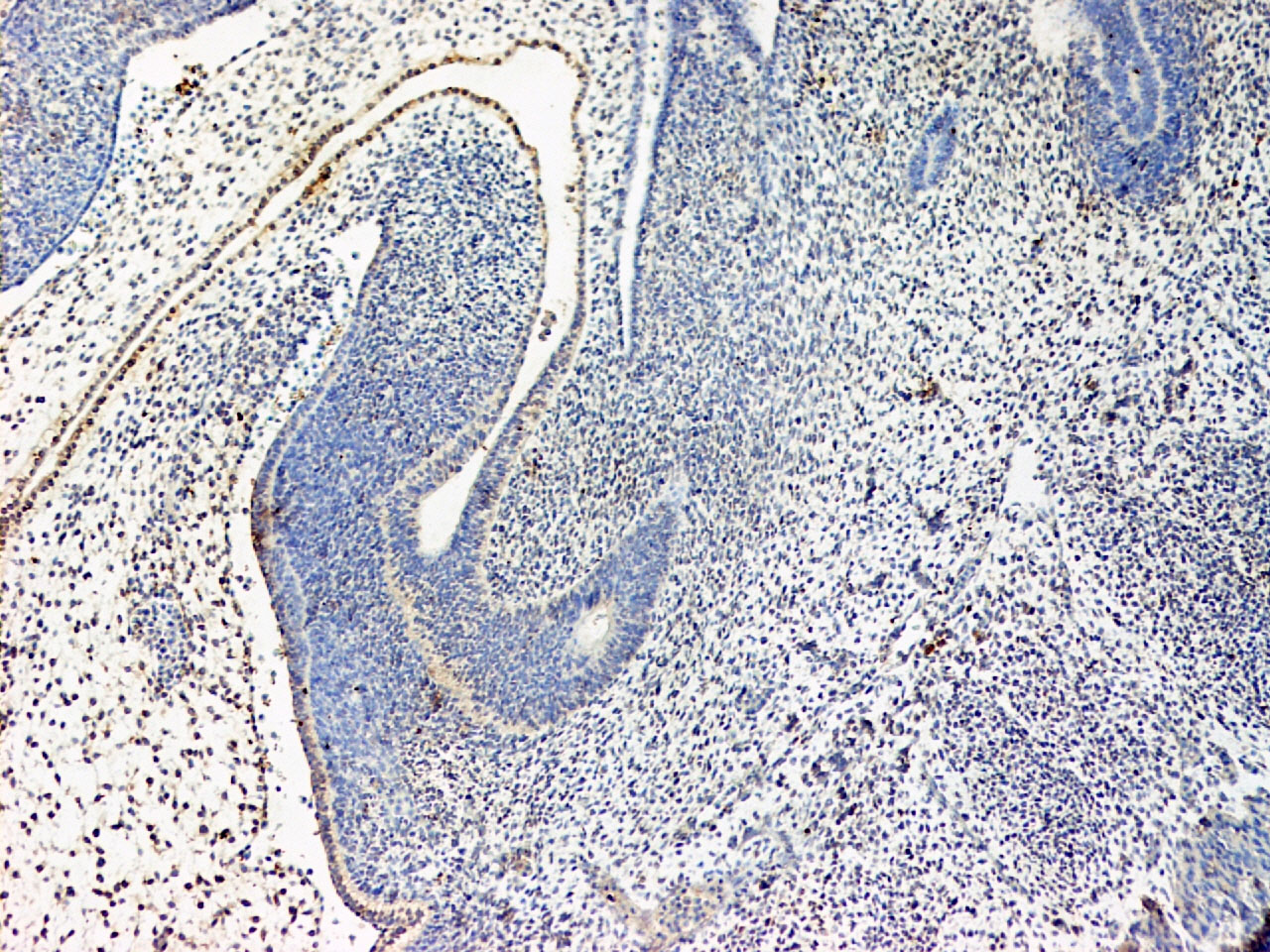

Supplement: Supplemental Information 2 [file peerj-04-1771-s002.zip › 2/c2-38-10 100╡≈╒√.jpg]

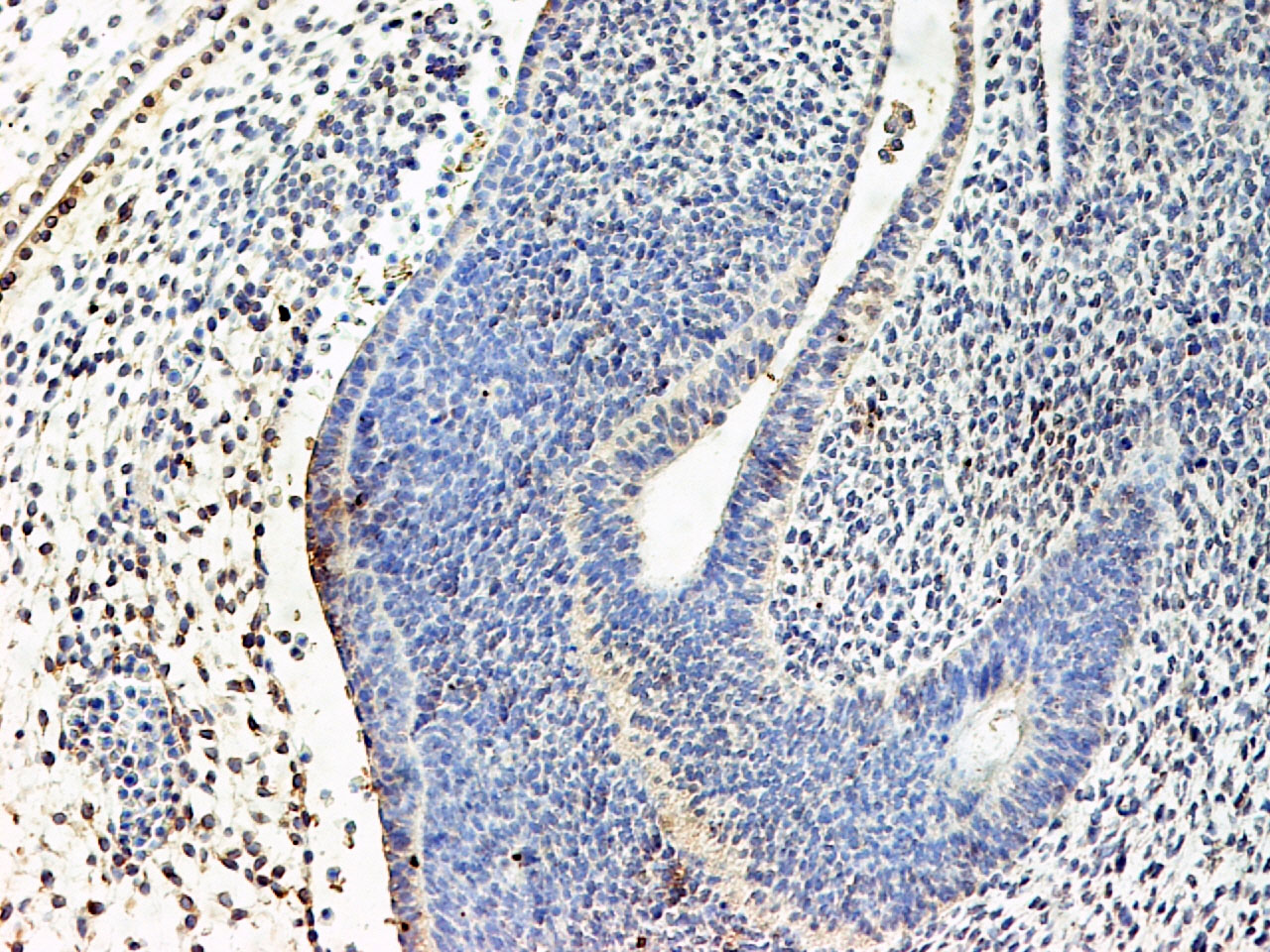

Supplement: Supplemental Information 2 [file peerj-04-1771-s002.zip › 2/c2-38-10 200'╡≈╒√.jpg]

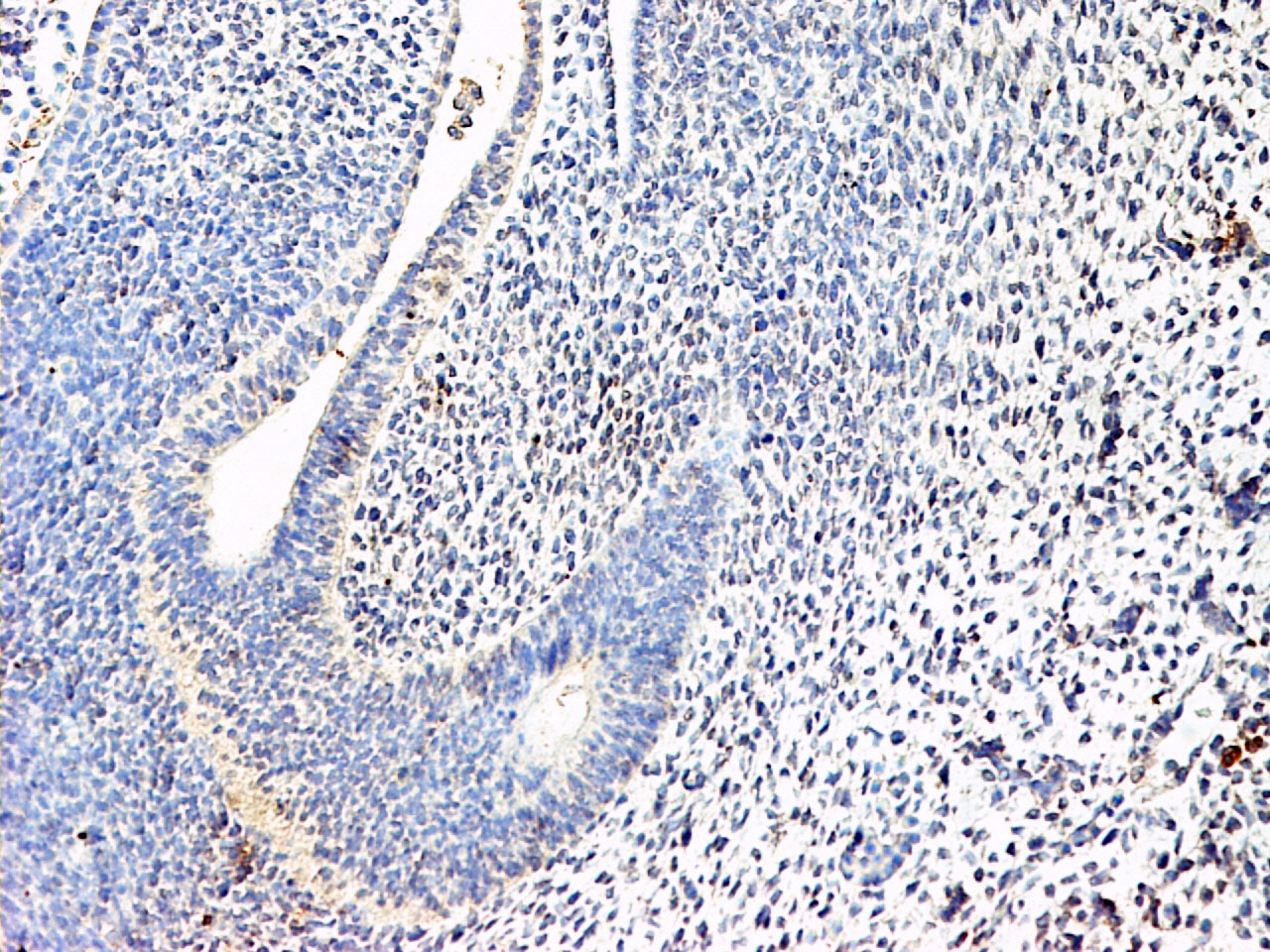

Supplement: Supplemental Information 2 [file peerj-04-1771-s002.zip › 2/c2-38-10 200╡≈╒√.jpg]

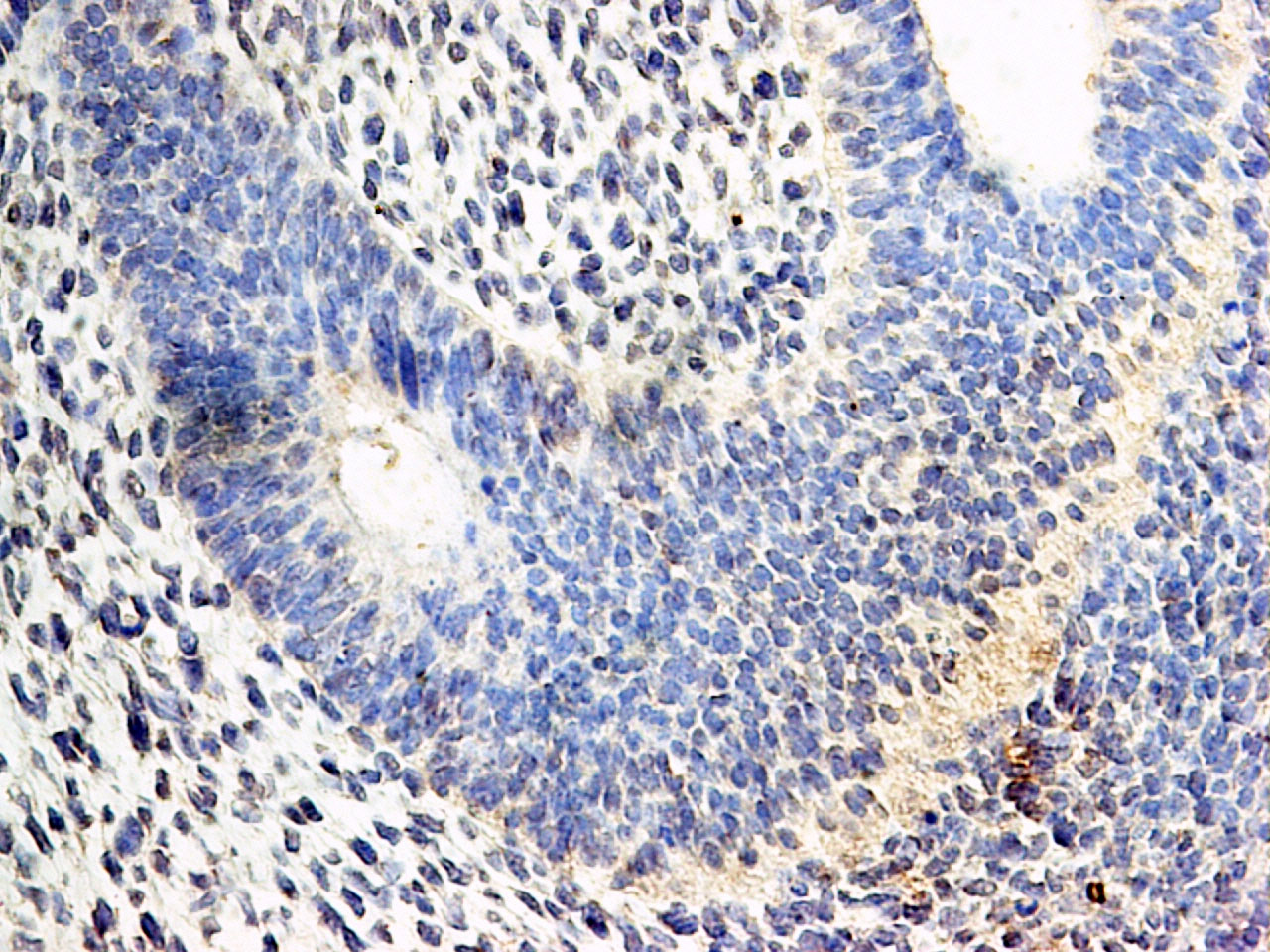

Supplement: Supplemental Information 2 [file peerj-04-1771-s002.zip › 2/c2-38-10 400''╡≈╒√.jpg]

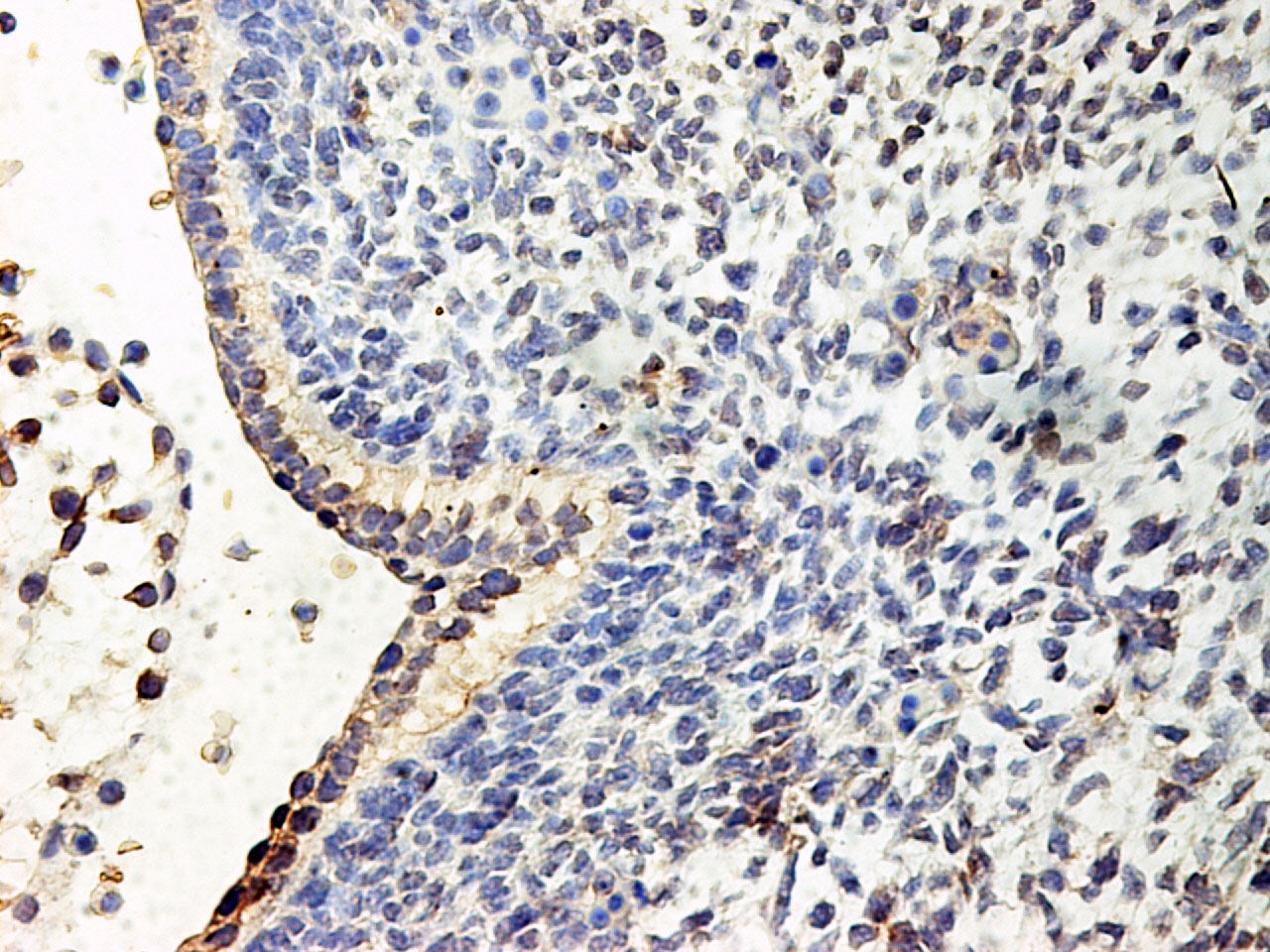

Supplement: Supplemental Information 2 [file peerj-04-1771-s002.zip › 2/c2-38-10 400'╡≈╒√.jpg]

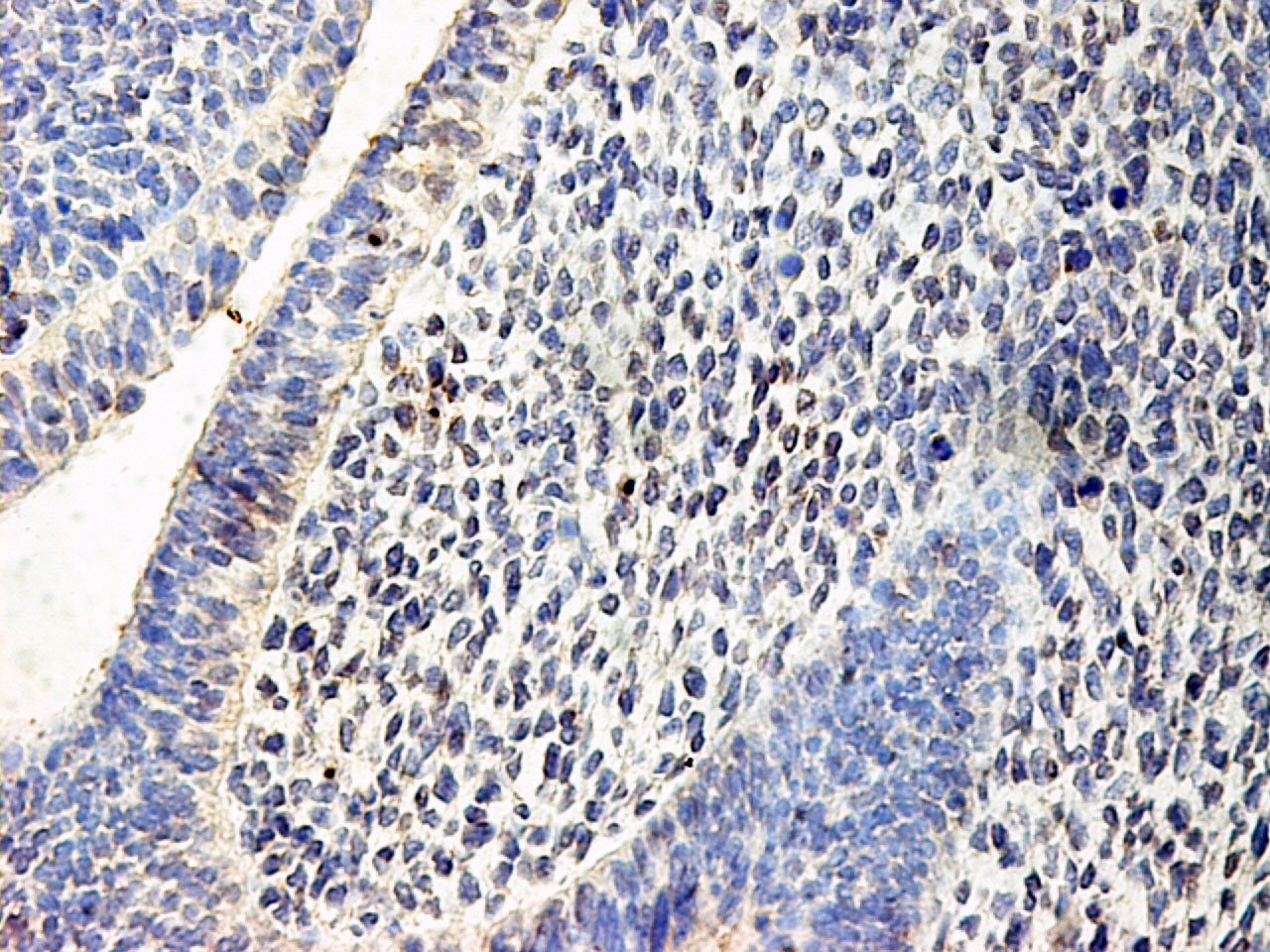

Supplement: Supplemental Information 2 [file peerj-04-1771-s002.zip › 2/c2-38-10 400╡≈╒√.jpg]

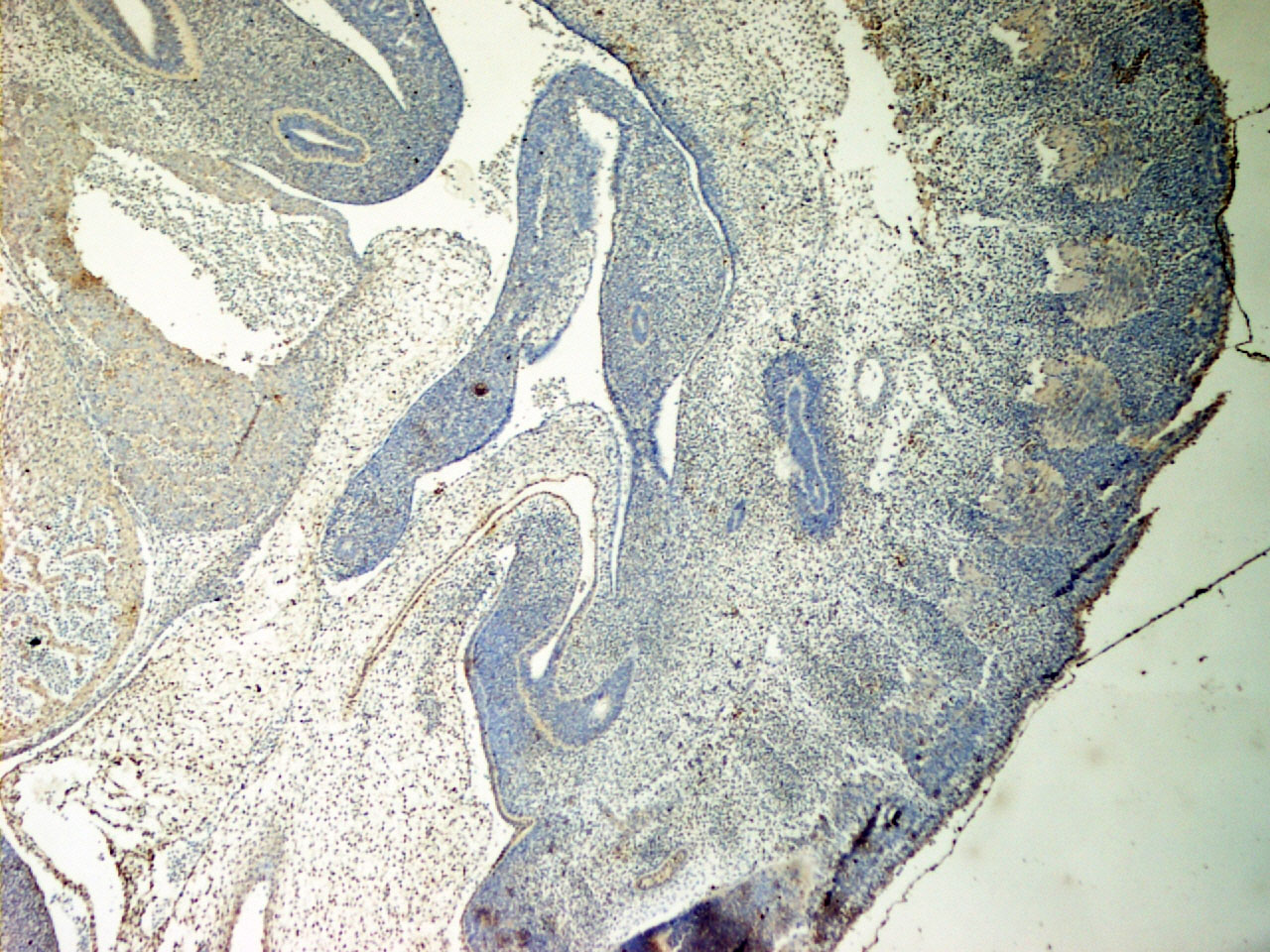

Supplement: Supplemental Information 2 [file peerj-04-1771-s002.zip › 2/c2-38-10 40╡≈╒√.jpg]

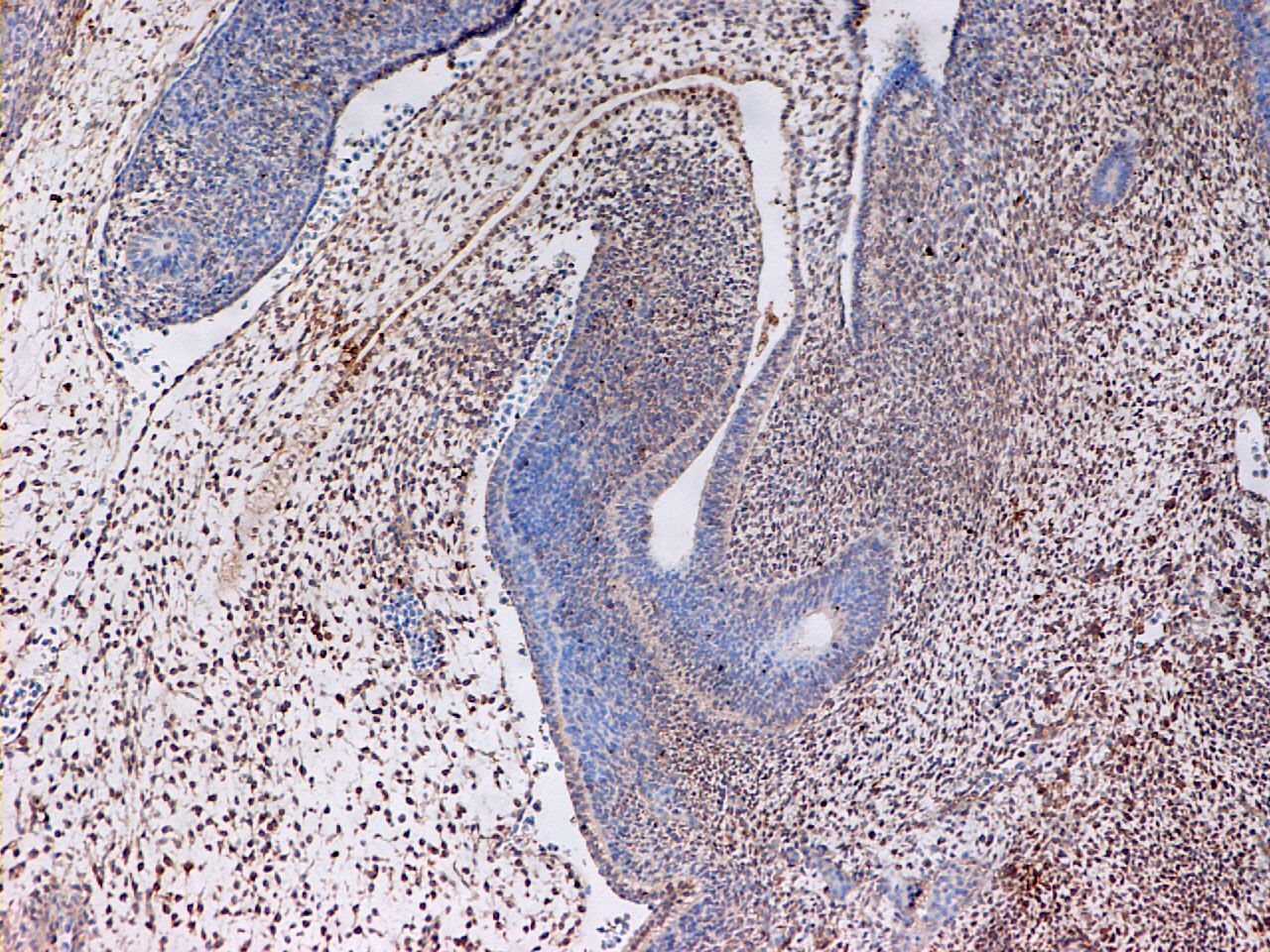

Supplement: Supplemental Information 2 [file peerj-04-1771-s002.zip › 2/C2-38-13 100▒╢╡≈╒√.jpg]

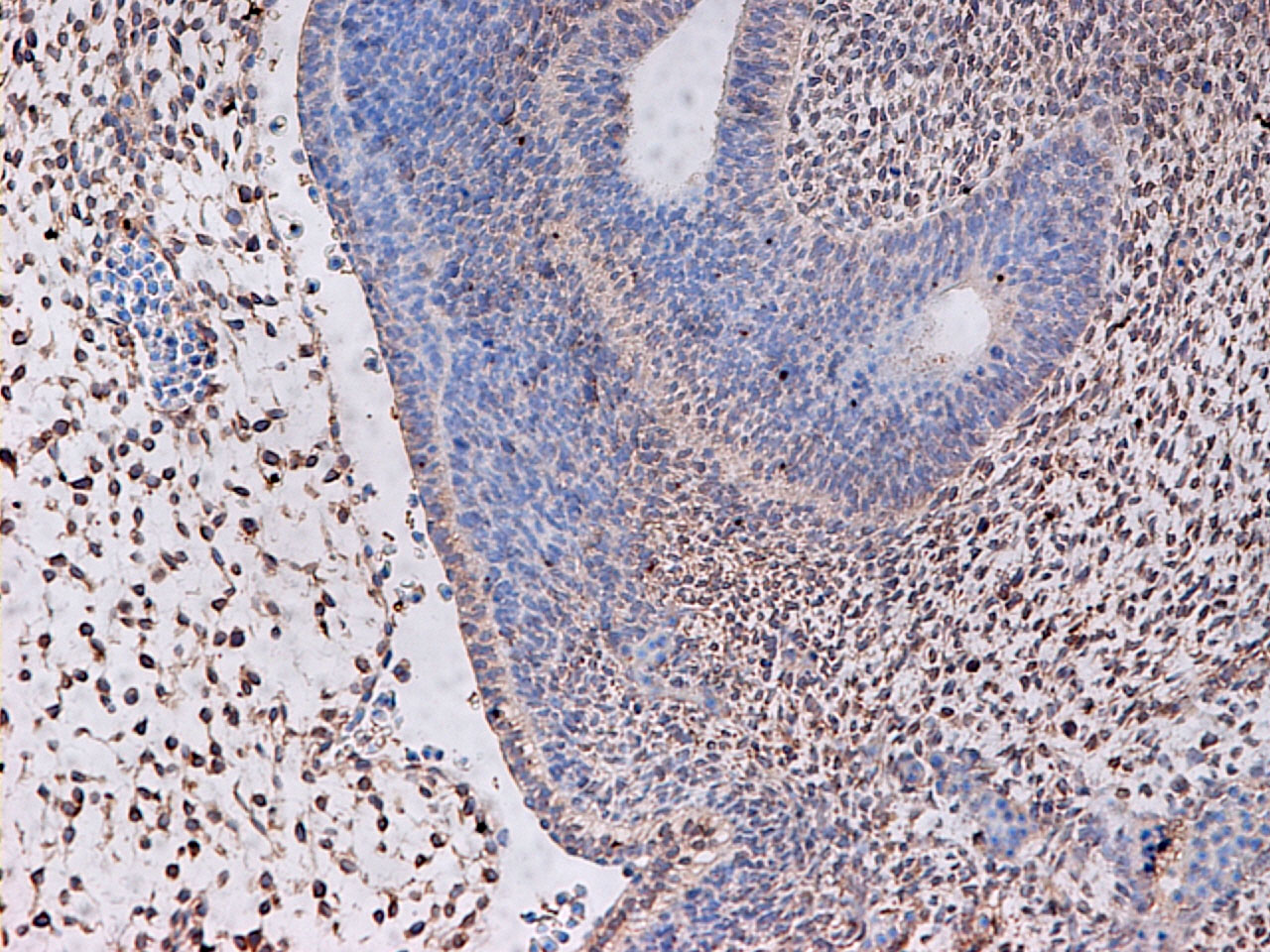

Supplement: Supplemental Information 2 [file peerj-04-1771-s002.zip › 2/C2-38-13 200▒╢'╡≈╒√.jpg]

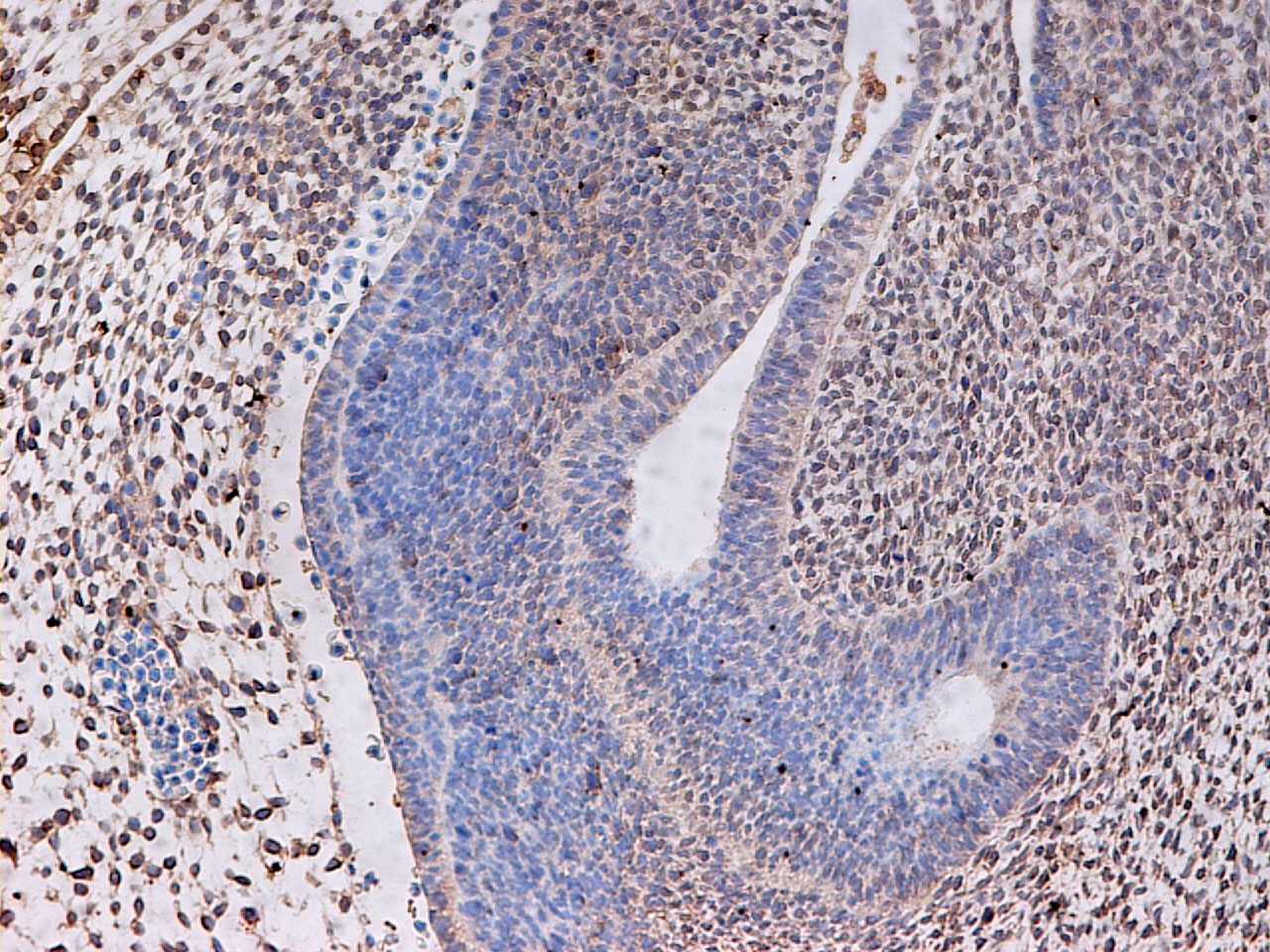

Supplement: Supplemental Information 2 [file peerj-04-1771-s002.zip › 2/C2-38-13 200▒╢╡≈╒√.jpg]

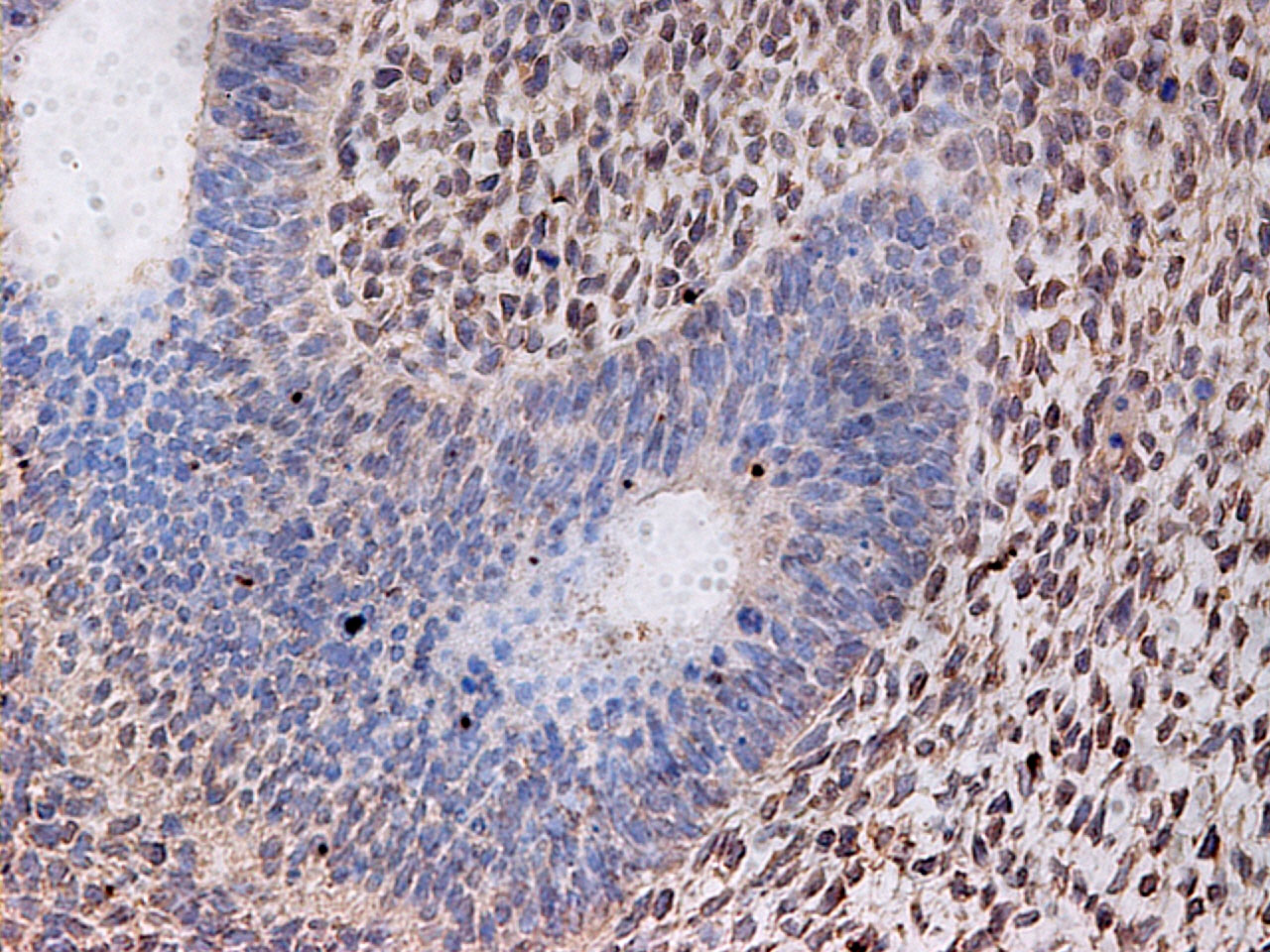

Supplement: Supplemental Information 2 [file peerj-04-1771-s002.zip › 2/C2-38-13 400▒╢'╡≈╒√.jpg]

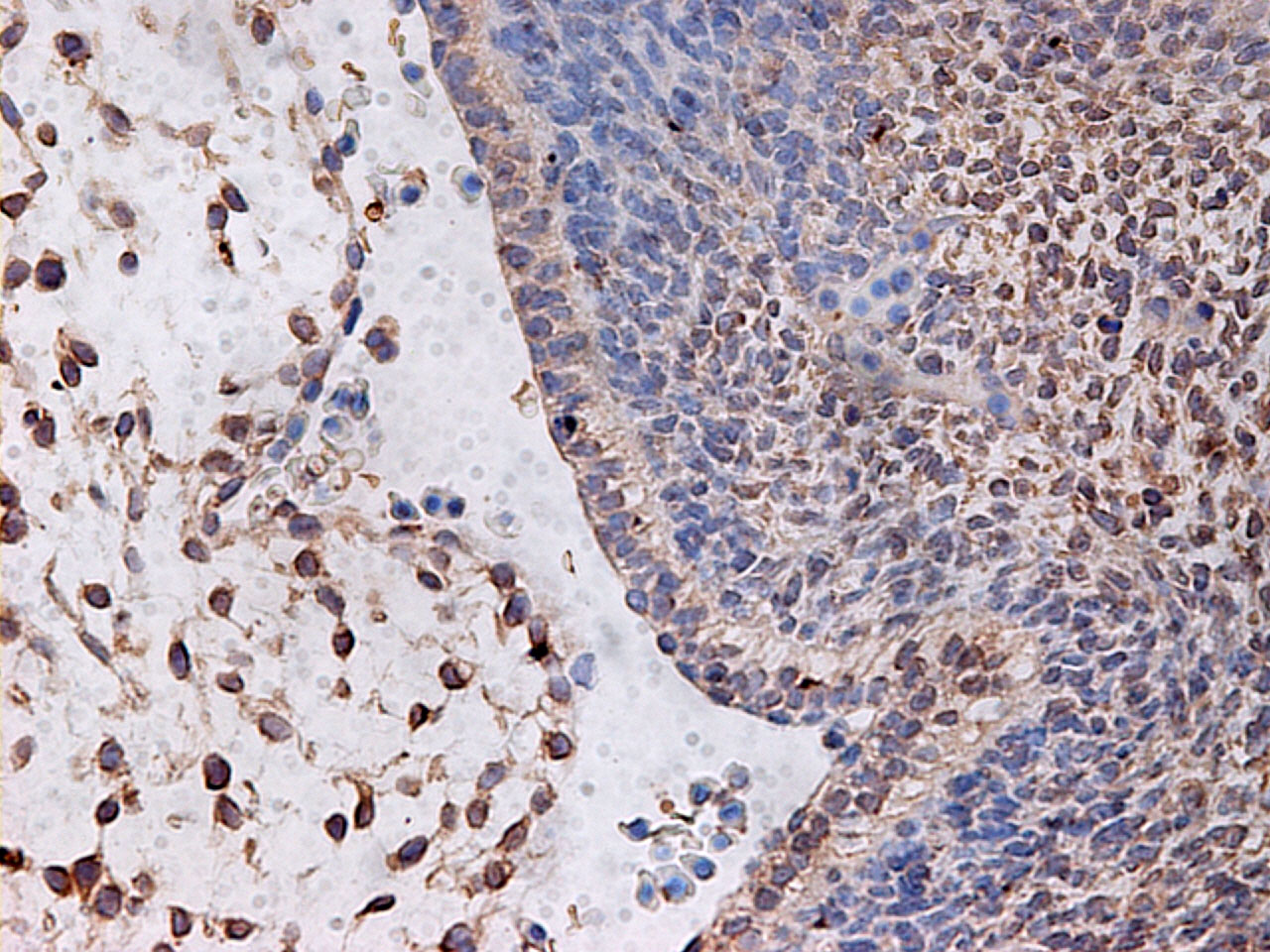

Supplement: Supplemental Information 2 [file peerj-04-1771-s002.zip › 2/C2-38-13 400▒╢╡≈╒√.jpg]

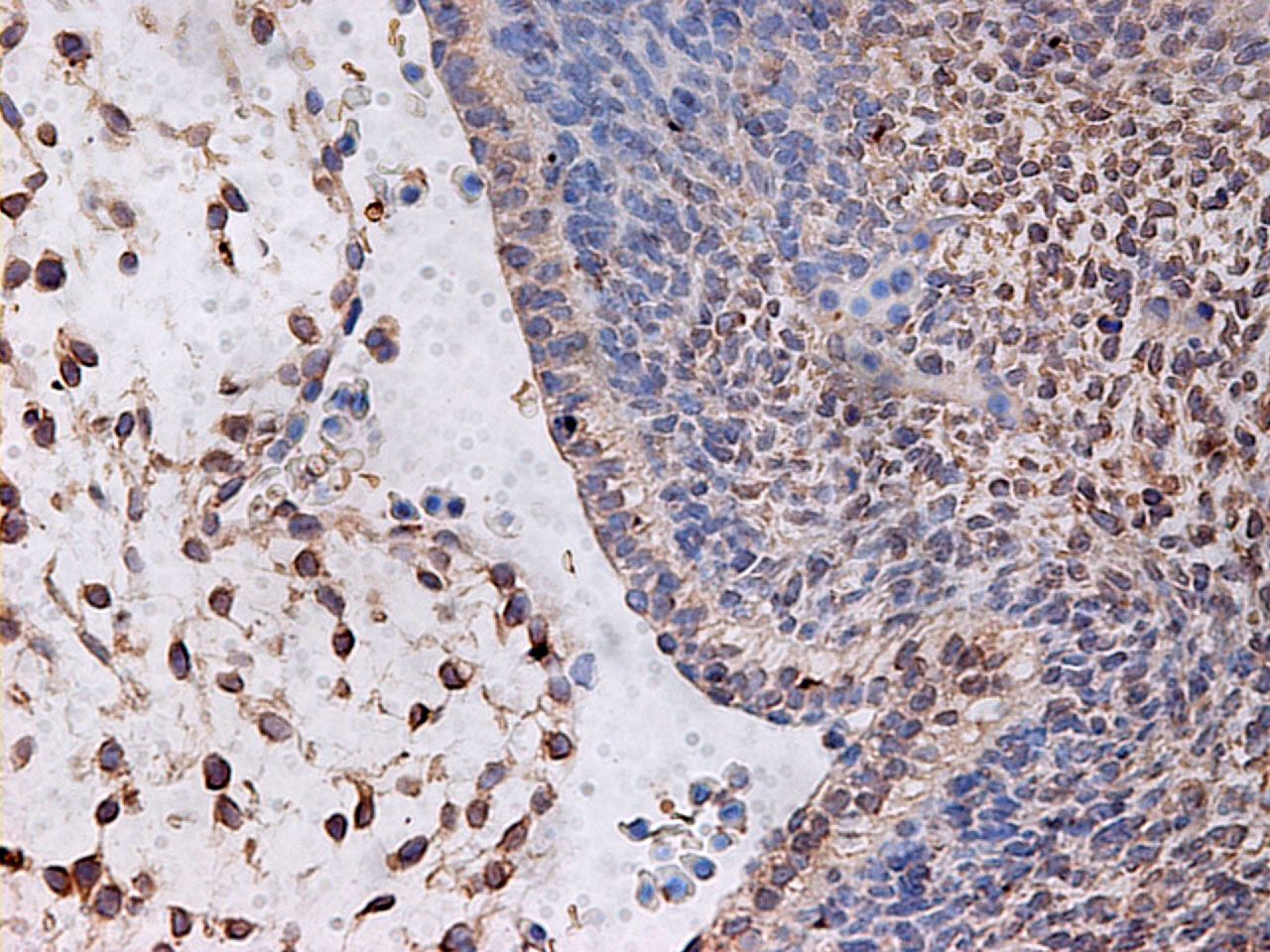

Supplement: Supplemental Information 2 [file peerj-04-1771-s002.zip › 2/C2-38-13 400╡≈╒√.jpg]

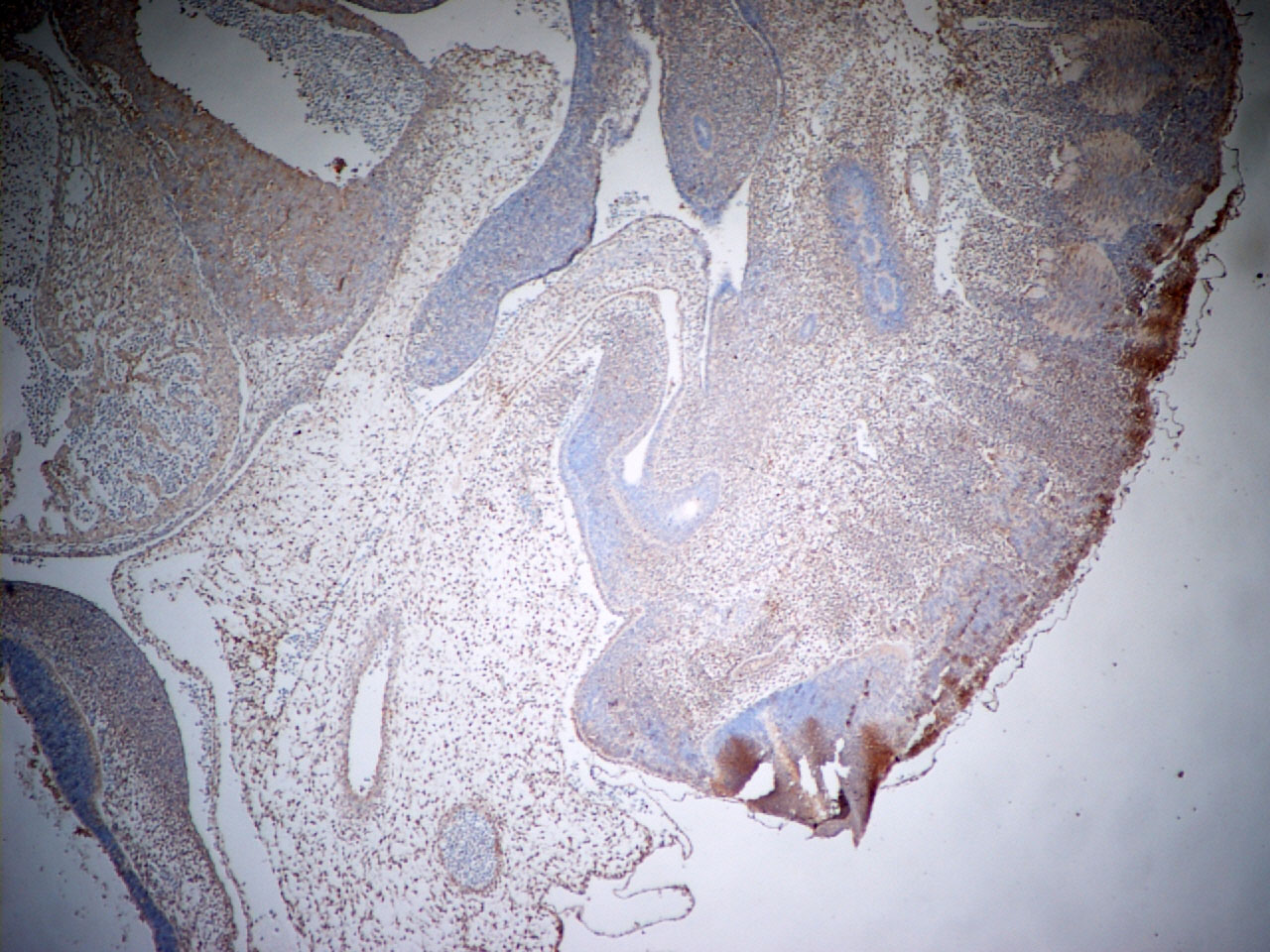

Supplement: Supplemental Information 2 [file peerj-04-1771-s002.zip › 2/C2-38-13 40▒╢╡≈╒√.jpg]

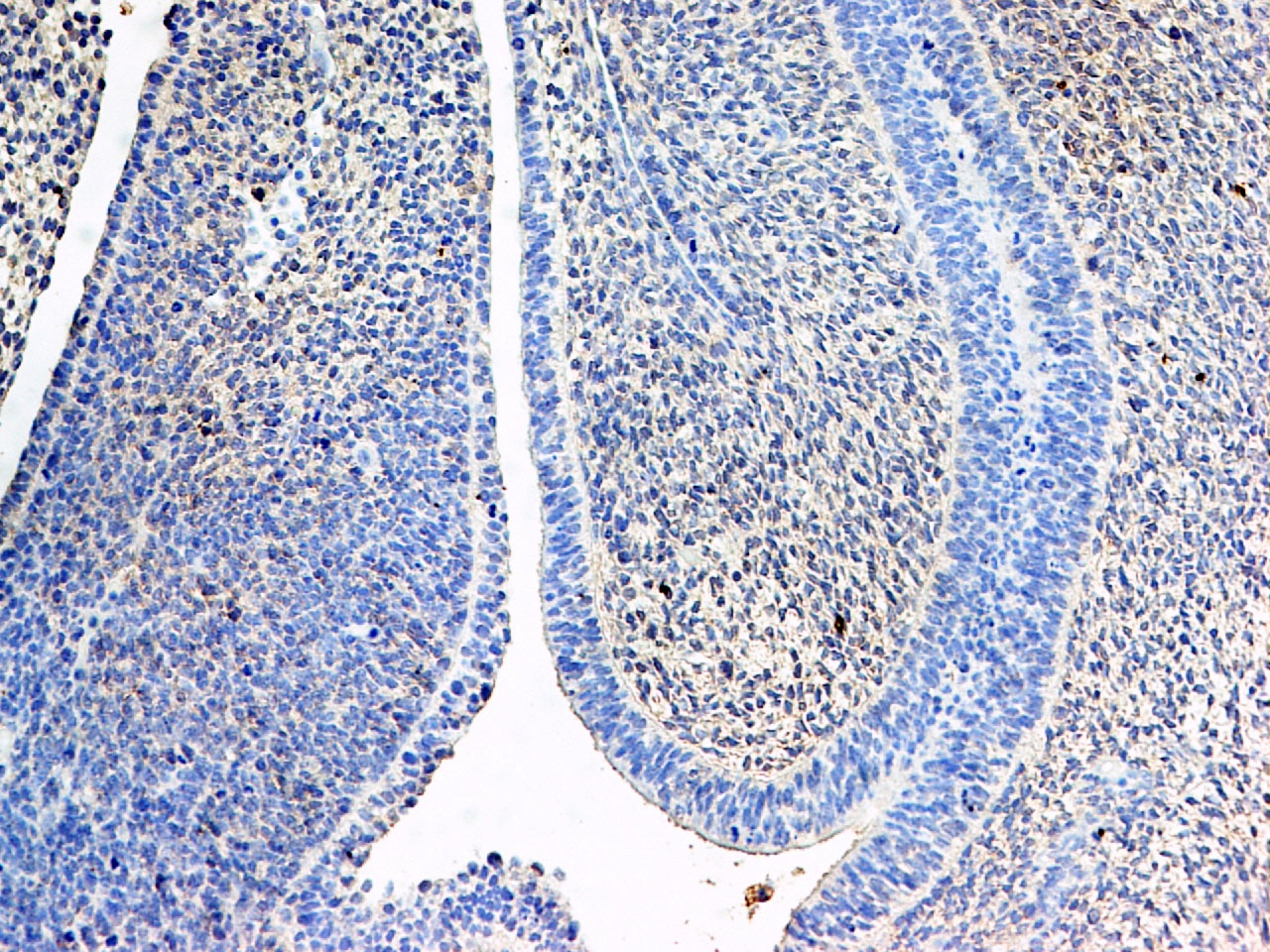

Supplement: Supplemental Information 2 [file peerj-04-1771-s002.zip › 2/C2-7w 200▒╢.jpg]

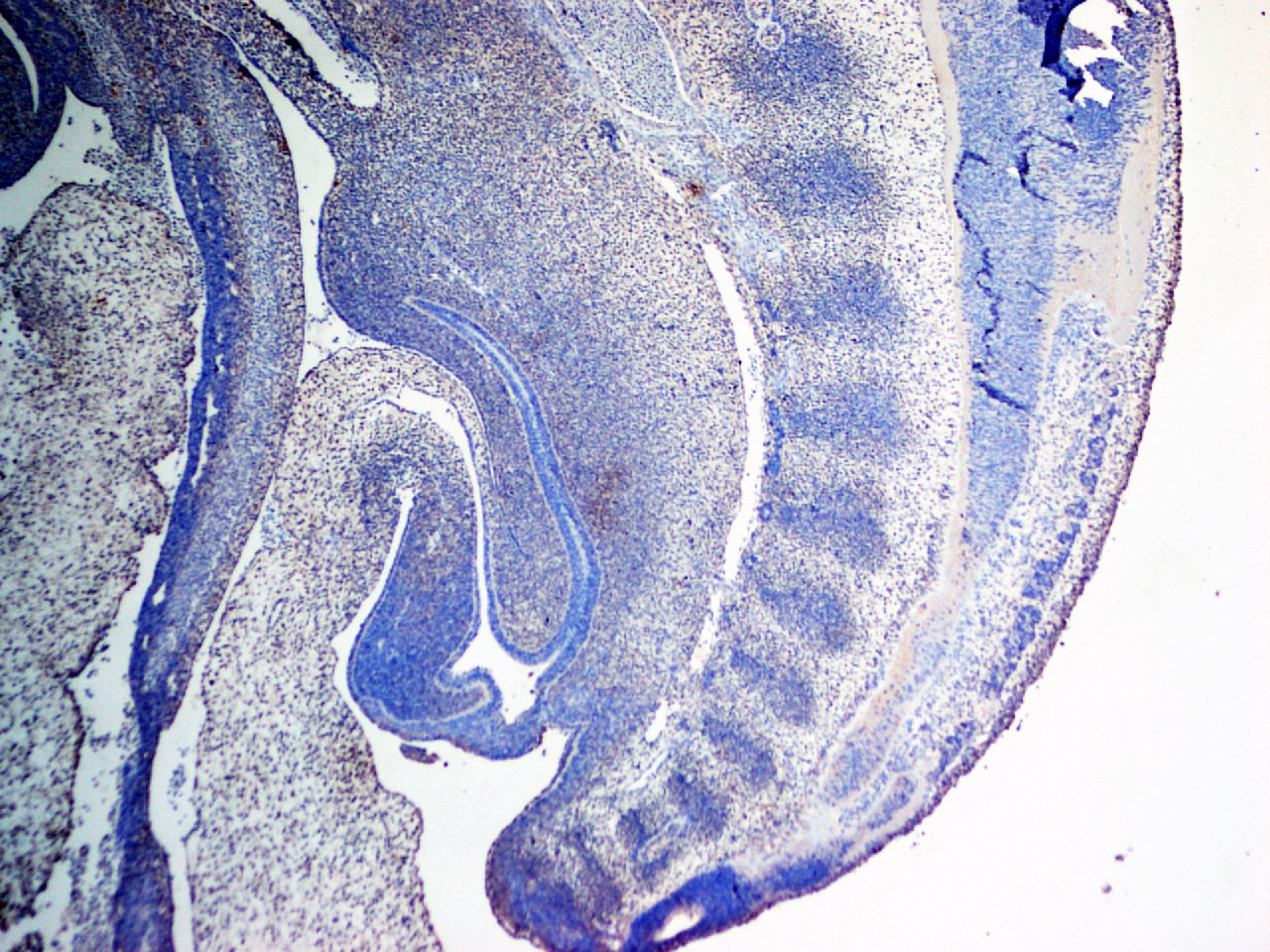

Supplement: Supplemental Information 2 [file peerj-04-1771-s002.zip › 2/C2-7w 40▒╢.jpg]

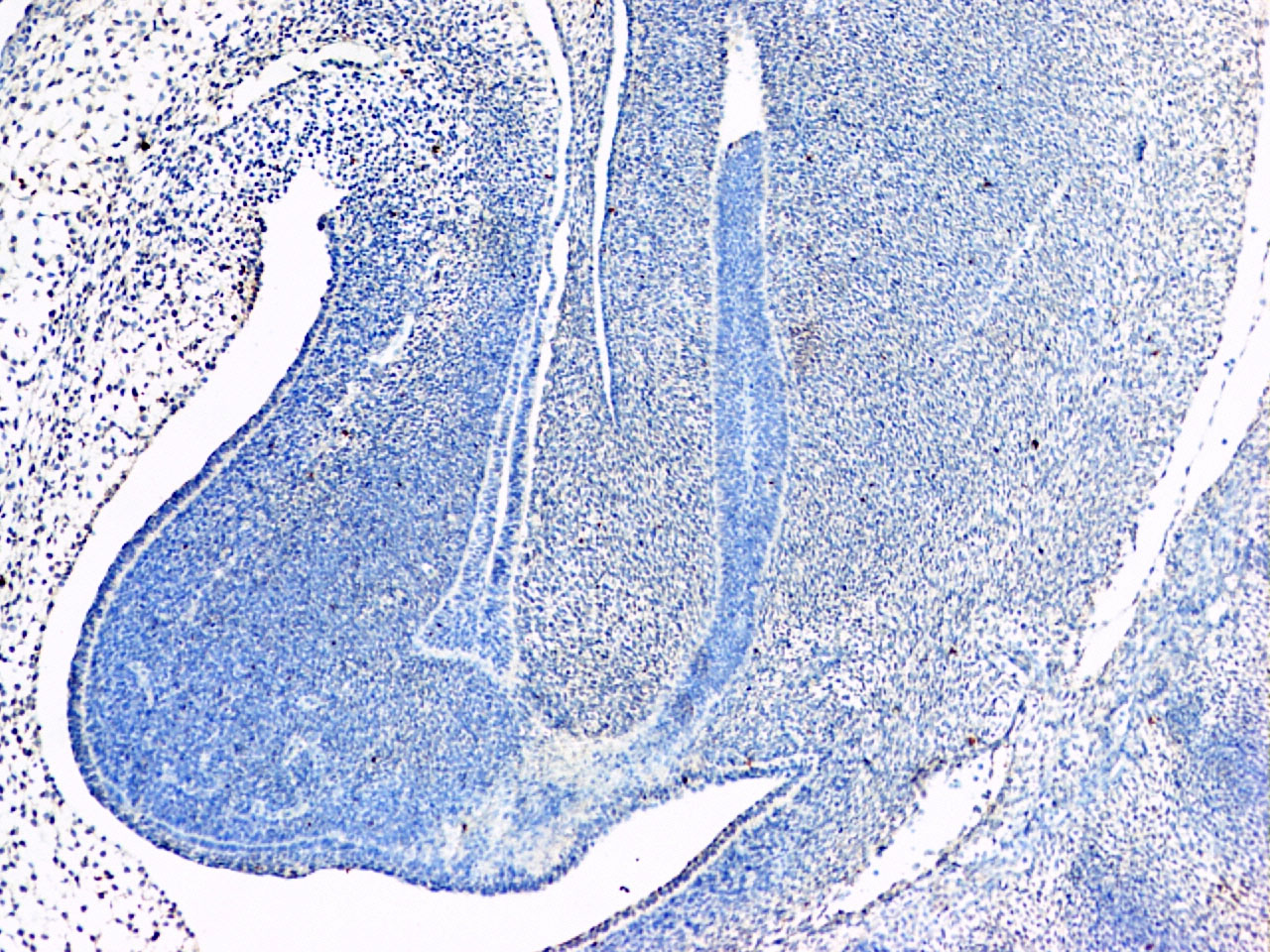

Supplement: Supplemental Information 2 [file peerj-04-1771-s002.zip › 2/c2-8-4 100╡≈╒√.jpg]

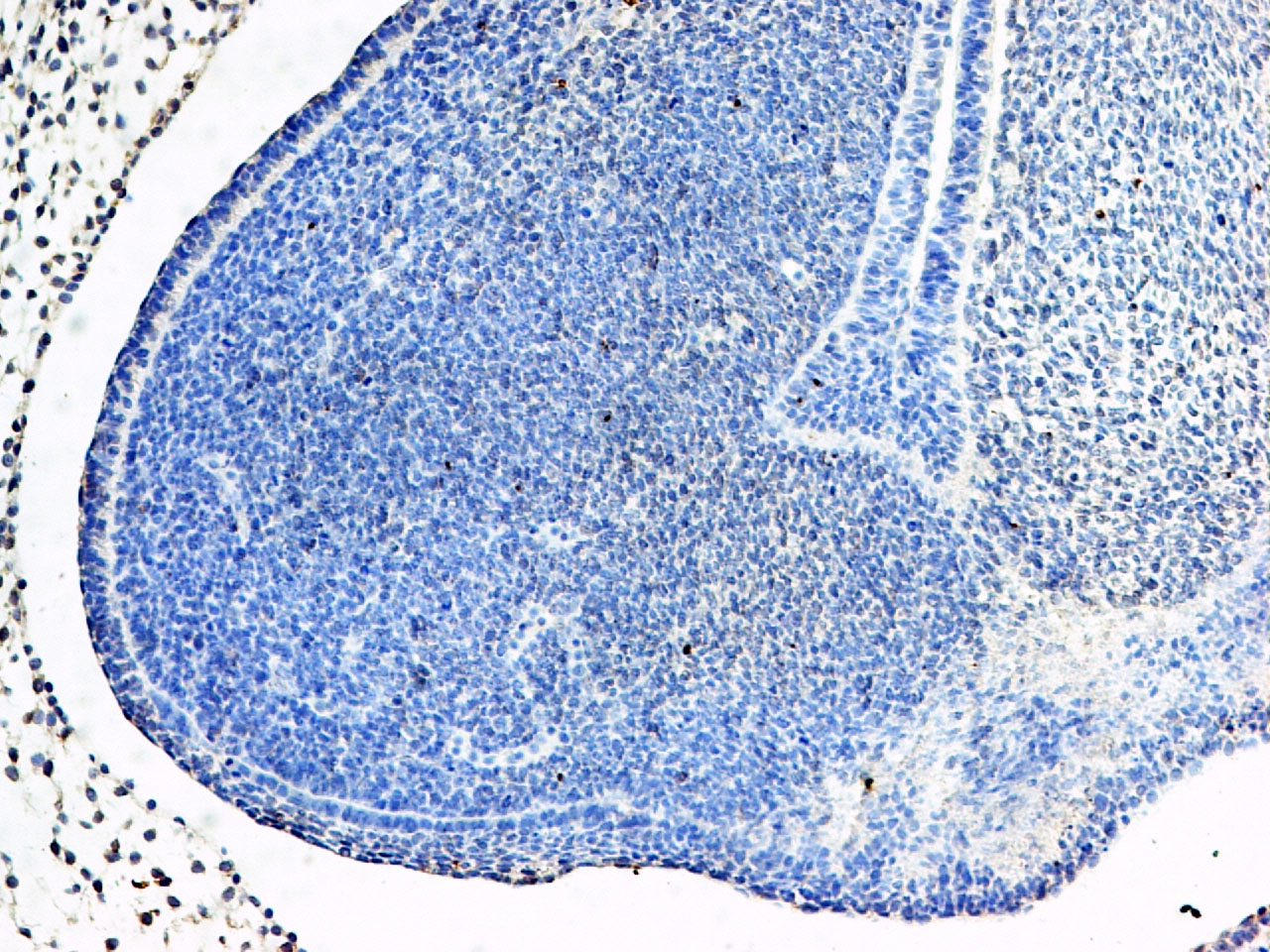

Supplement: Supplemental Information 2 [file peerj-04-1771-s002.zip › 2/c2-8-4 200''╡≈╒√.jpg]

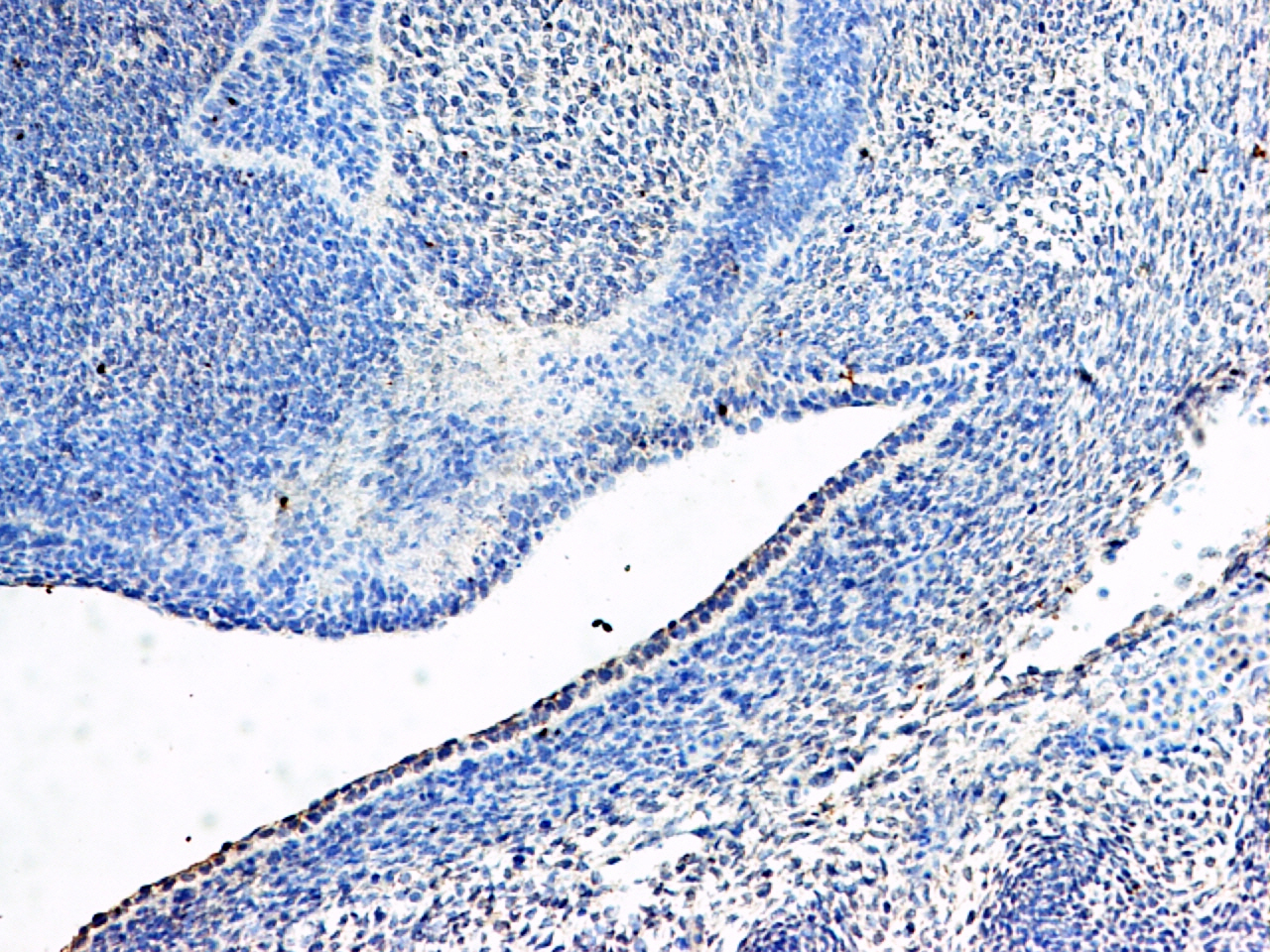

Supplement: Supplemental Information 2 [file peerj-04-1771-s002.zip › 2/c2-8-4 200'╡≈╒√.jpg]

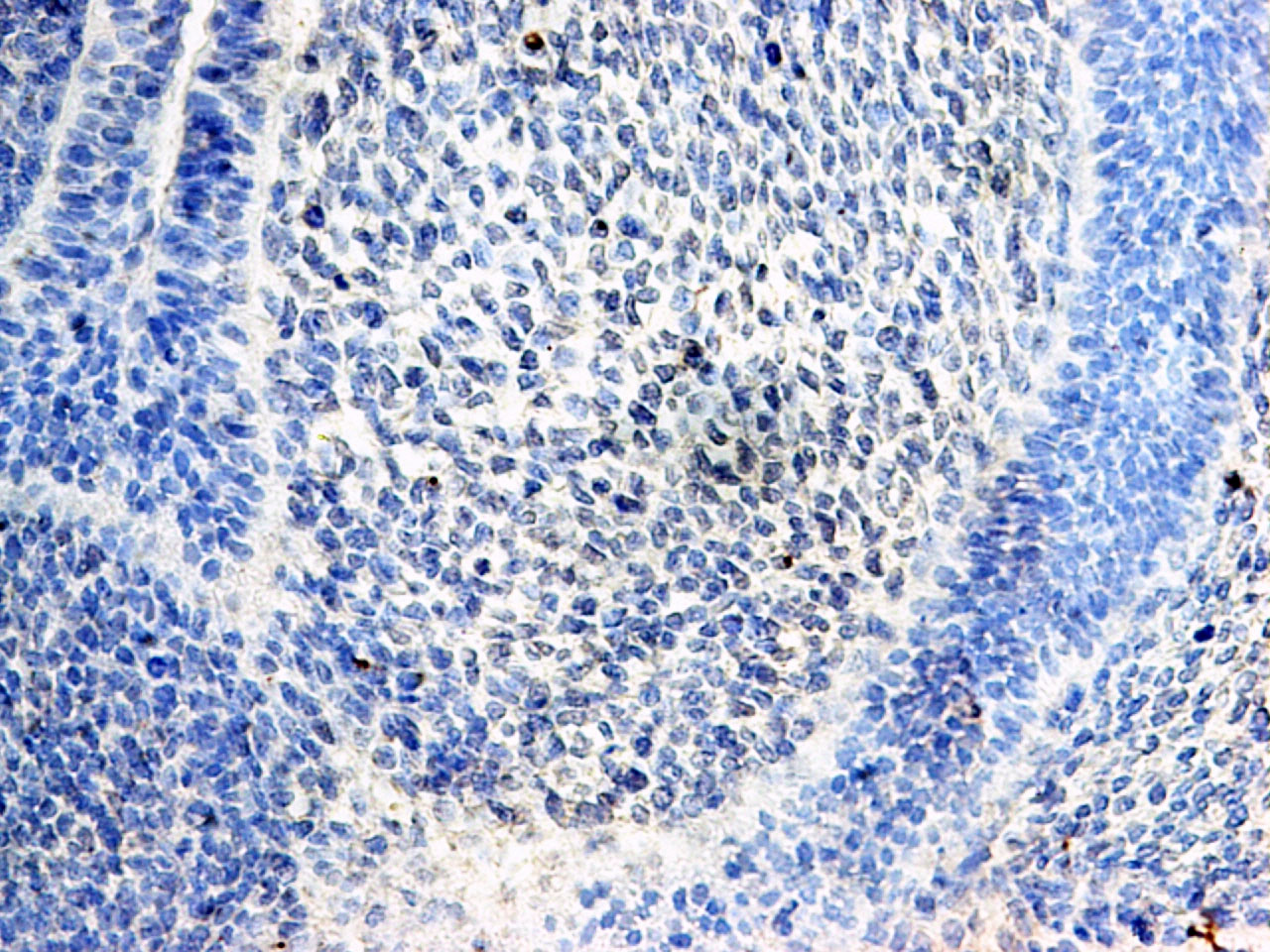

Supplement: Supplemental Information 2 [file peerj-04-1771-s002.zip › 2/c2-8-4 400'''╡≈╒√.jpg]

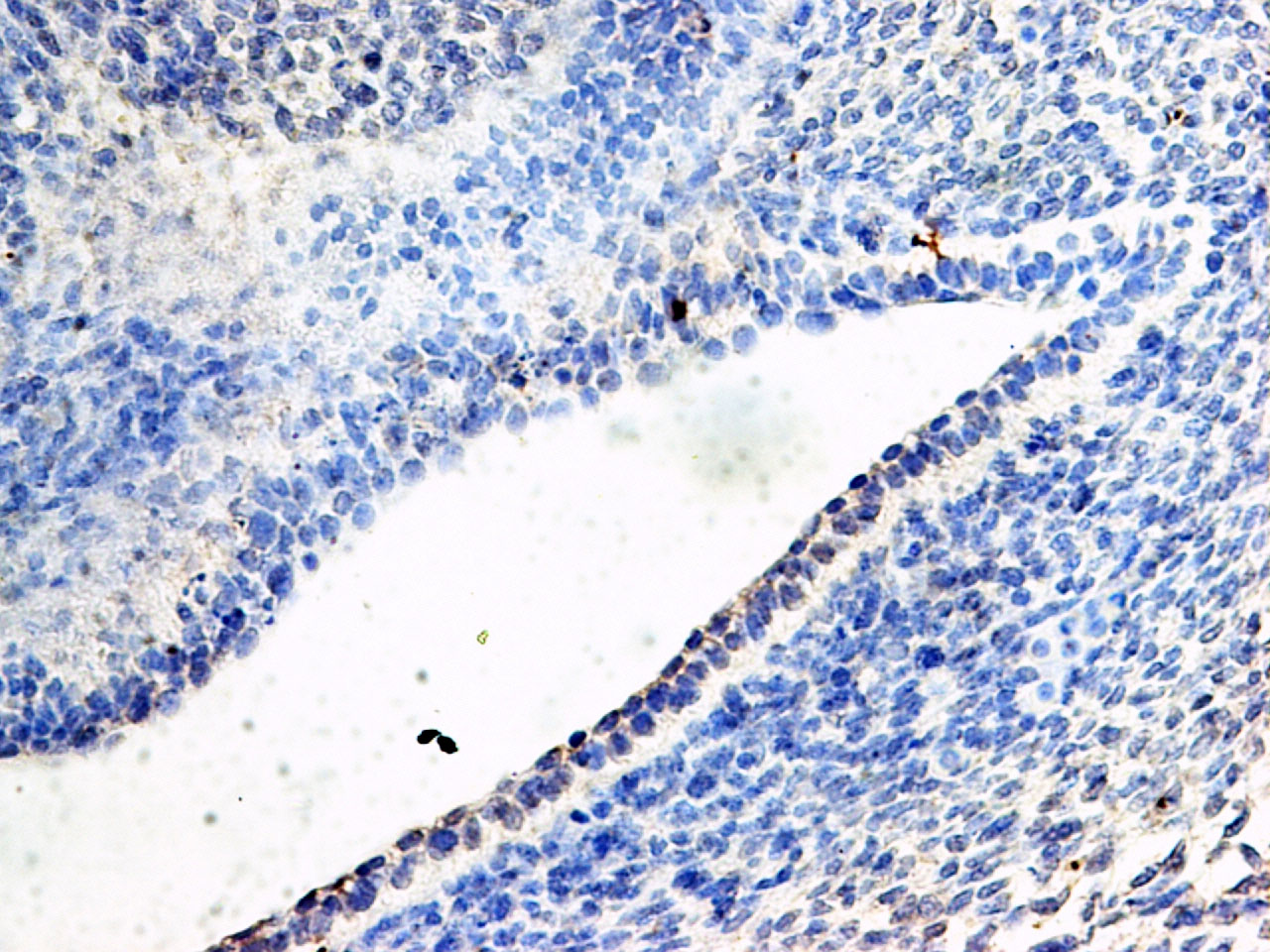

Supplement: Supplemental Information 2 [file peerj-04-1771-s002.zip › 2/c2-8-4 400''╡≈╒√.jpg]

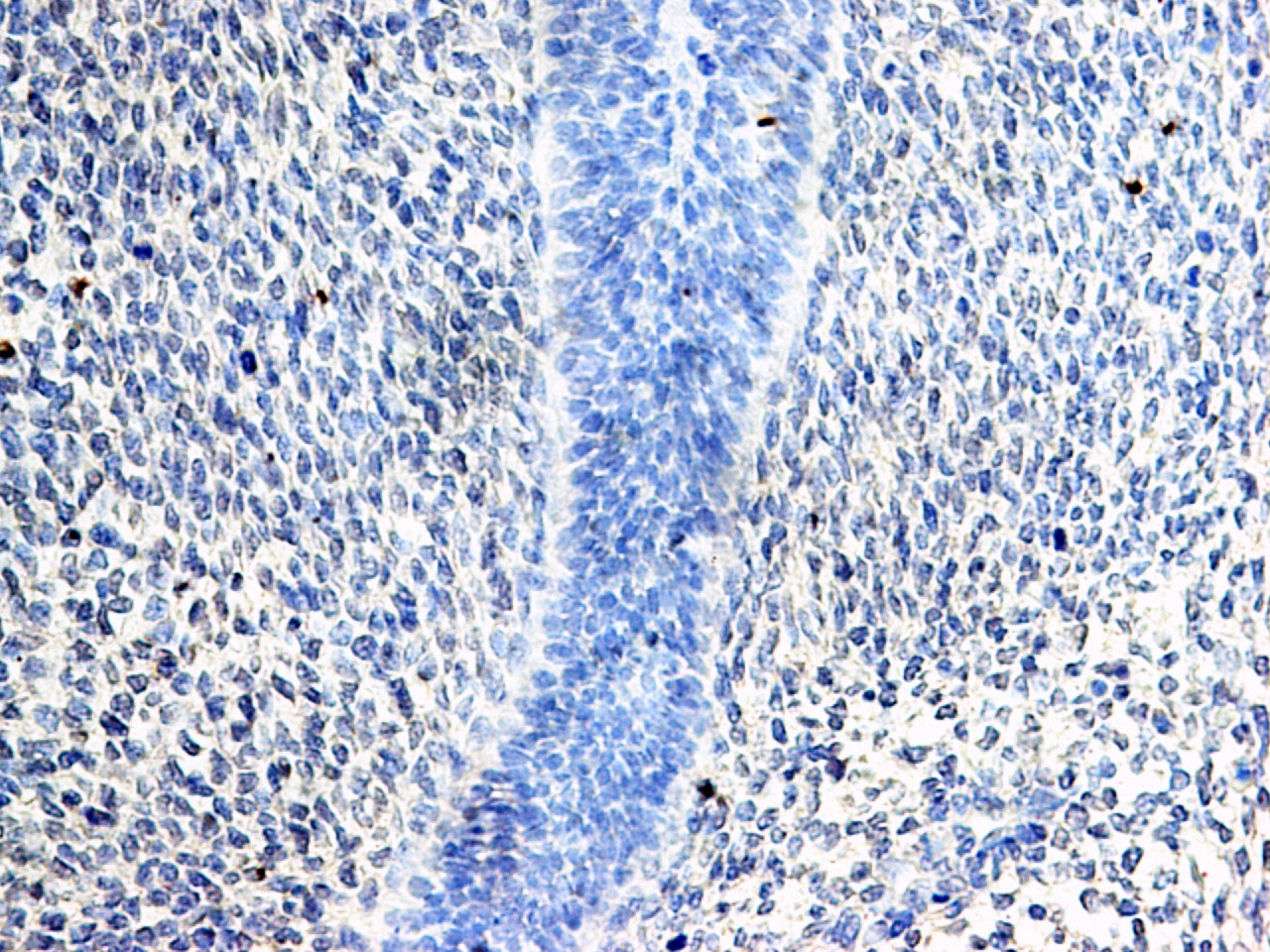

Supplement: Supplemental Information 2 [file peerj-04-1771-s002.zip › 2/c2-8-4 400'╡≈╒√.jpg]

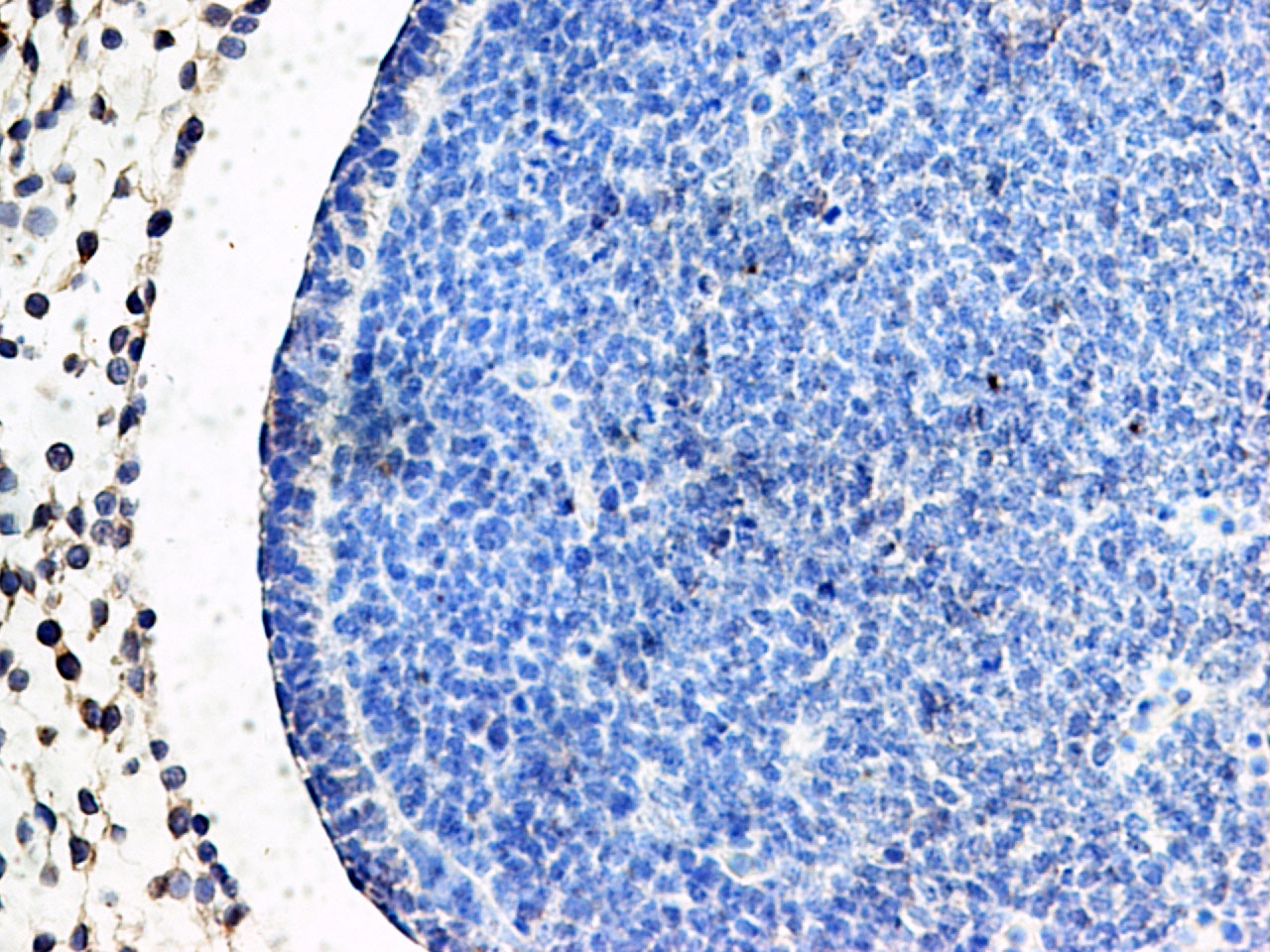

Supplement: Supplemental Information 2 [file peerj-04-1771-s002.zip › 2/c2-8-4 400╡≈╒√.jpg]

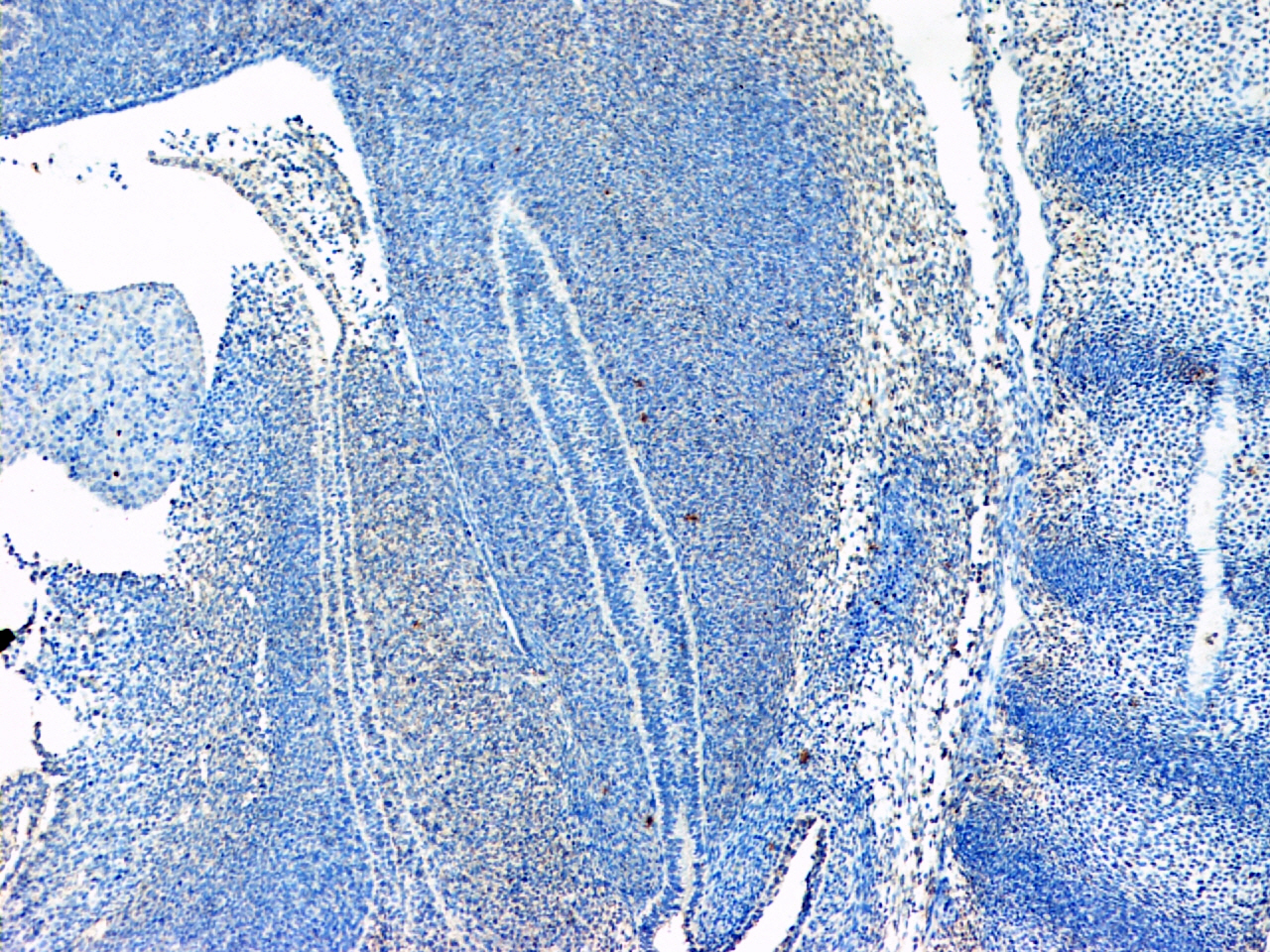

Supplement: Supplemental Information 3 [file peerj-04-1771-s003.zip › 4/c4-1-7 100╡≈╒√.jpg]

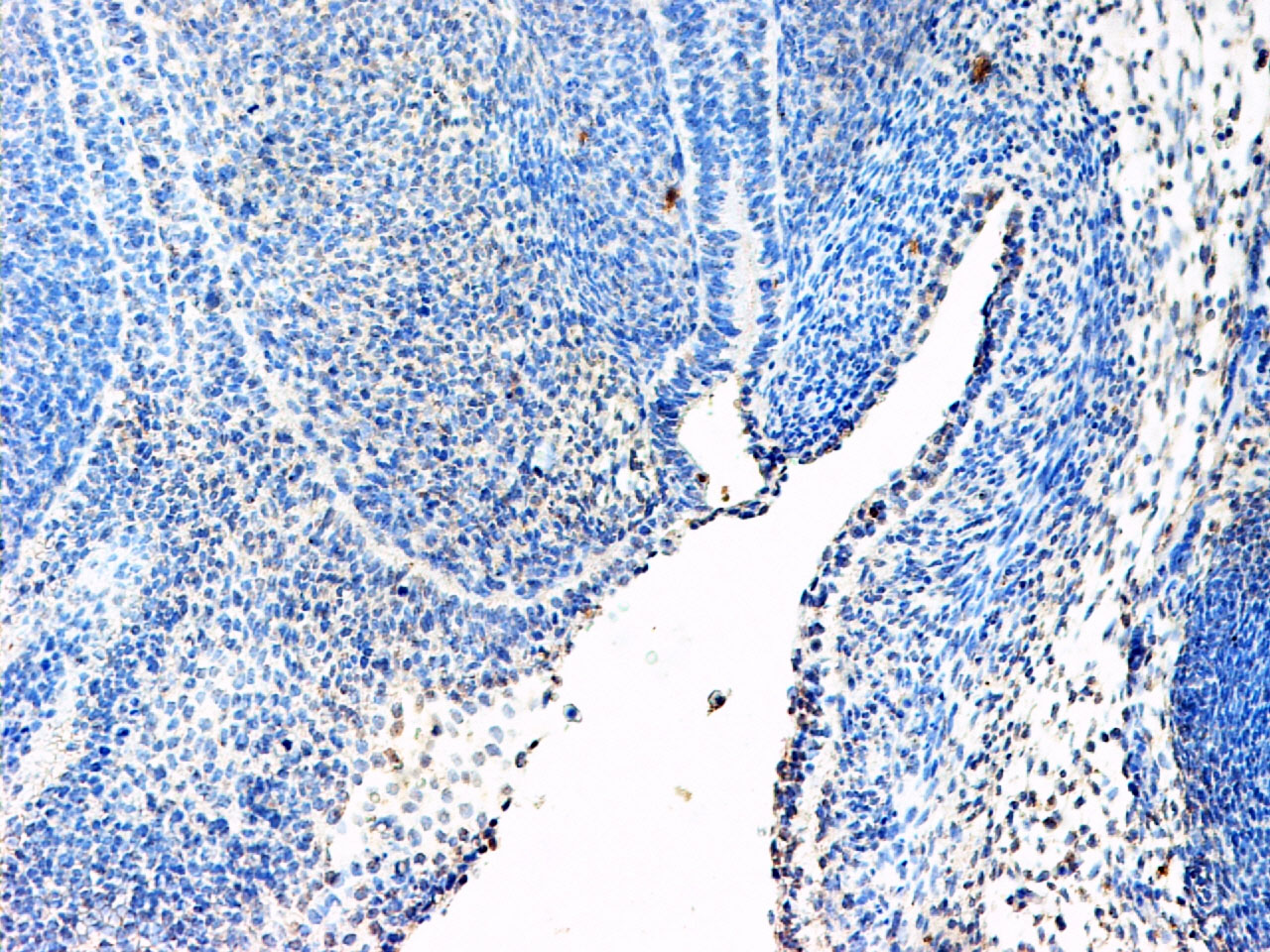

Supplement: Supplemental Information 3 [file peerj-04-1771-s003.zip › 4/c4-1-7 200í»╡≈╒√.jpg]

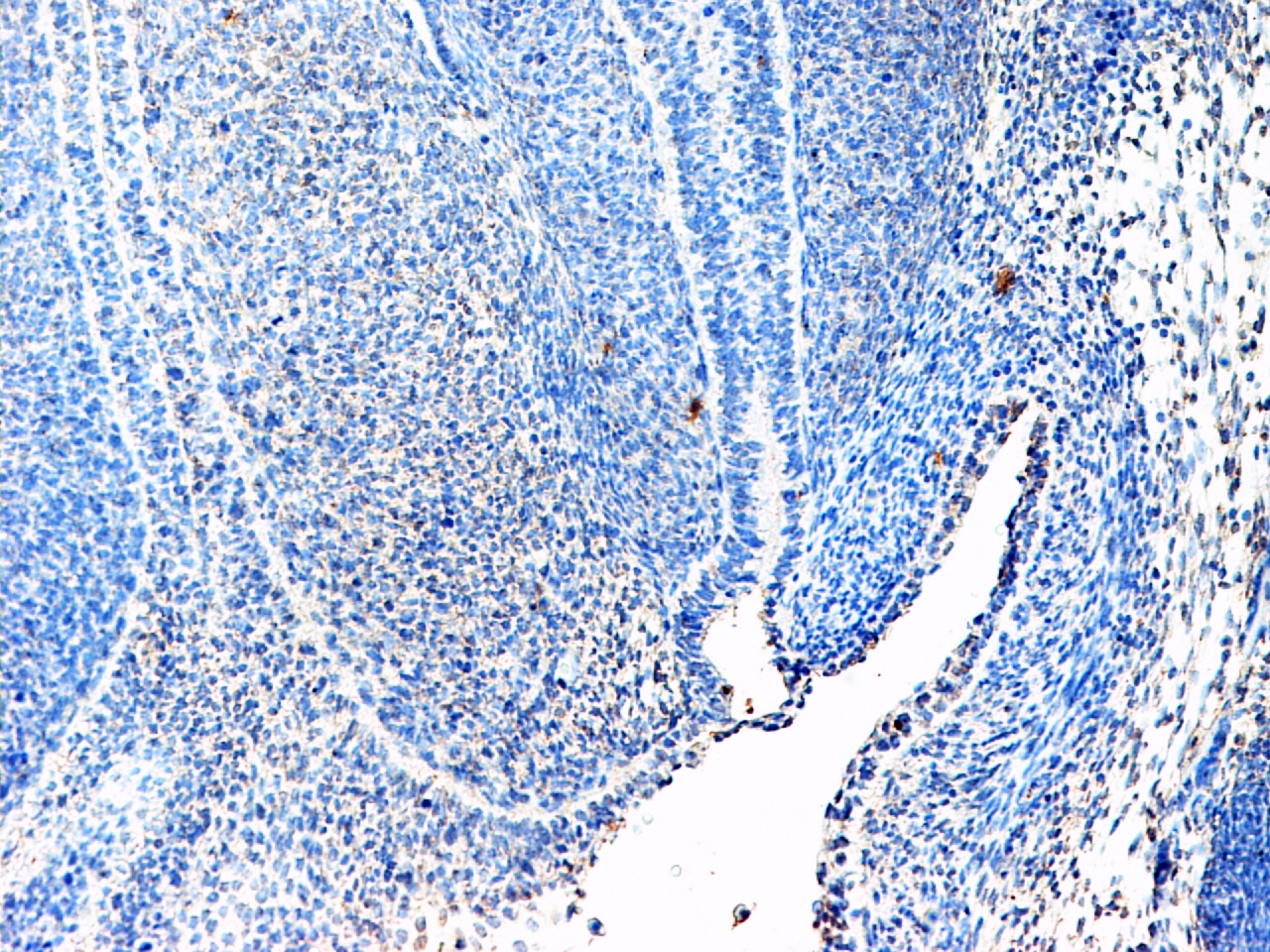

Supplement: Supplemental Information 3 [file peerj-04-1771-s003.zip › 4/c4-1-7 200╡≈╒√.jpg]

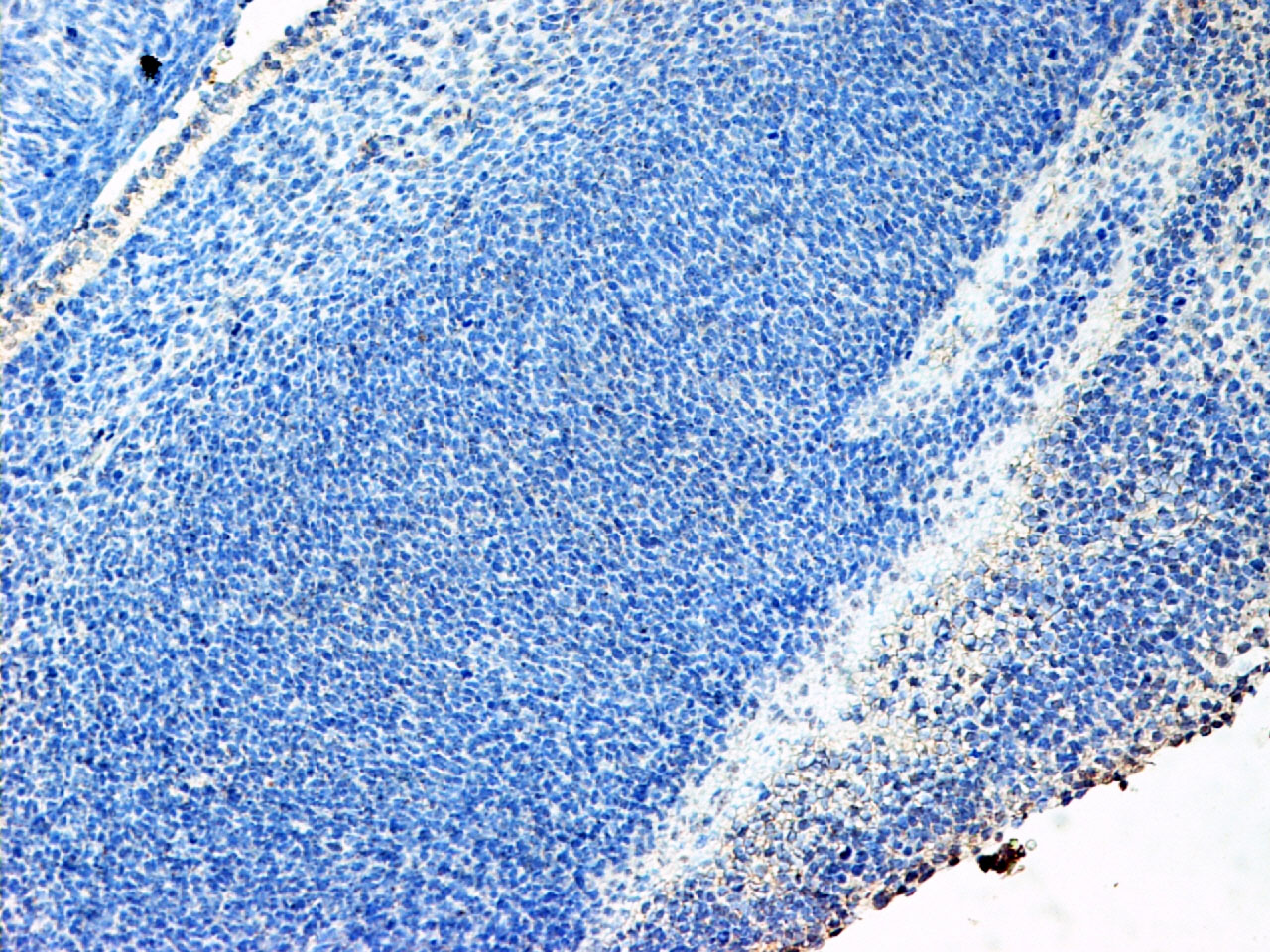

Supplement: Supplemental Information 3 [file peerj-04-1771-s003.zip › 4/c4-1-7 400í«╡≈╒√.jpg]

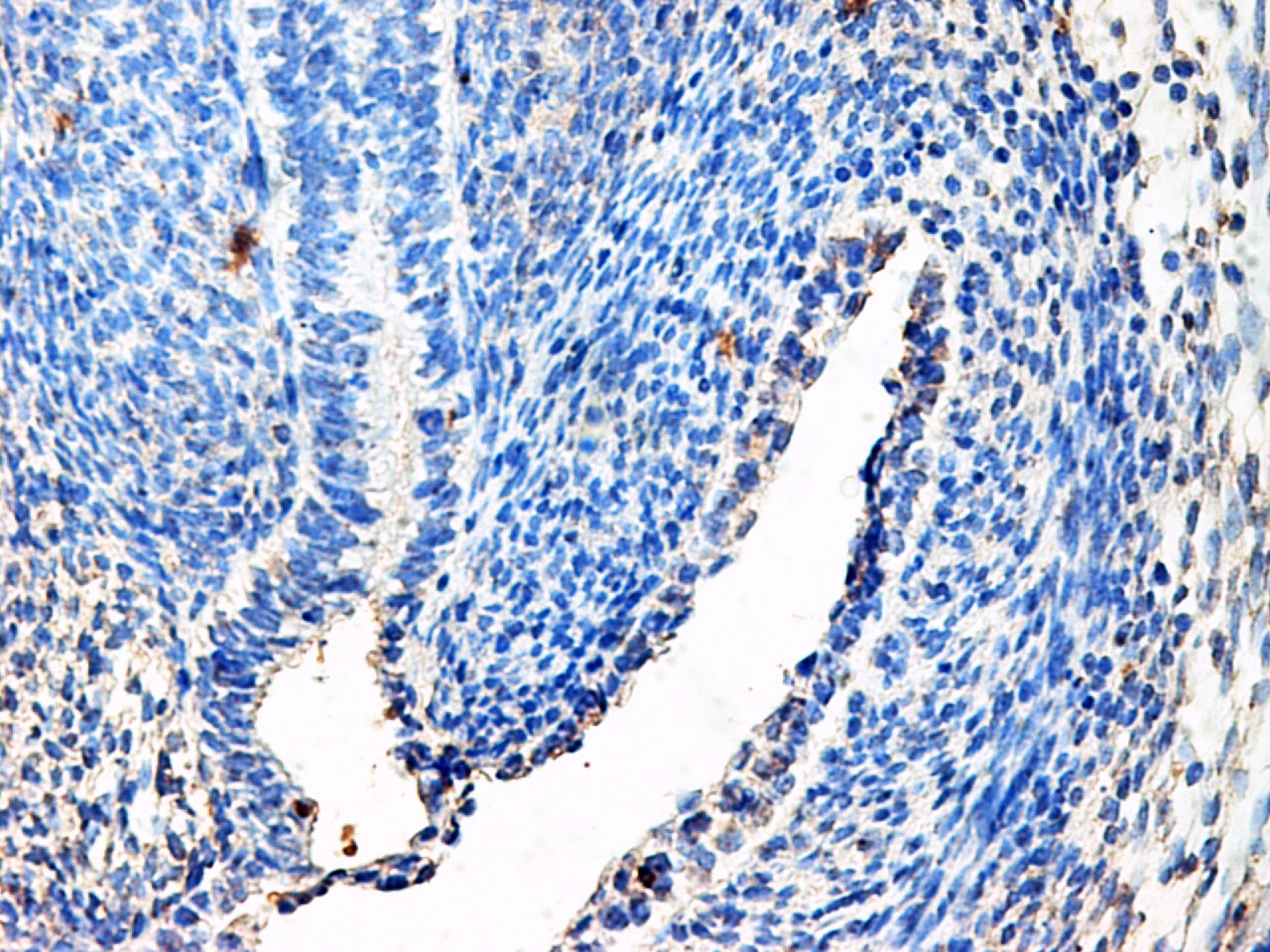

Supplement: Supplemental Information 3 [file peerj-04-1771-s003.zip › 4/c4-1-7 400╡≈╒√.jpg]

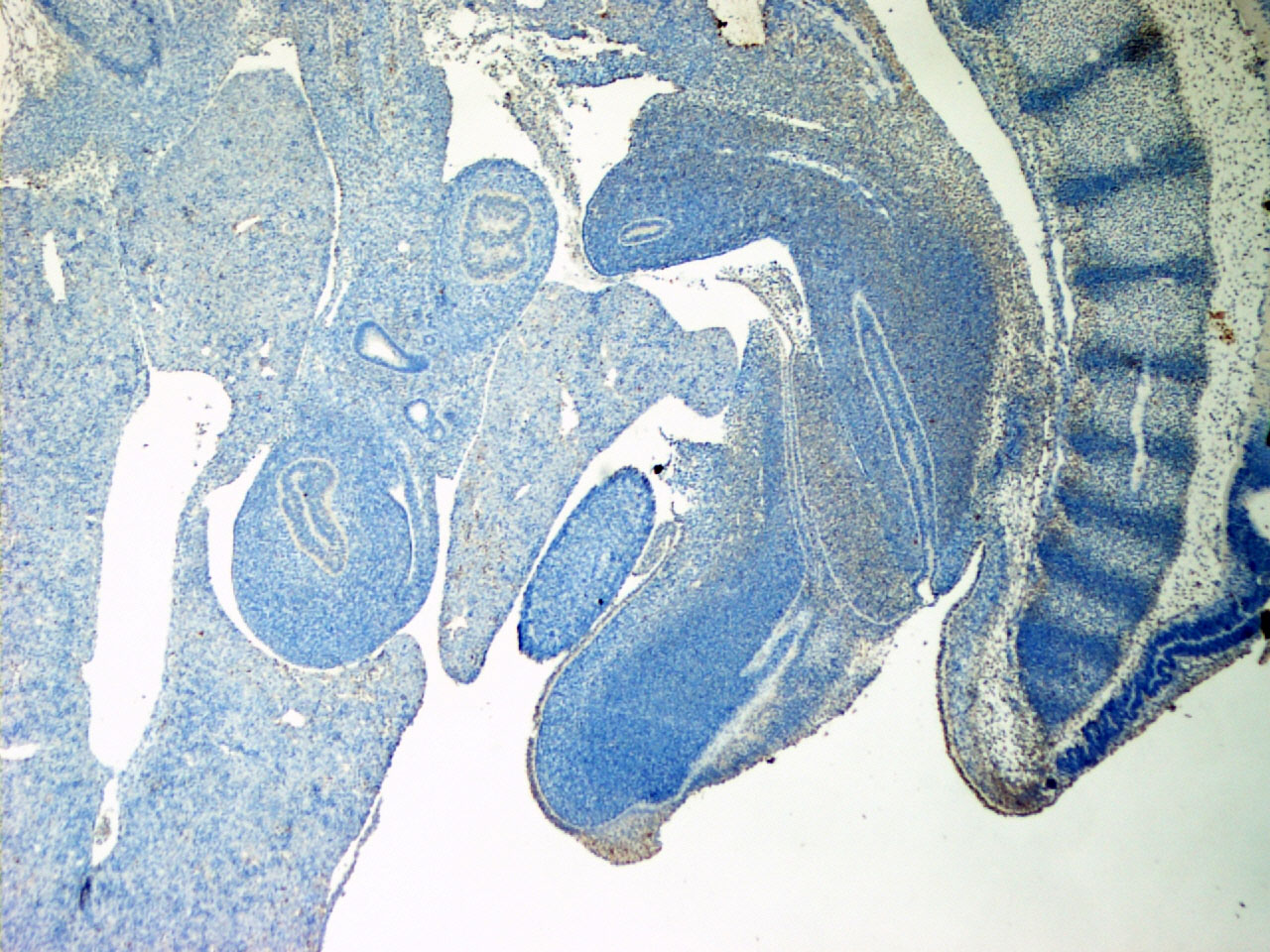

Supplement: Supplemental Information 3 [file peerj-04-1771-s003.zip › 4/c4-1-7 40╡≈╒√.jpg]

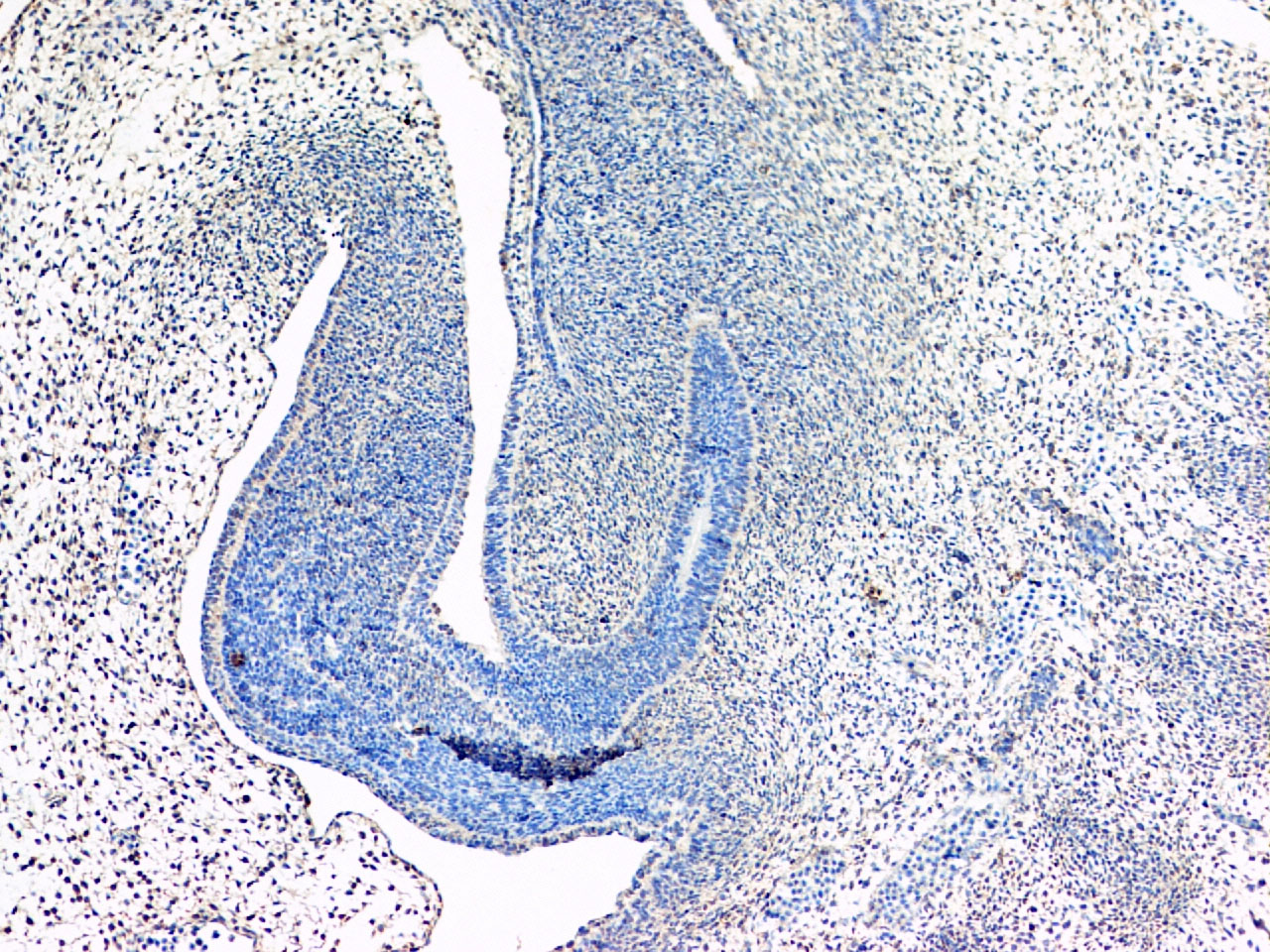

Supplement: Supplemental Information 3 [file peerj-04-1771-s003.zip › 4/c4-25-12 100╡≈╒√.jpg]

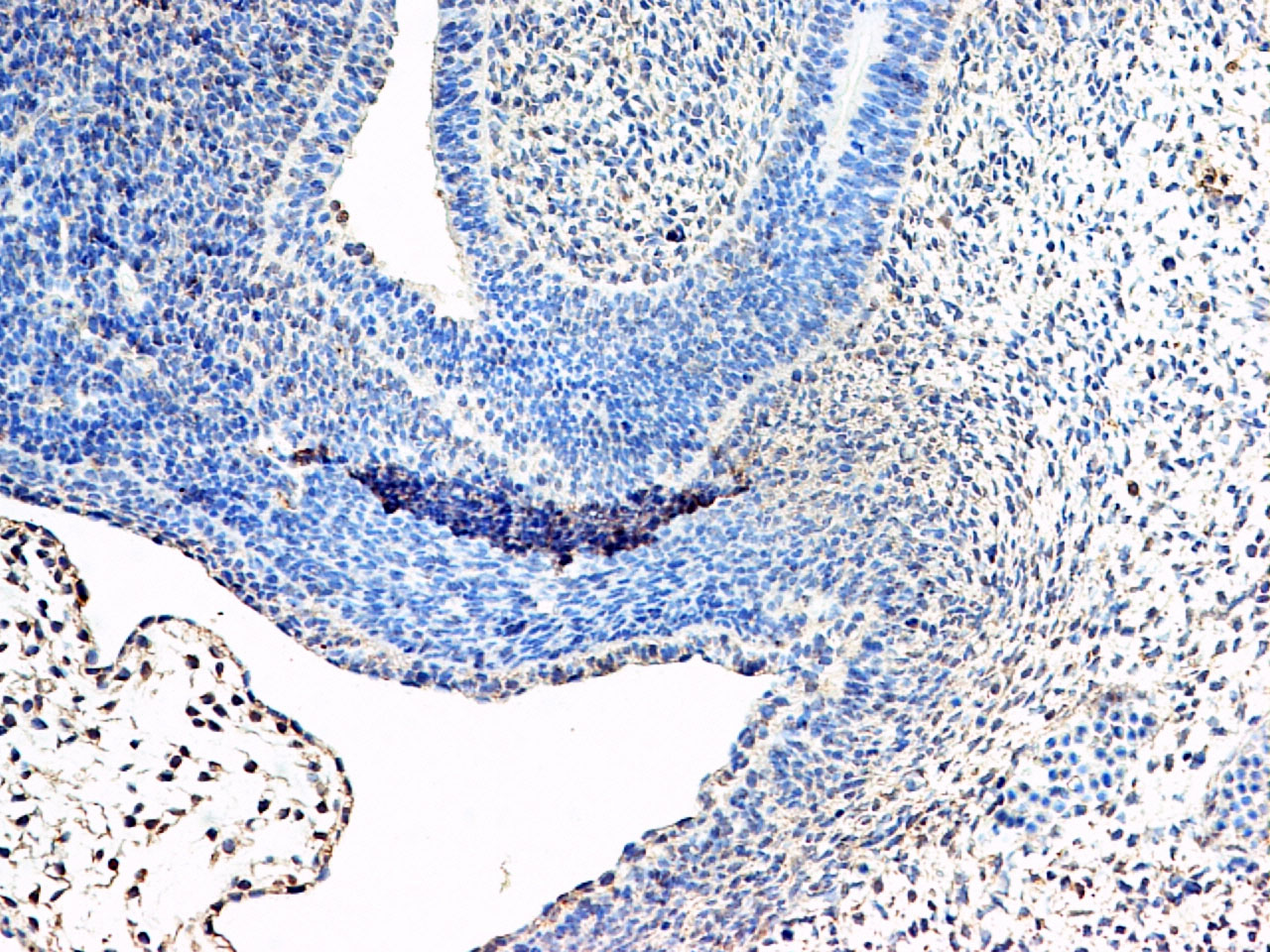

Supplement: Supplemental Information 3 [file peerj-04-1771-s003.zip › 4/c4-25-12 200╡≈╒√.jpg]

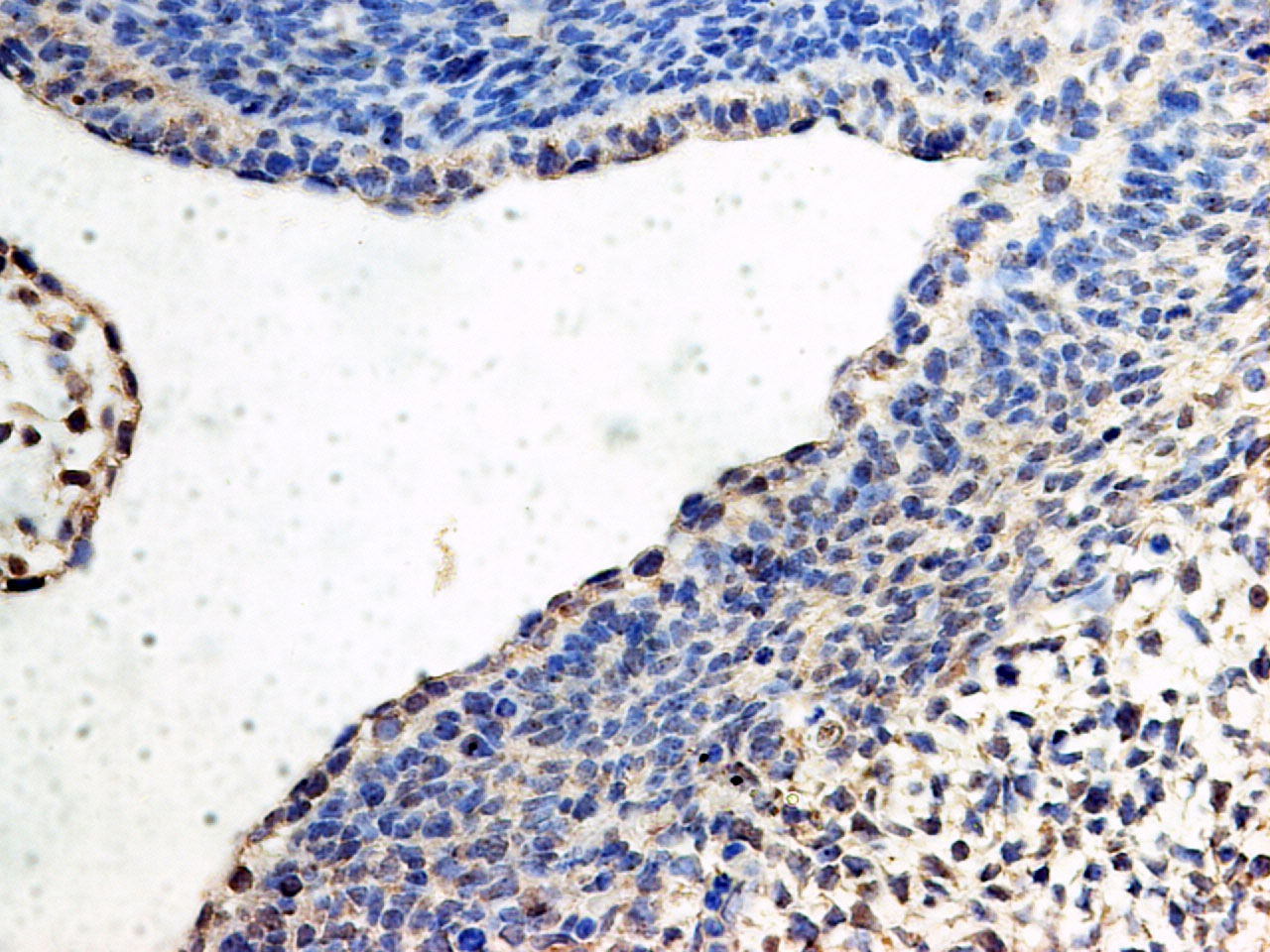

Supplement: Supplemental Information 3 [file peerj-04-1771-s003.zip › 4/c4-25-12 400''╡≈╒√.jpg]

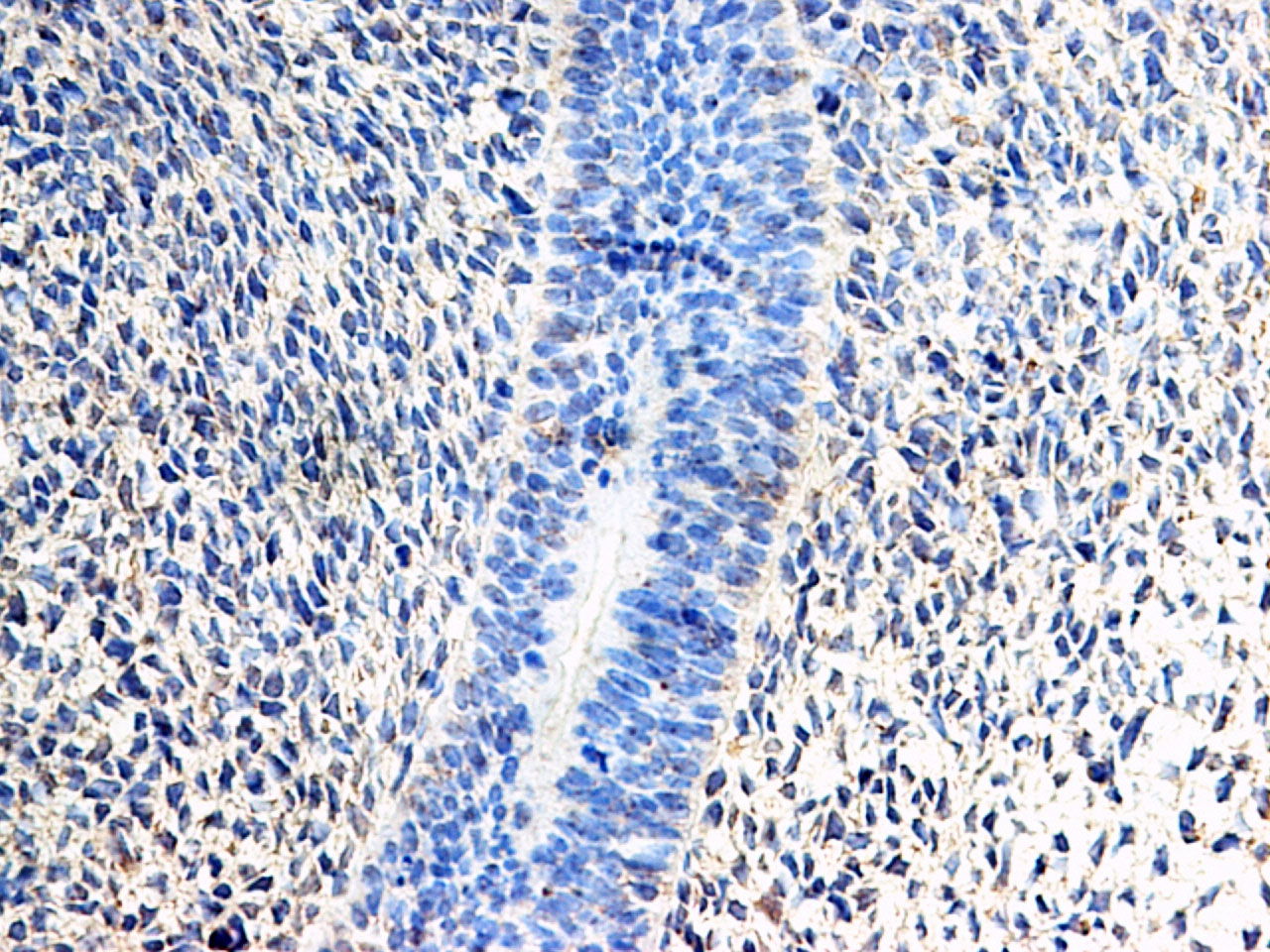

Supplement: Supplemental Information 3 [file peerj-04-1771-s003.zip › 4/c4-25-12 400'╡≈╒√.jpg]

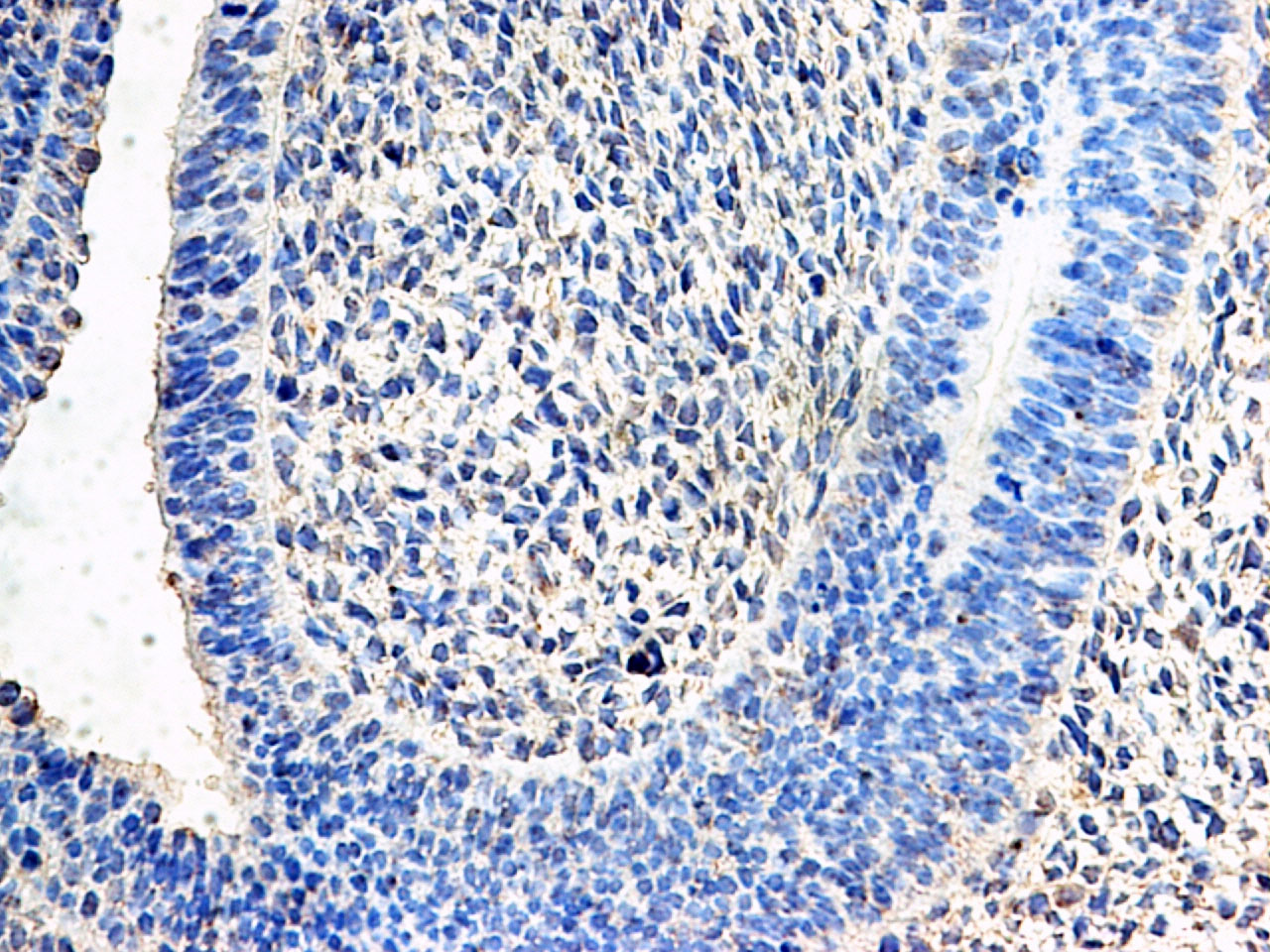

Supplement: Supplemental Information 3 [file peerj-04-1771-s003.zip › 4/c4-25-12 400╡≈╒√.jpg]

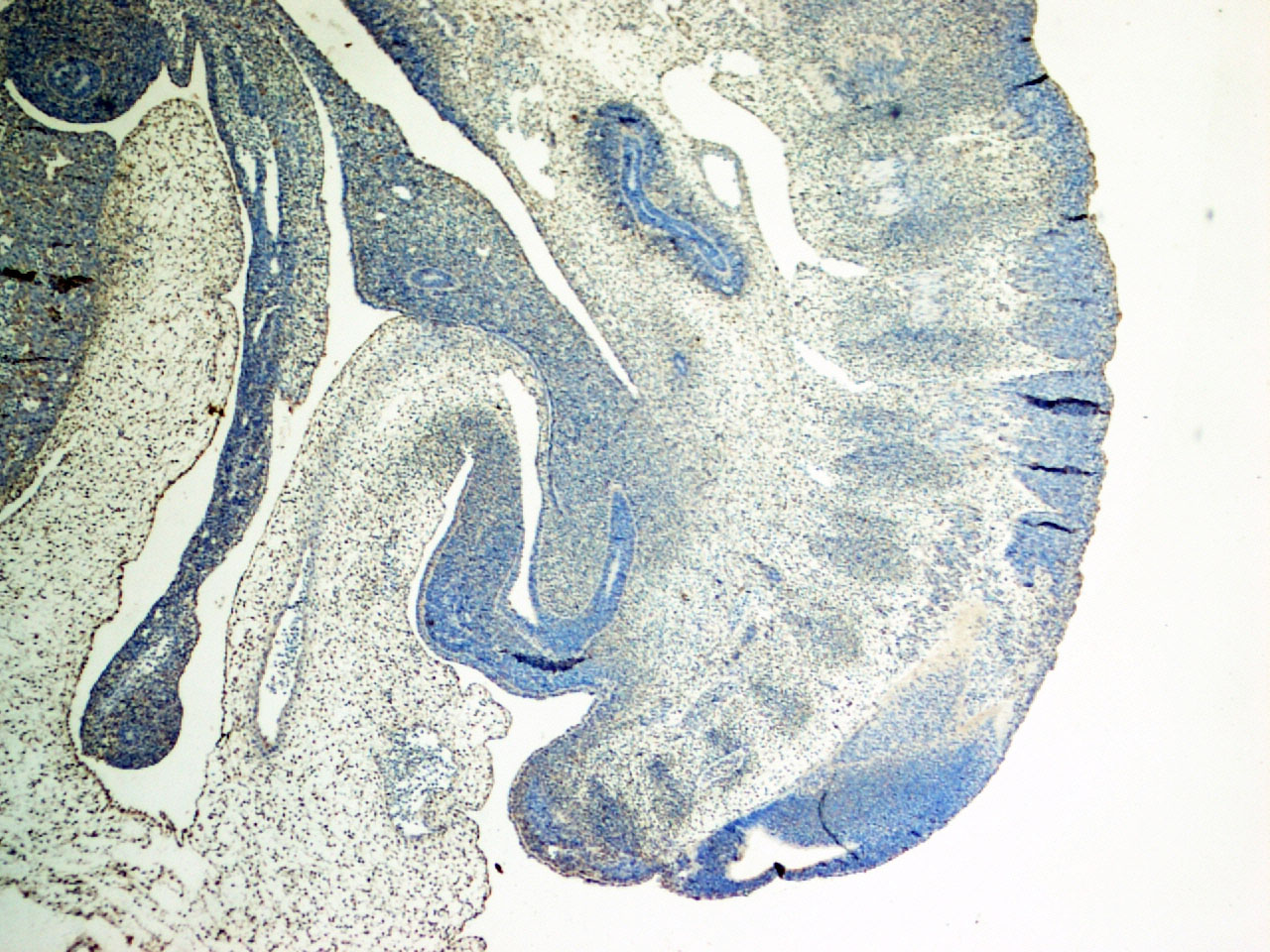

Supplement: Supplemental Information 3 [file peerj-04-1771-s003.zip › 4/c4-25-12 40╡≈╒√.jpg]

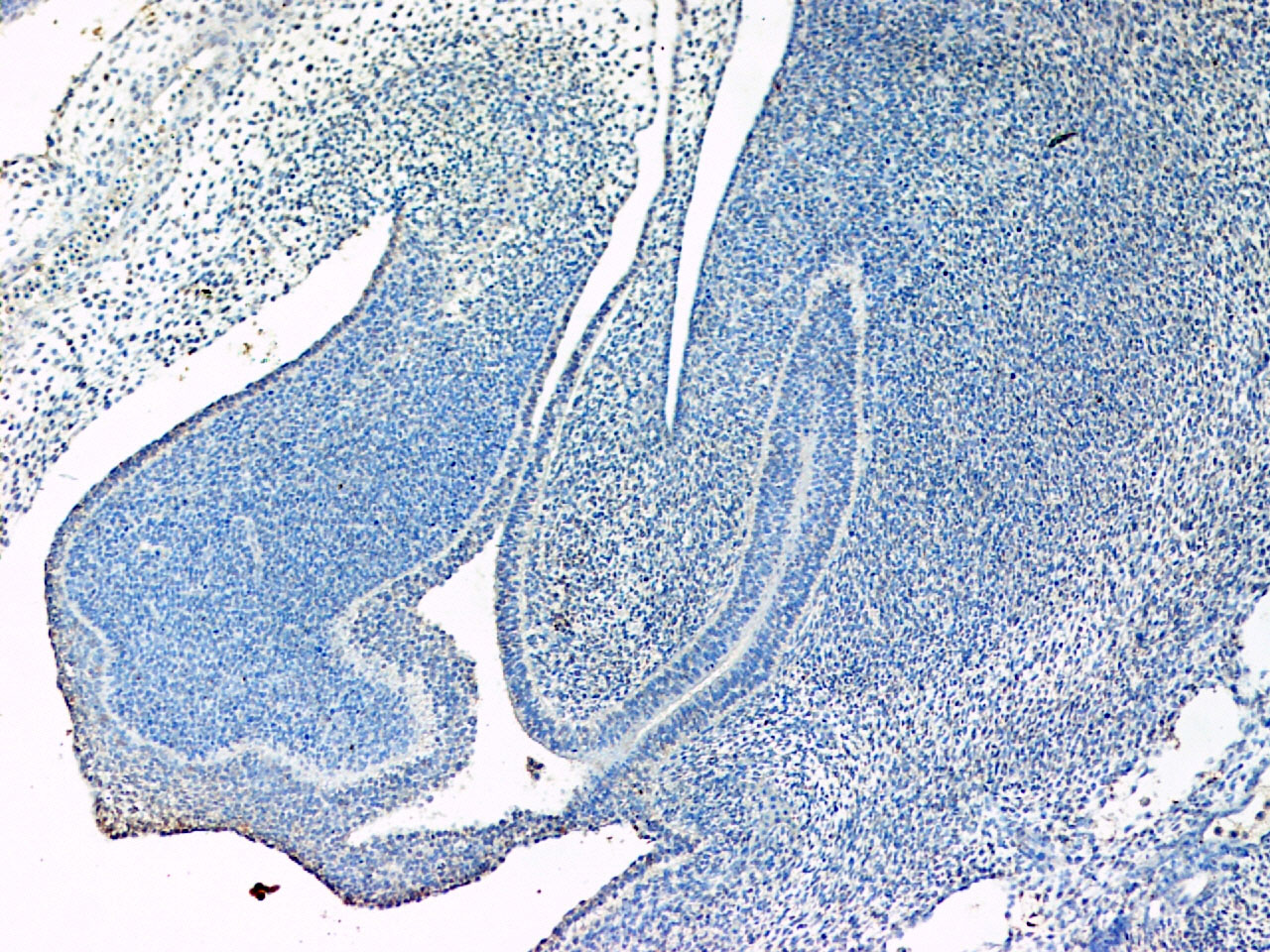

Supplement: Supplemental Information 3 [file peerj-04-1771-s003.zip › 4/c4-36-7 100╡≈╒√.jpg]

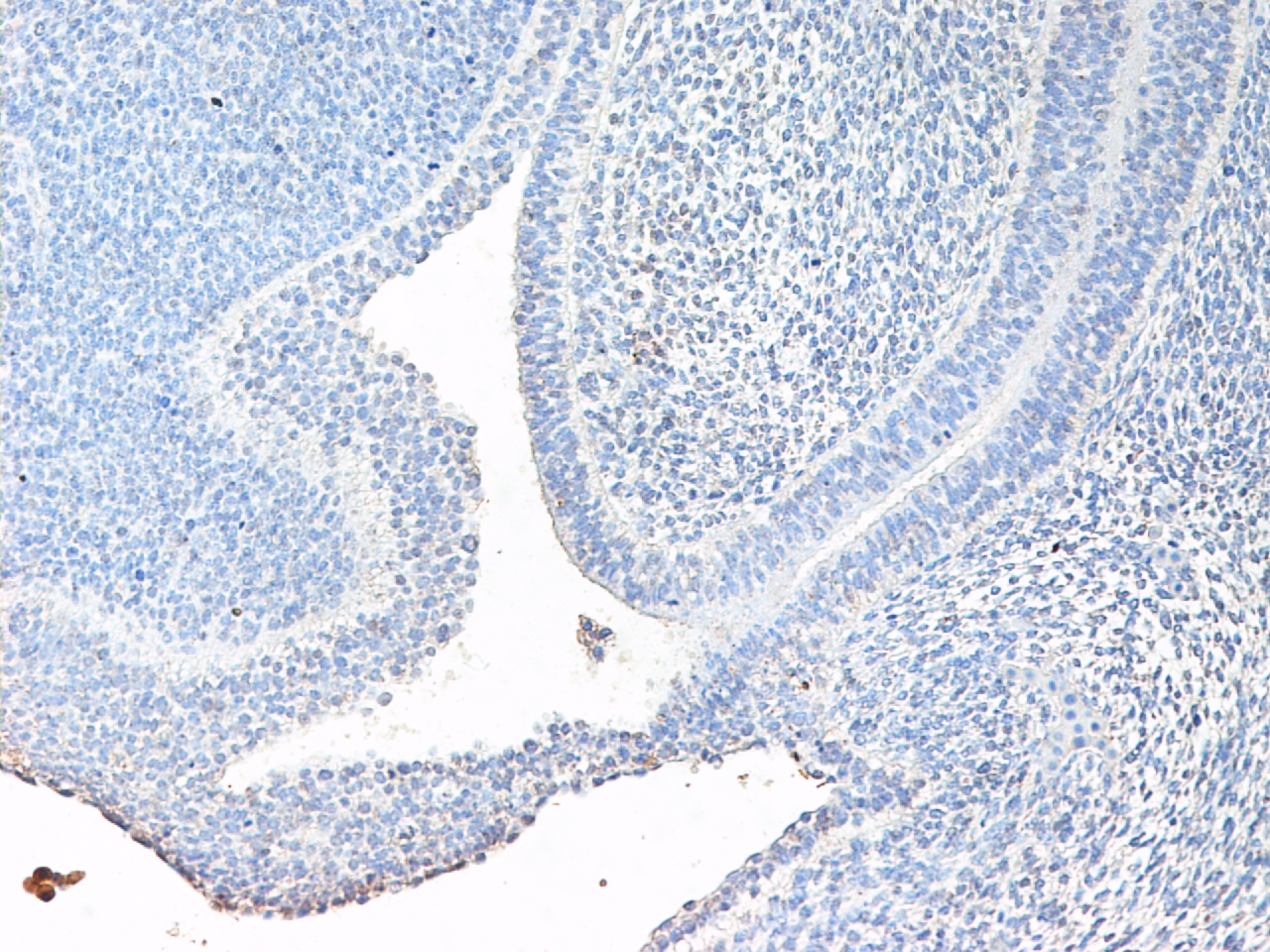

Supplement: Supplemental Information 3 [file peerj-04-1771-s003.zip › 4/c4-36-7 200'.jpg]

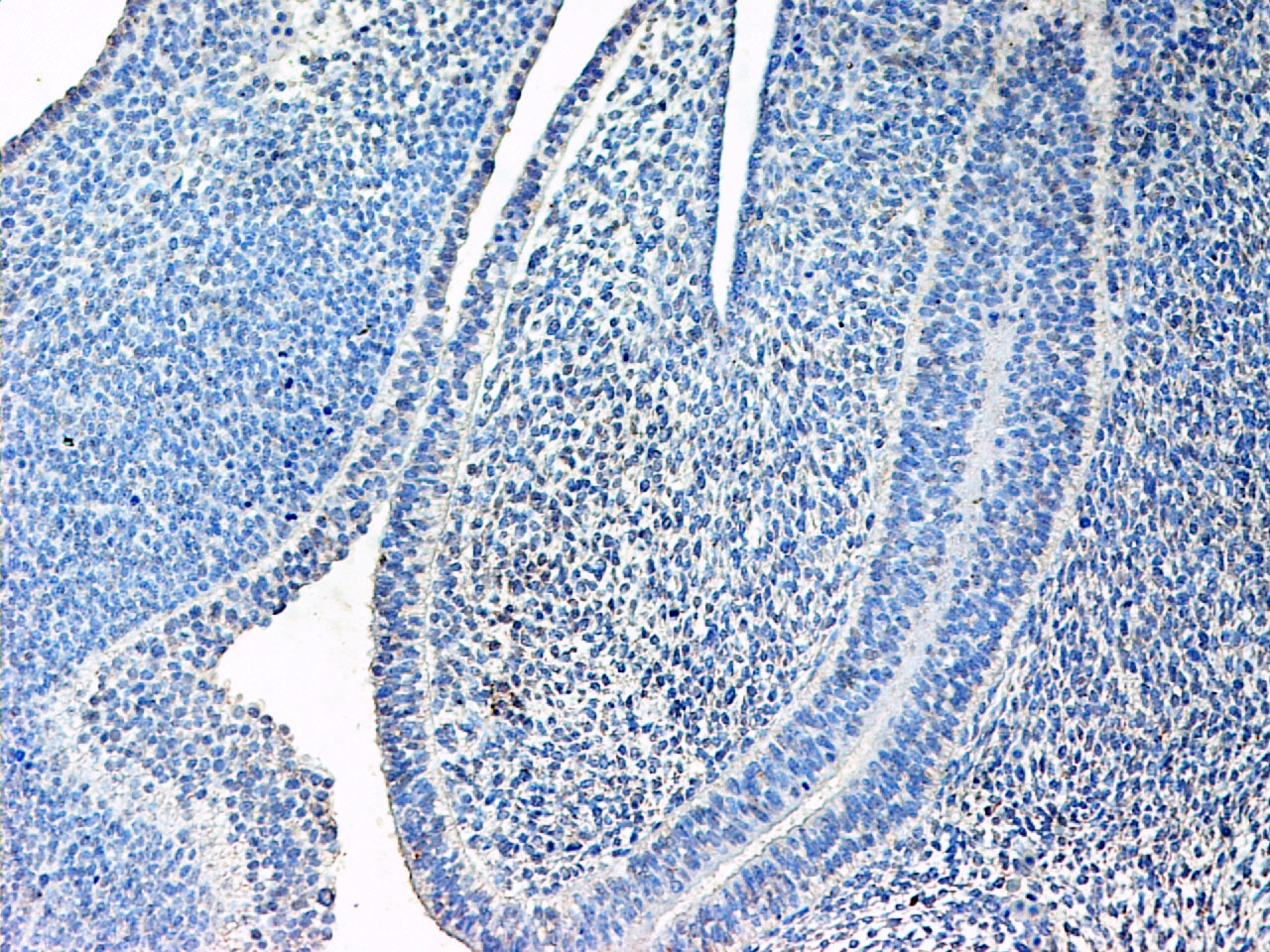

Supplement: Supplemental Information 3 [file peerj-04-1771-s003.zip › 4/c4-36-7 200╡≈╒√.jpg]

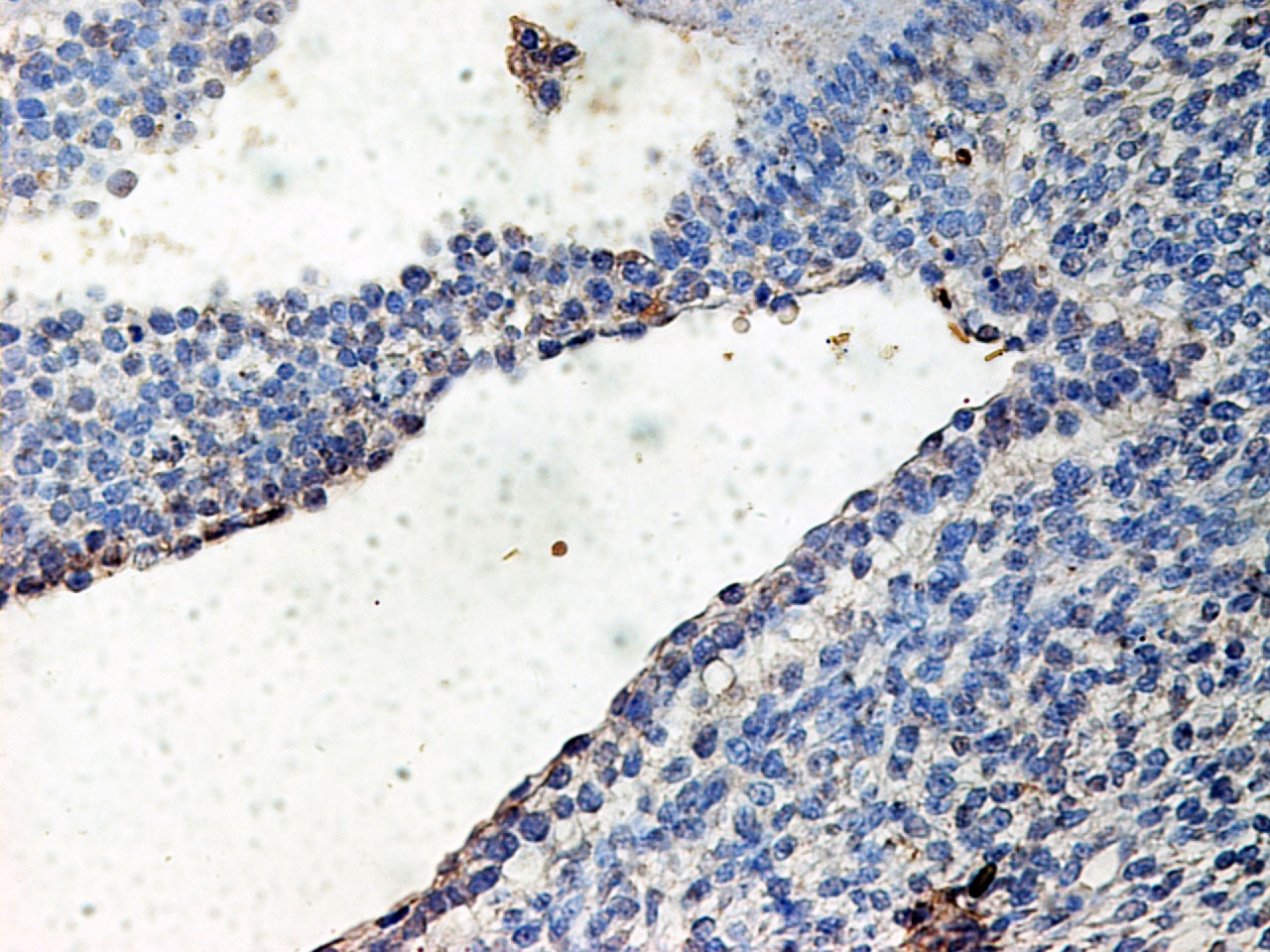

Supplement: Supplemental Information 3 [file peerj-04-1771-s003.zip › 4/c4-36-7 400'''╡≈╒√.jpg]

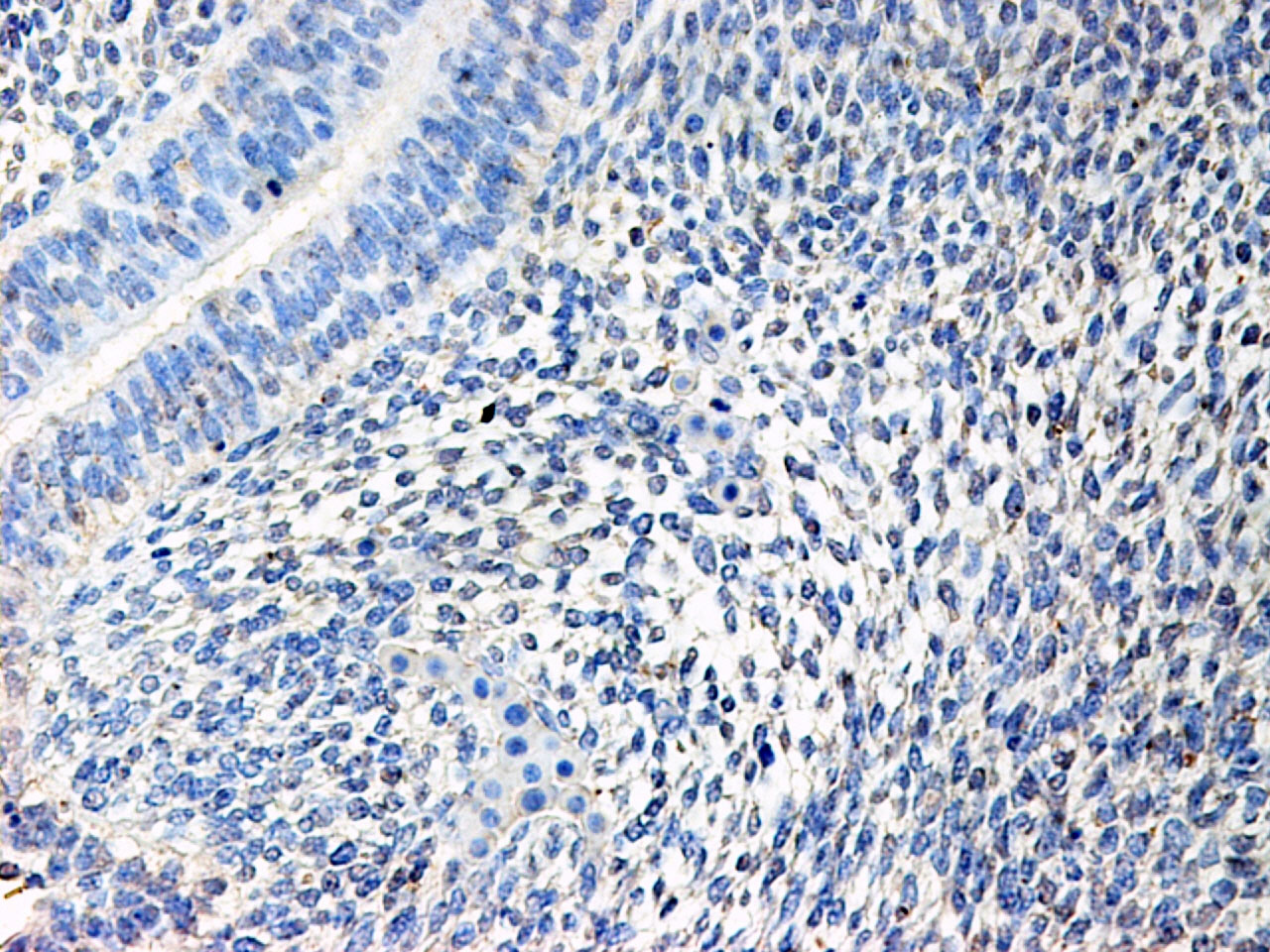

Supplement: Supplemental Information 3 [file peerj-04-1771-s003.zip › 4/c4-36-7 400''╡≈╒√.jpg]

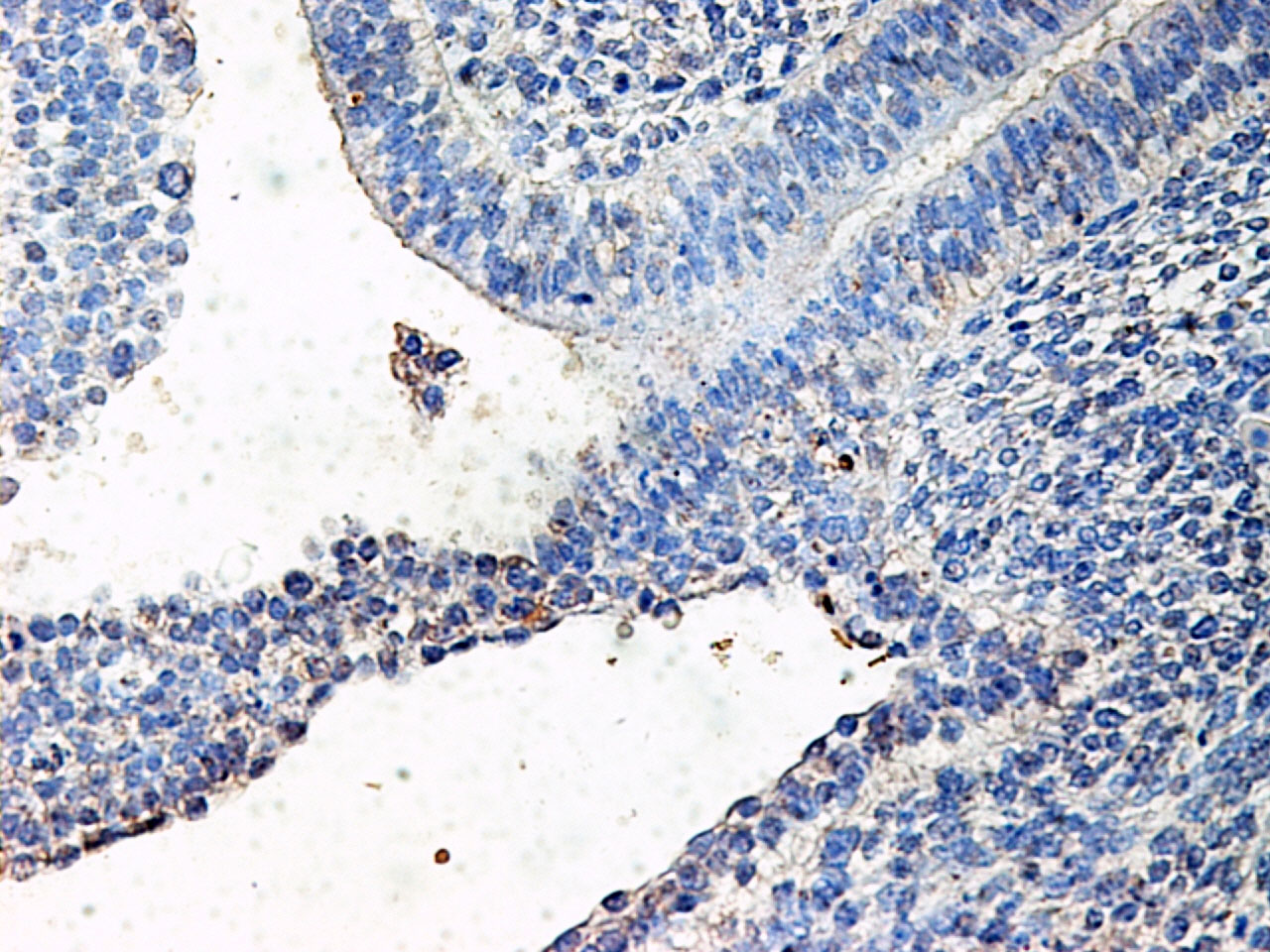

Supplement: Supplemental Information 3 [file peerj-04-1771-s003.zip › 4/c4-36-7 400'╡≈╒√.jpg]

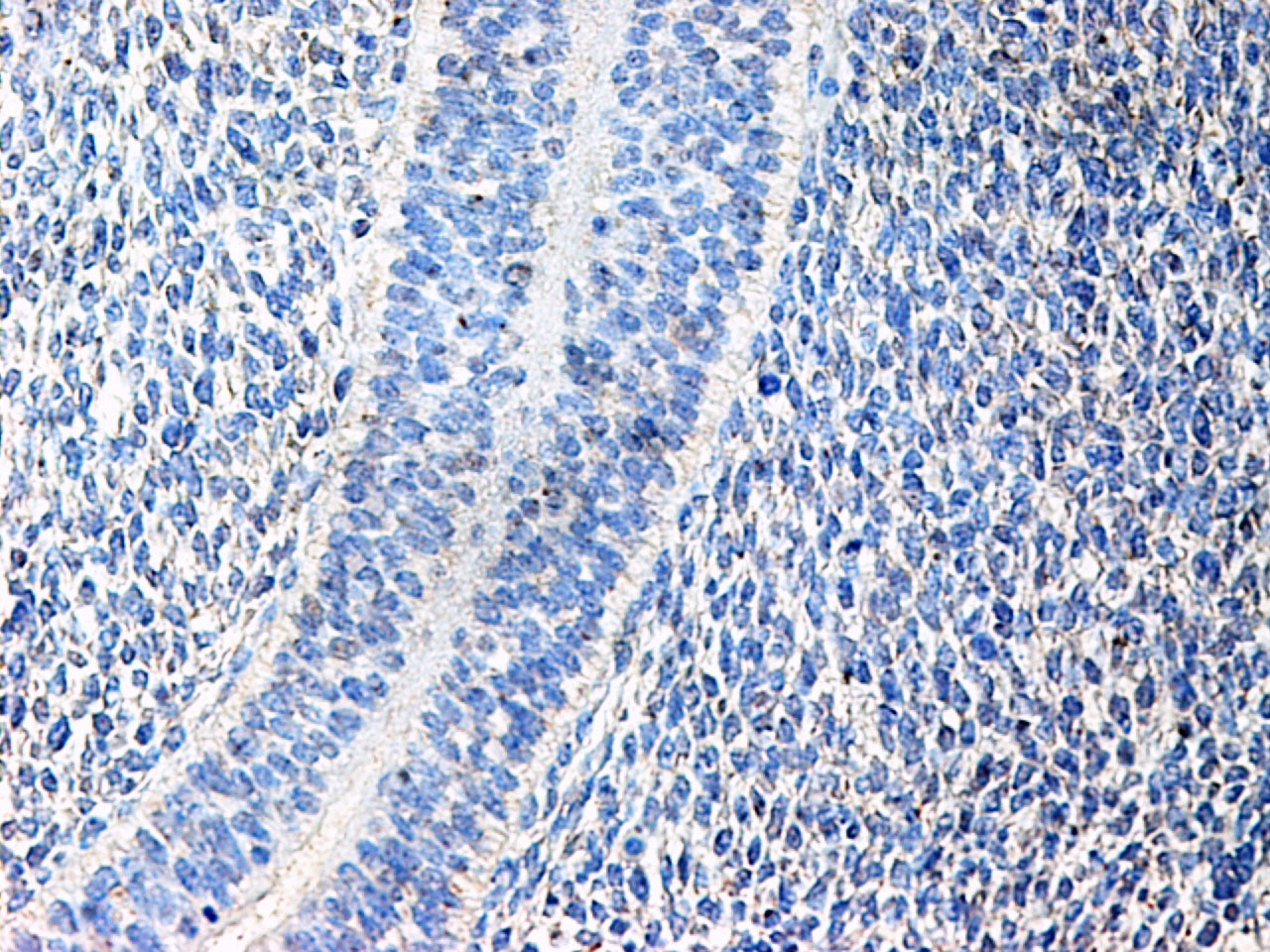

Supplement: Supplemental Information 3 [file peerj-04-1771-s003.zip › 4/c4-36-7 400╡≈╒√.jpg]

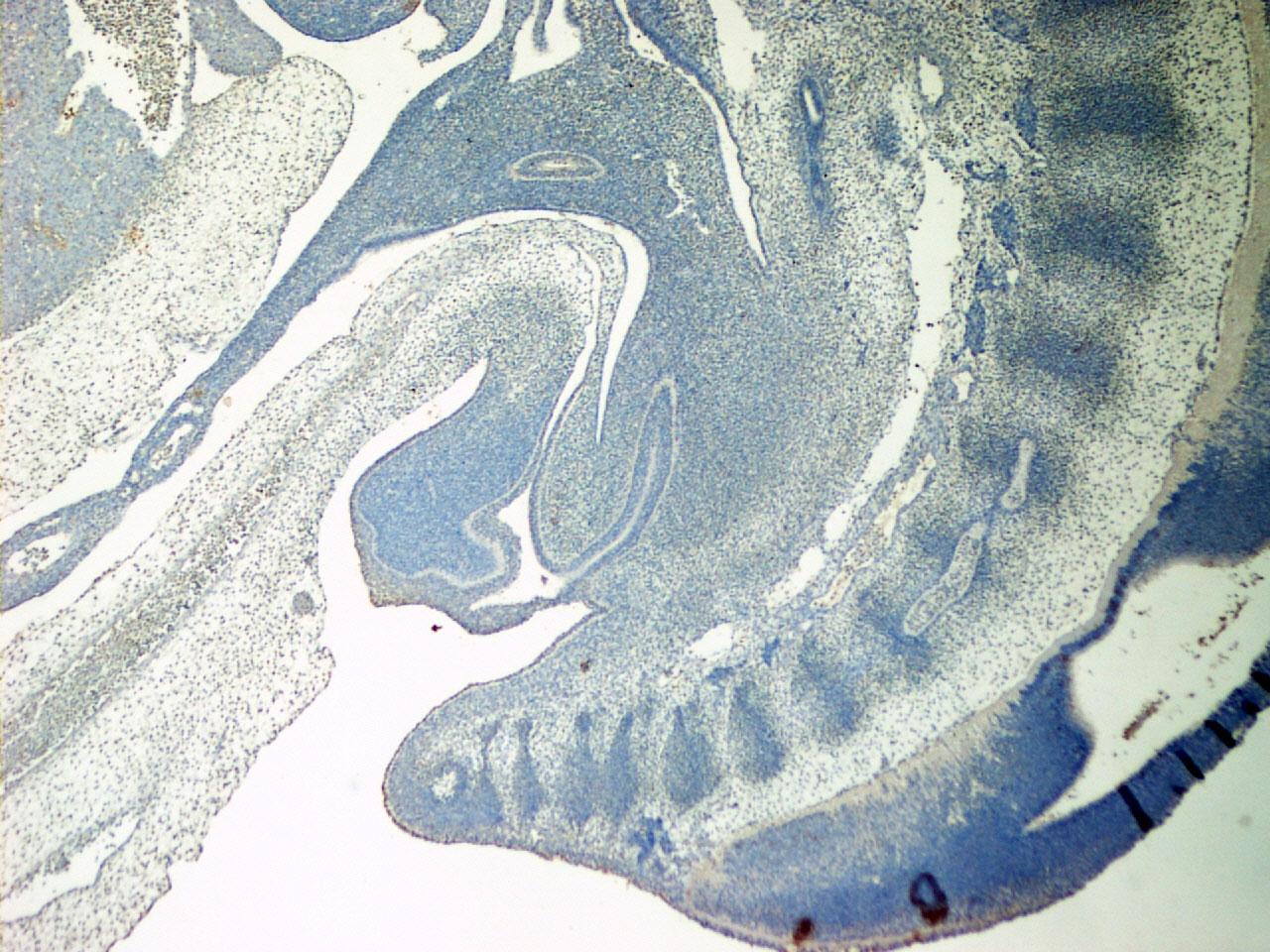

Supplement: Supplemental Information 3 [file peerj-04-1771-s003.zip › 4/c4-36-7 40╡≈╒√.jpg]

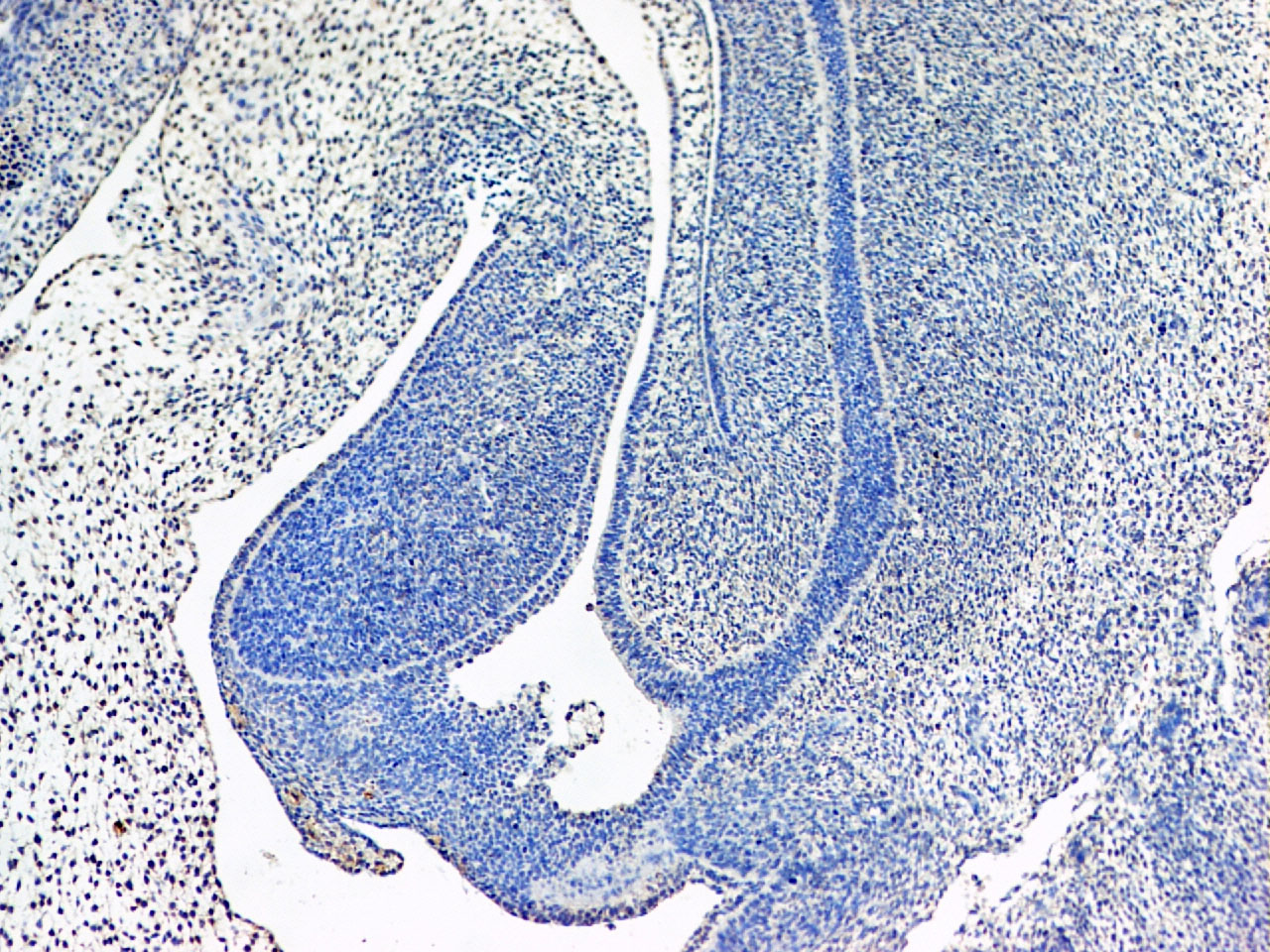

Supplement: Supplemental Information 3 [file peerj-04-1771-s003.zip › 4/c4-37-11 100╡≈╒√.jpg]

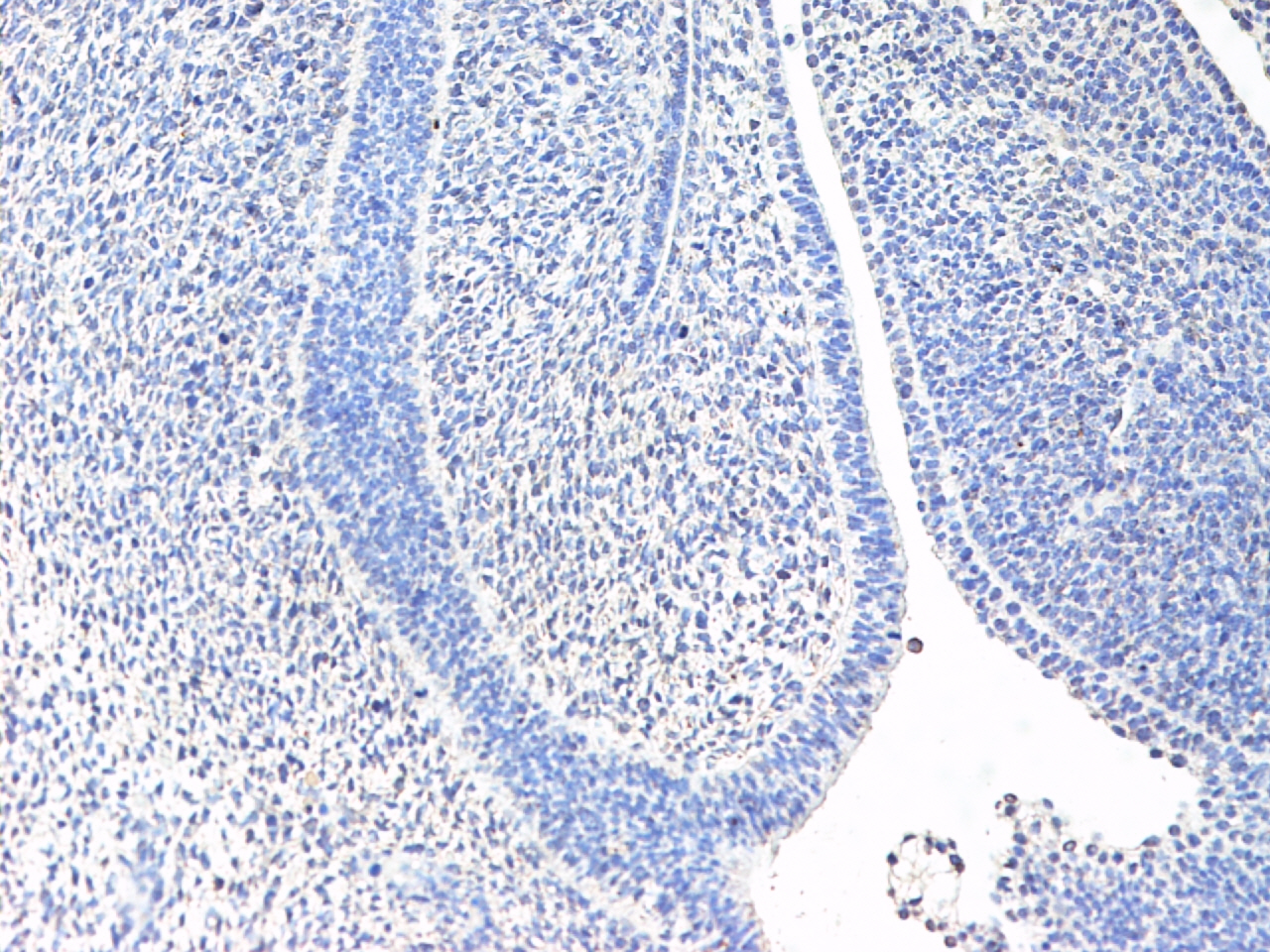

Supplement: Supplemental Information 3 [file peerj-04-1771-s003.zip › 4/c4-37-11 200'.jpg]

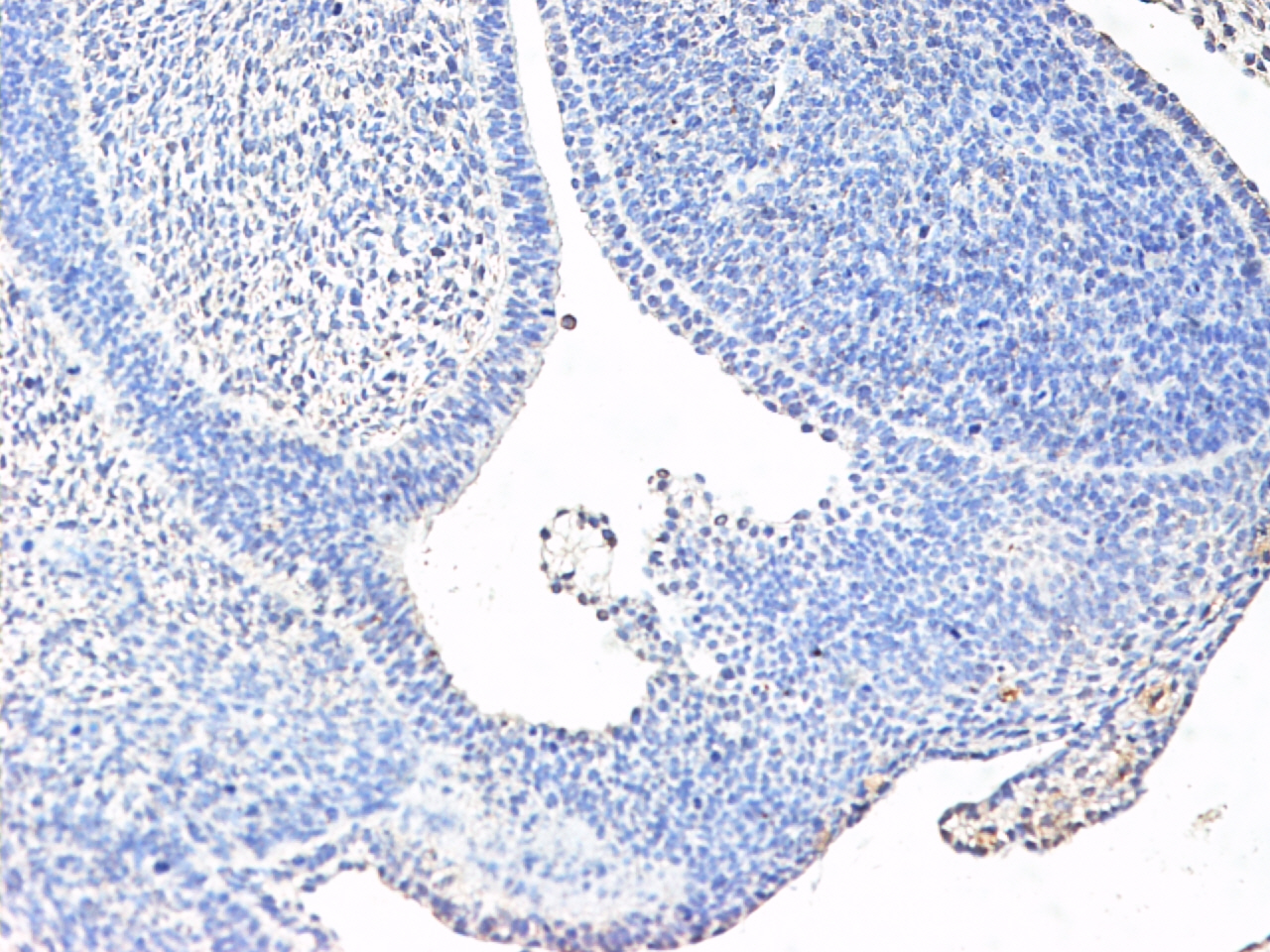

Supplement: Supplemental Information 3 [file peerj-04-1771-s003.zip › 4/c4-37-11 200.jpg]

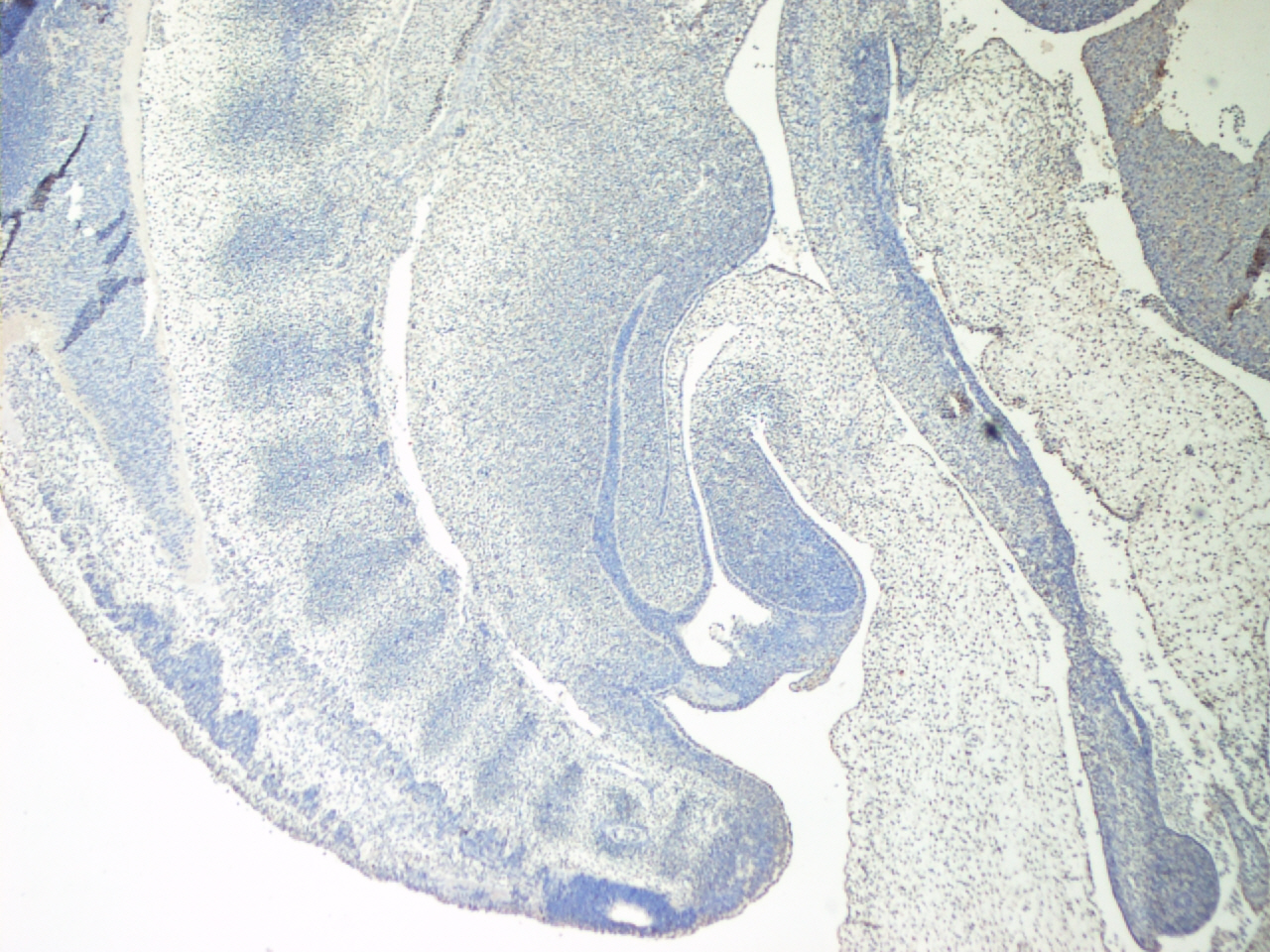

Supplement: Supplemental Information 3 [file peerj-04-1771-s003.zip › 4/c4-37-11 40.jpg]

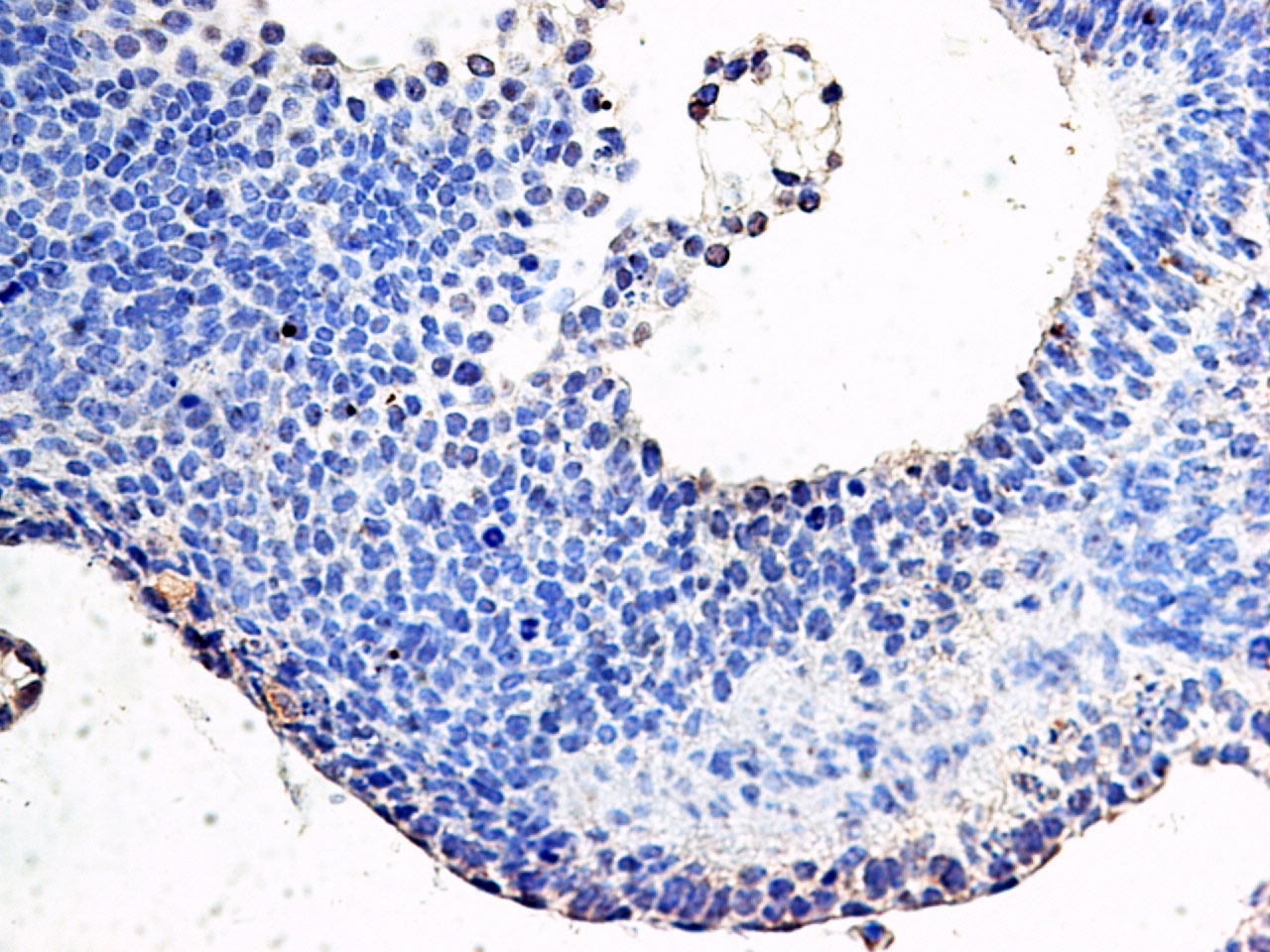

Supplement: Supplemental Information 3 [file peerj-04-1771-s003.zip › 4/c4-37-11 400'''╡≈╒√.jpg]

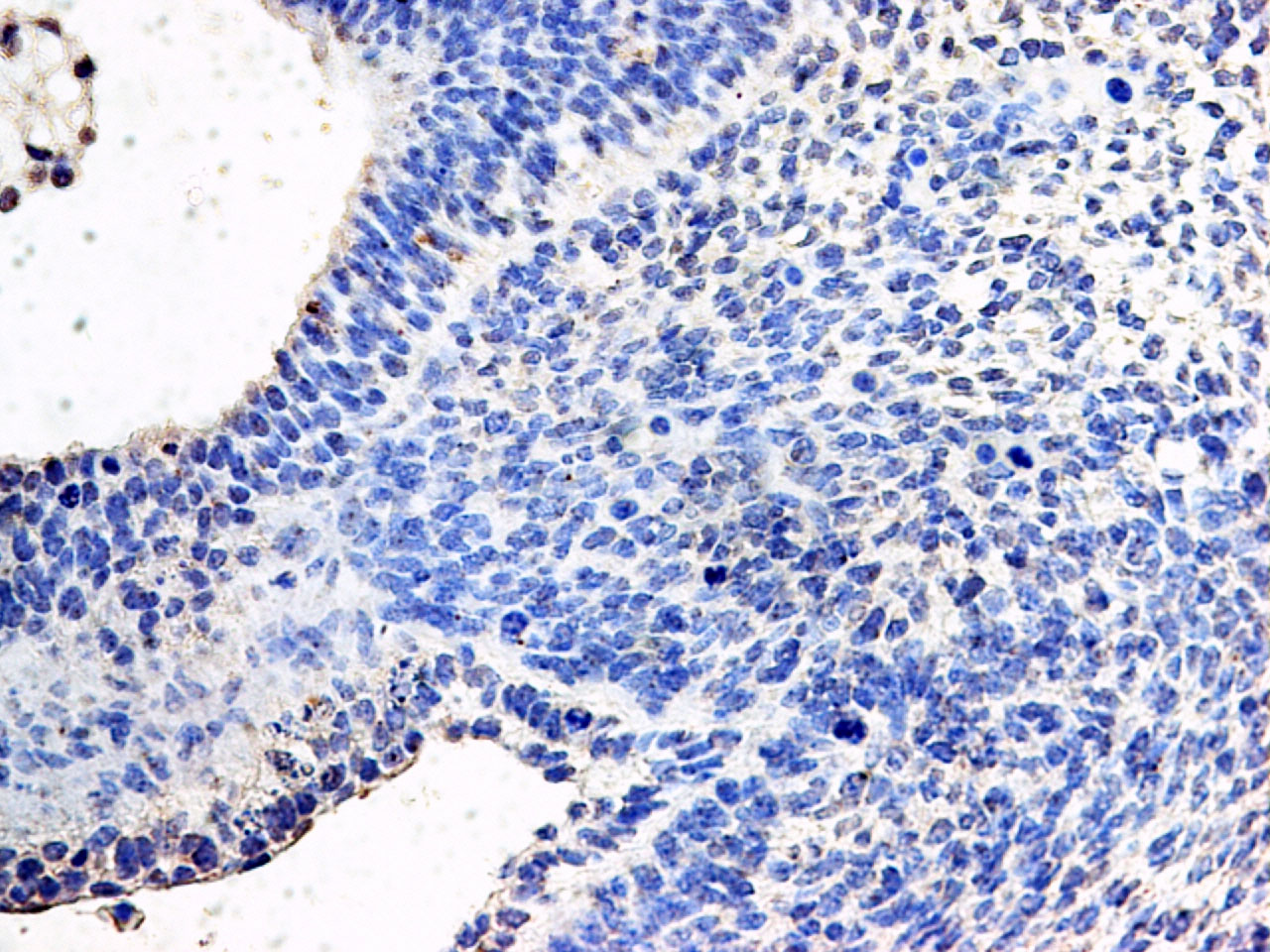

Supplement: Supplemental Information 3 [file peerj-04-1771-s003.zip › 4/c4-37-11 400''╡≈╒√.jpg]

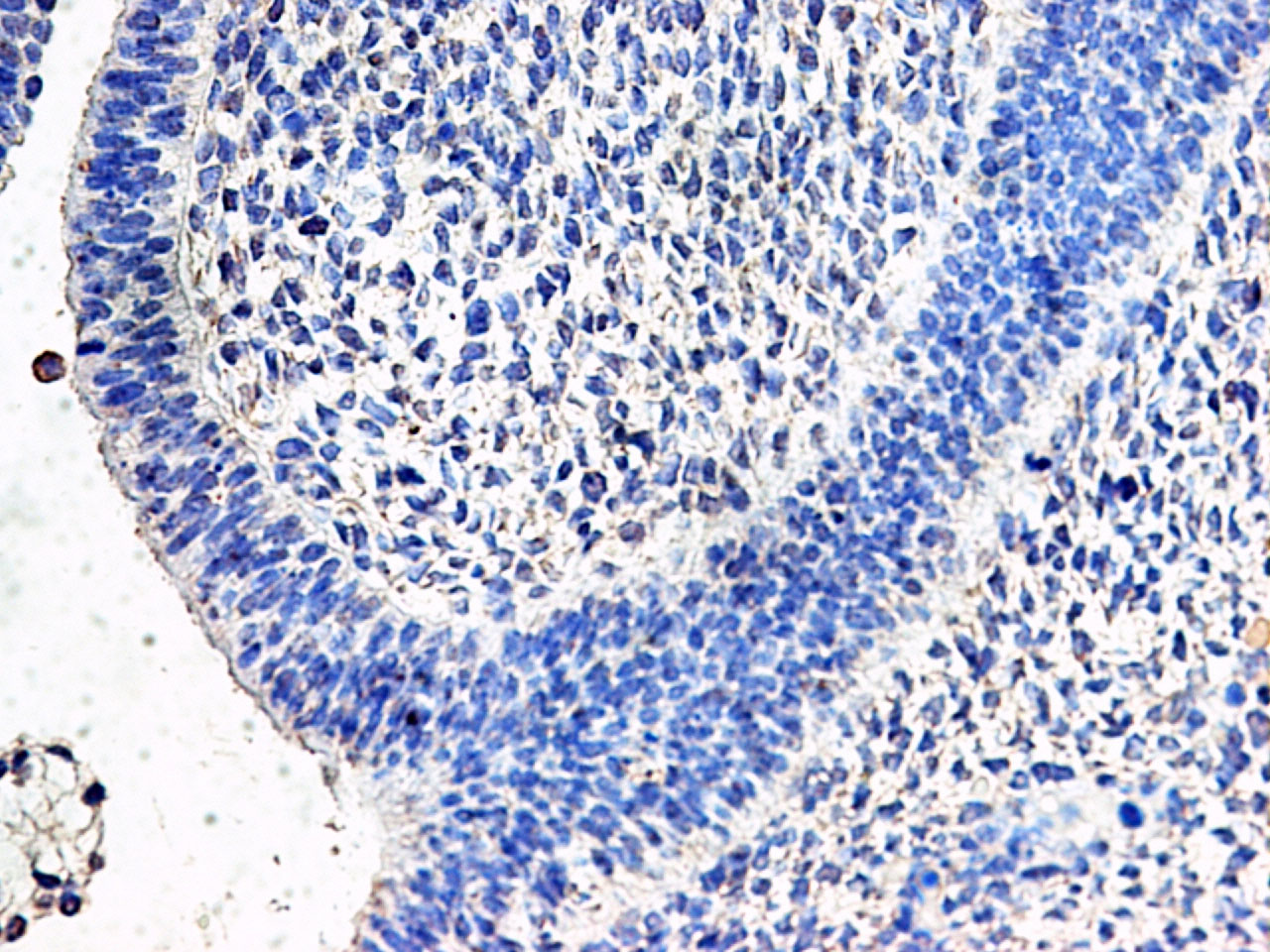

Supplement: Supplemental Information 3 [file peerj-04-1771-s003.zip › 4/c4-37-11 400'╡≈╒√.jpg]

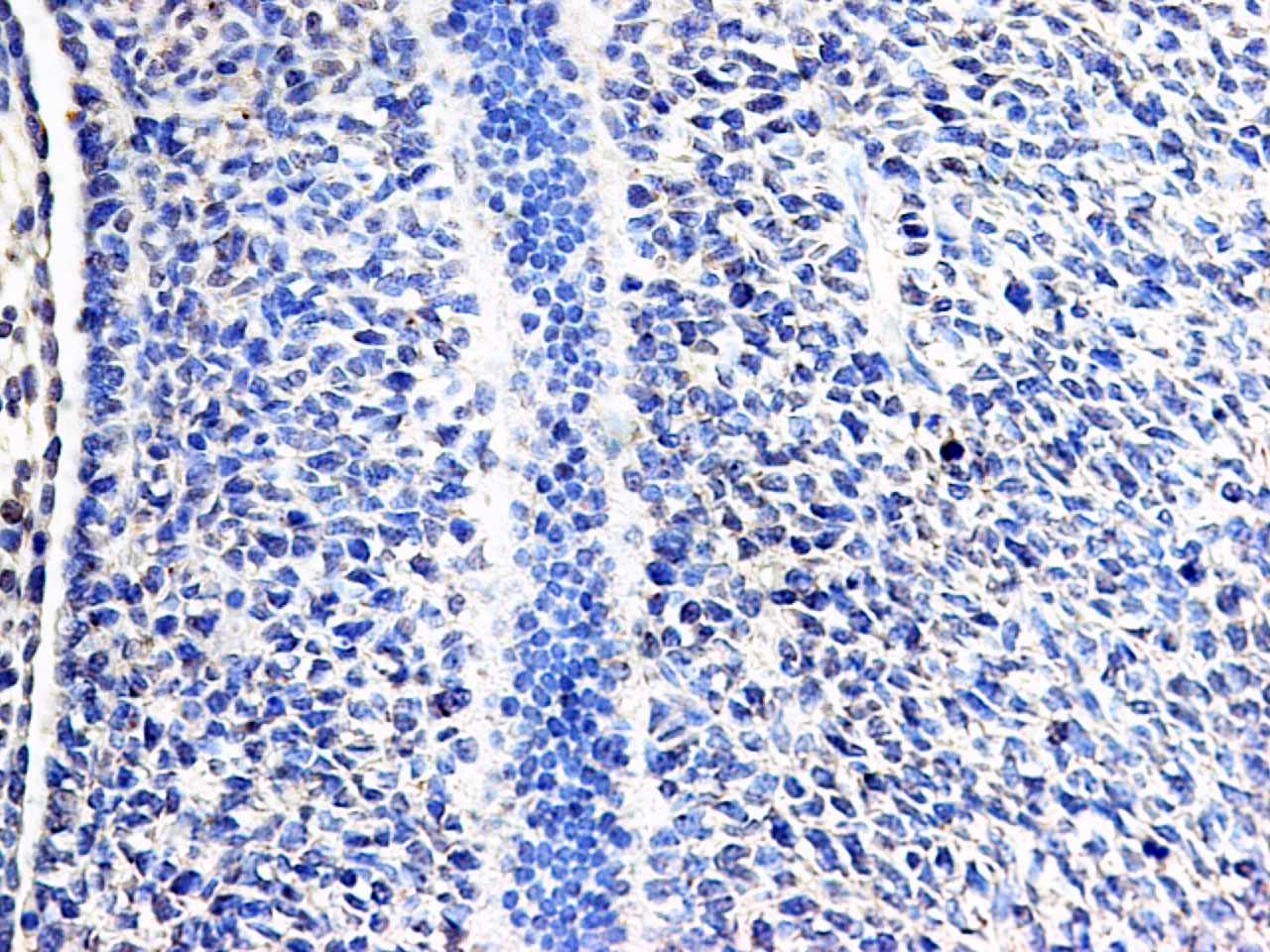

Supplement: Supplemental Information 3 [file peerj-04-1771-s003.zip › 4/c4-37-11 400╡≈╒√.jpg]
