# Supplementary figures and images for: Spatiotemporal distribution of caudal-type homeobox proteins during development of the hindgut and anorectum in human embryos (part 2 of 2)
Source: PeerJ. 2016 Mar 24;4:e1771. doi: 10.7717/peerj.1771 (PMC4811170; doi:10.7717/peerj.1771)

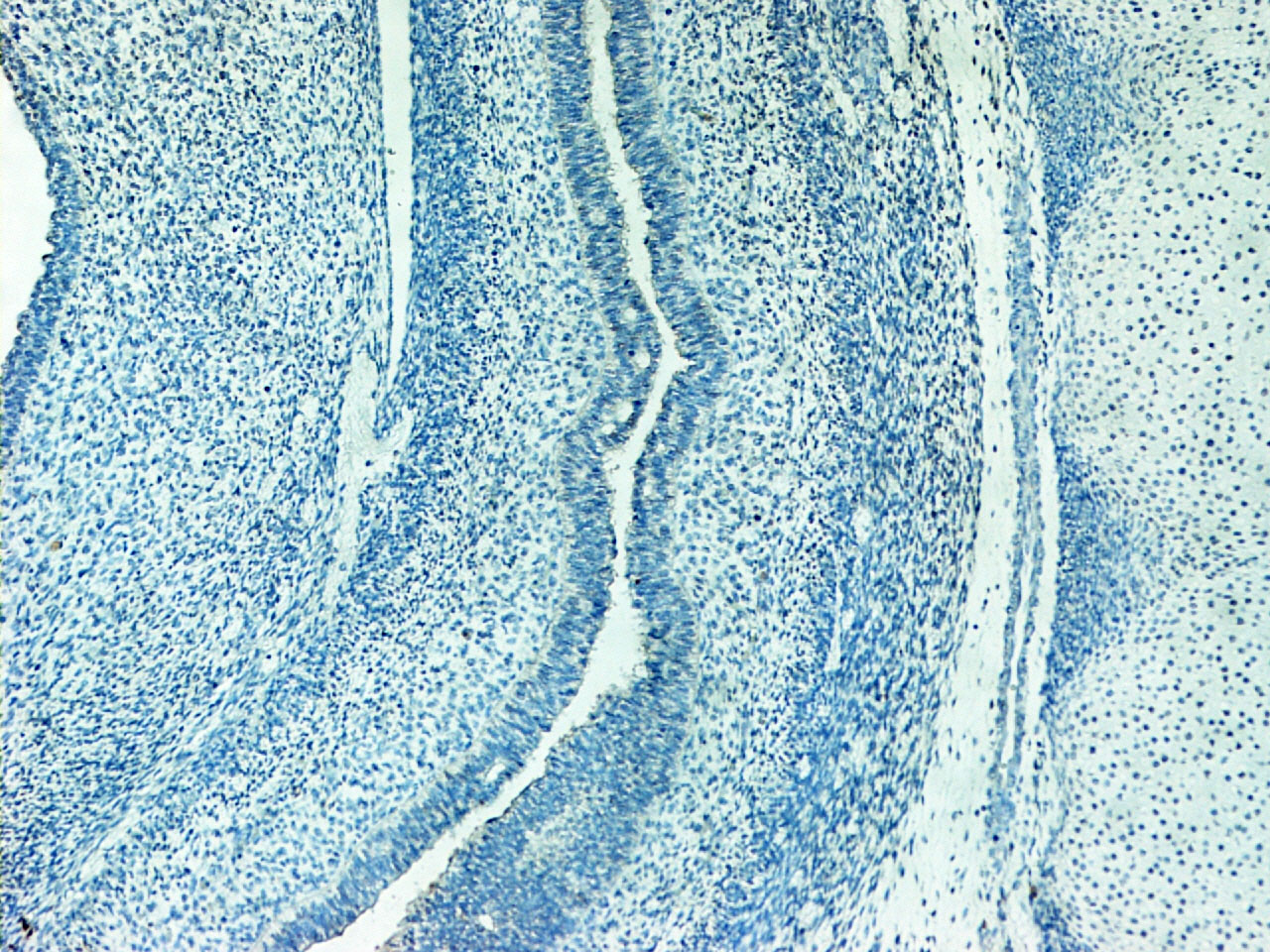

Supplement: Supplemental Information 3 [file peerj-04-1771-s003.zip › 4/c4-50-98-100▒╢'╡≈╒√.jpg]

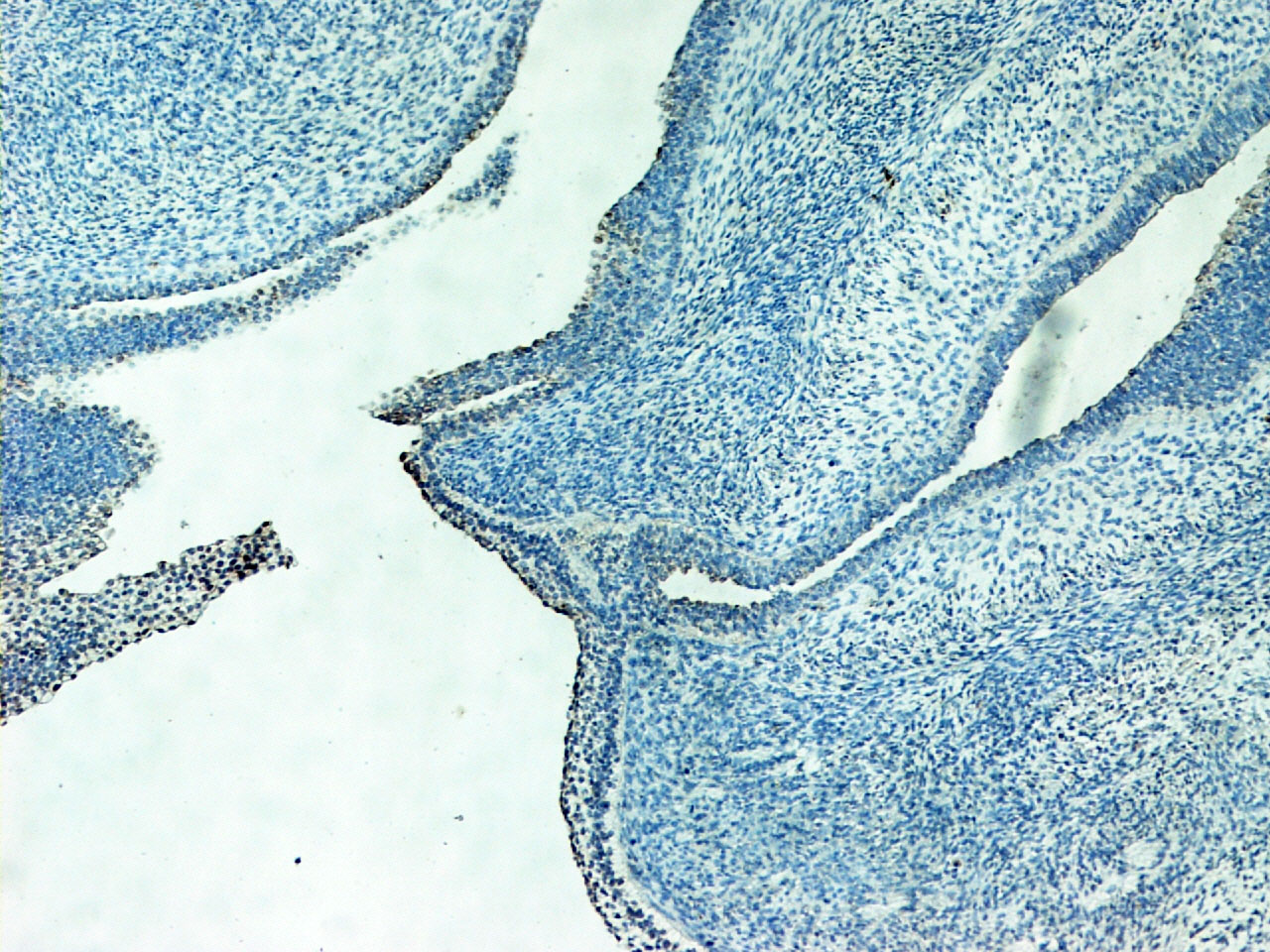

Supplement: Supplemental Information 3 [file peerj-04-1771-s003.zip › 4/c4-50-98-100▒╢╡≈╒√.jpg]

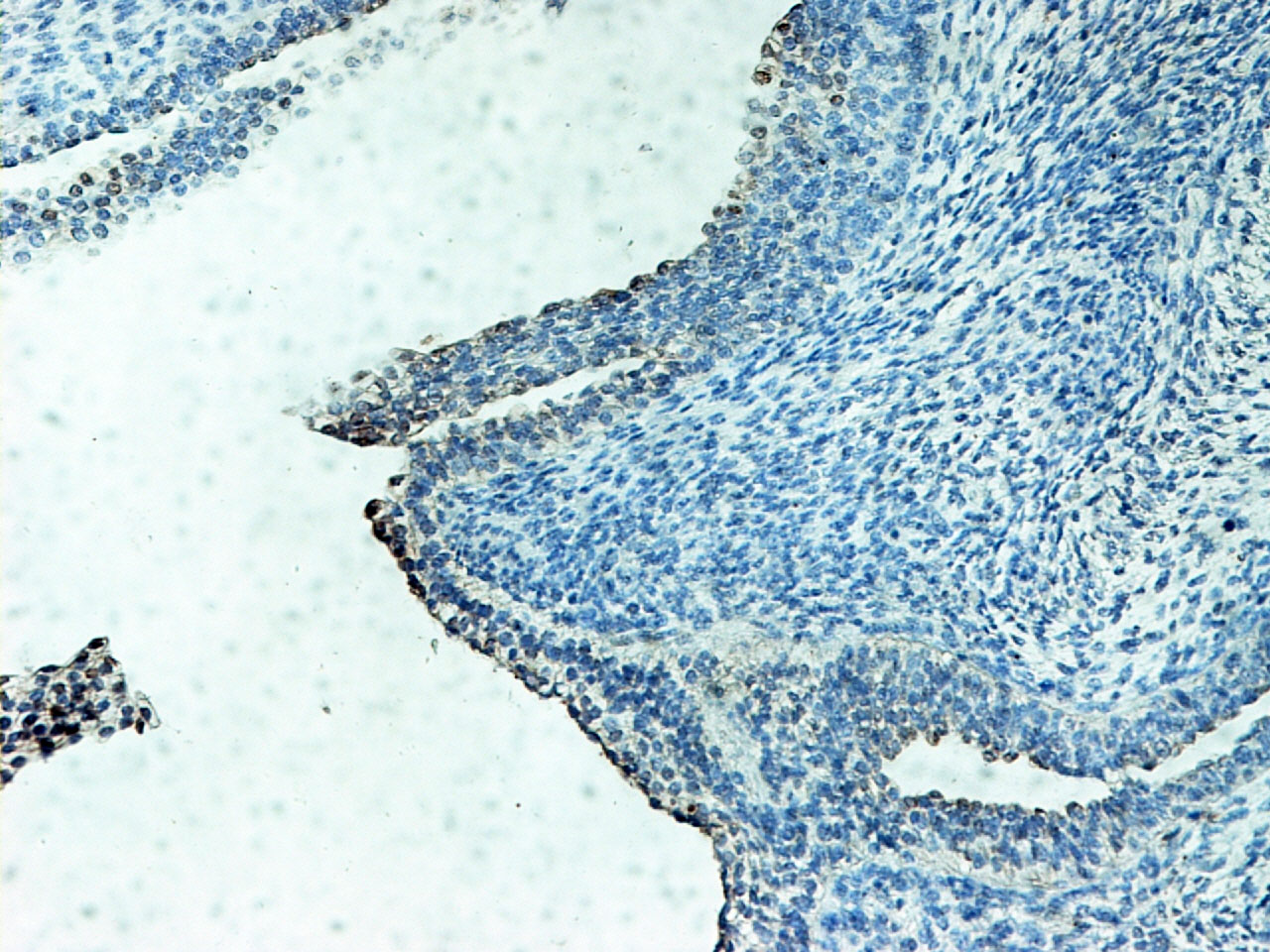

Supplement: Supplemental Information 3 [file peerj-04-1771-s003.zip › 4/c4-50-98-200▒╢''╡≈╒√.jpg]

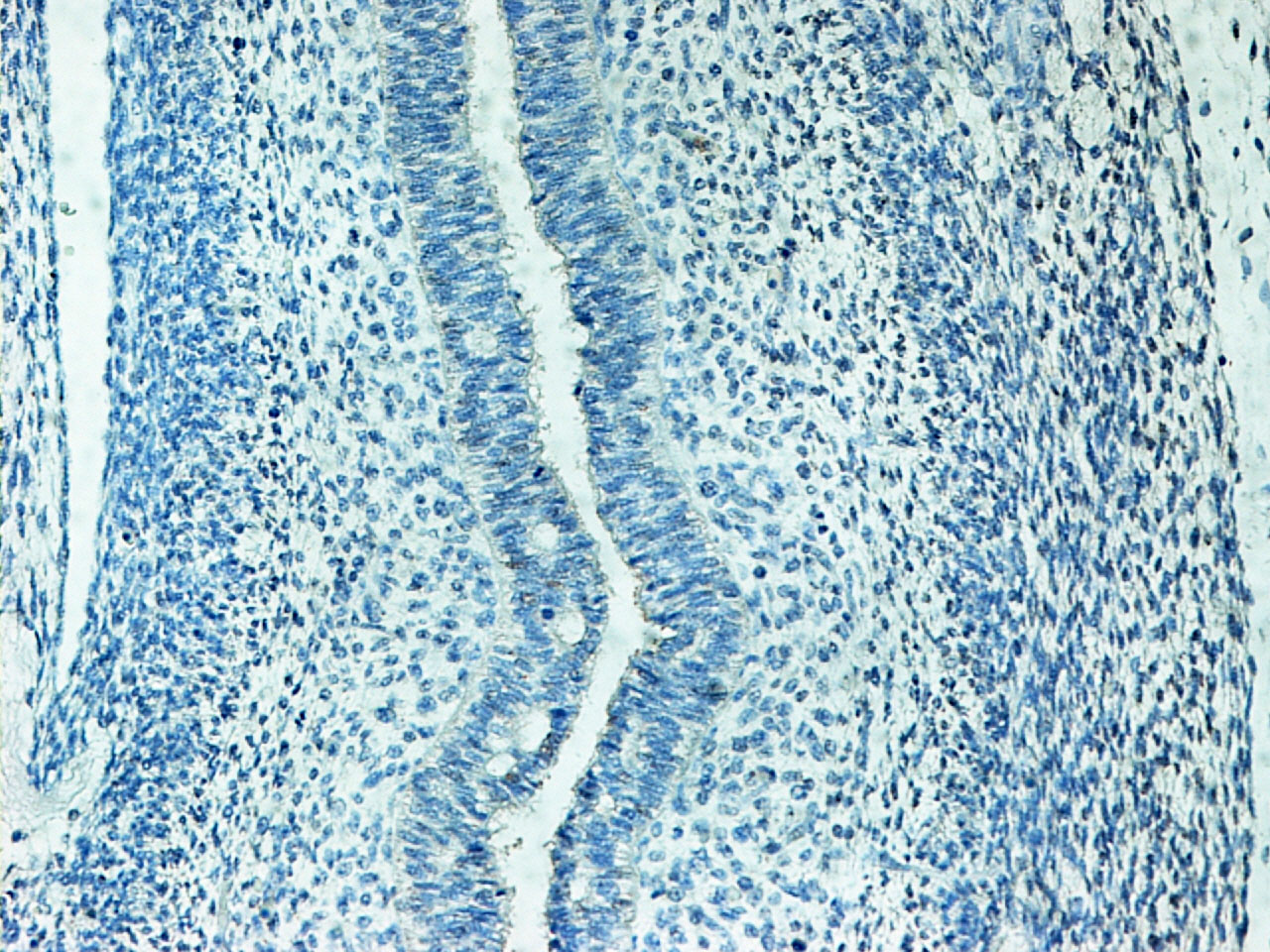

Supplement: Supplemental Information 3 [file peerj-04-1771-s003.zip › 4/c4-50-98-200▒╢'╡≈╒√.jpg]

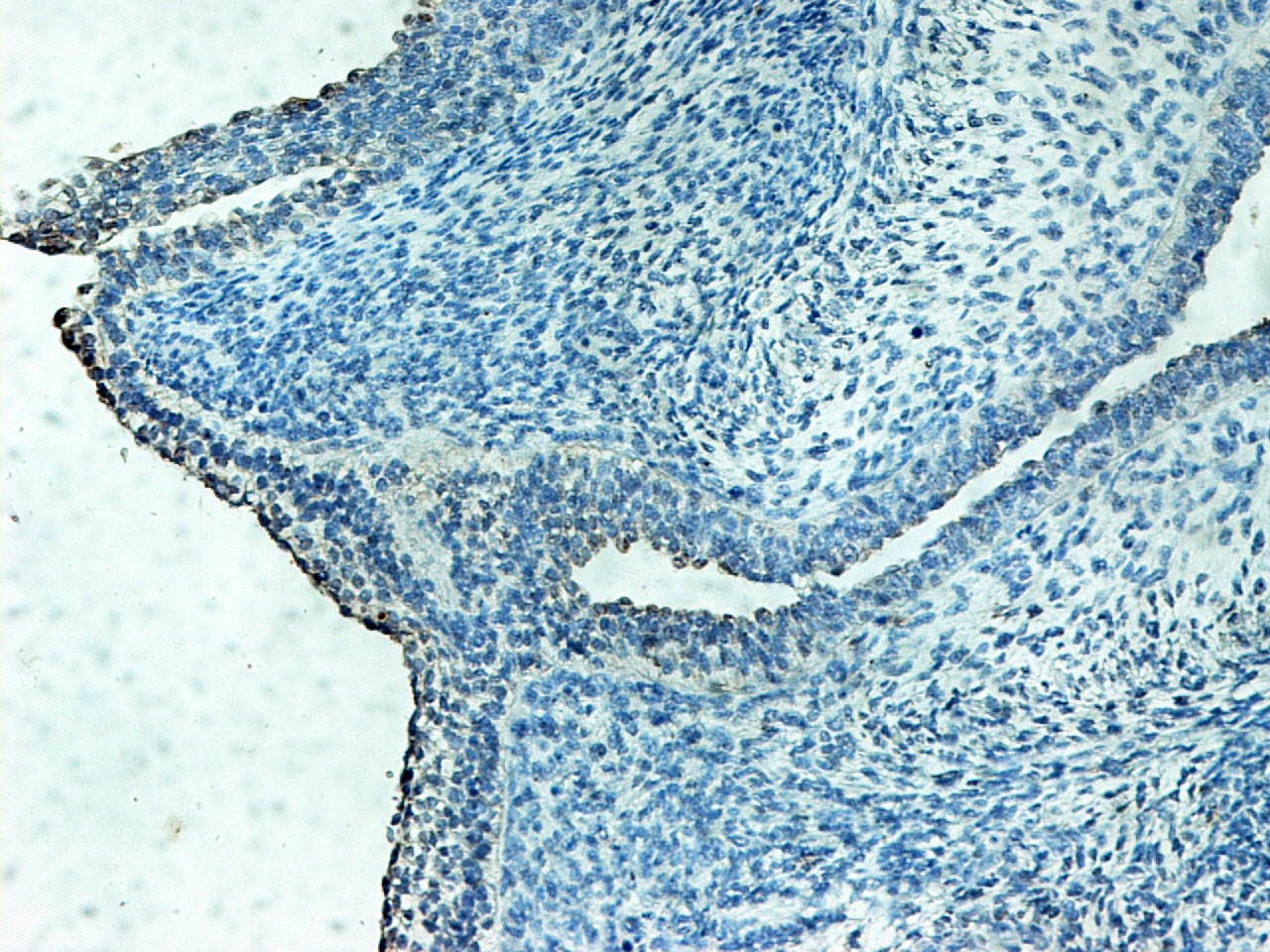

Supplement: Supplemental Information 3 [file peerj-04-1771-s003.zip › 4/c4-50-98-200▒╢╡≈╒√.jpg]

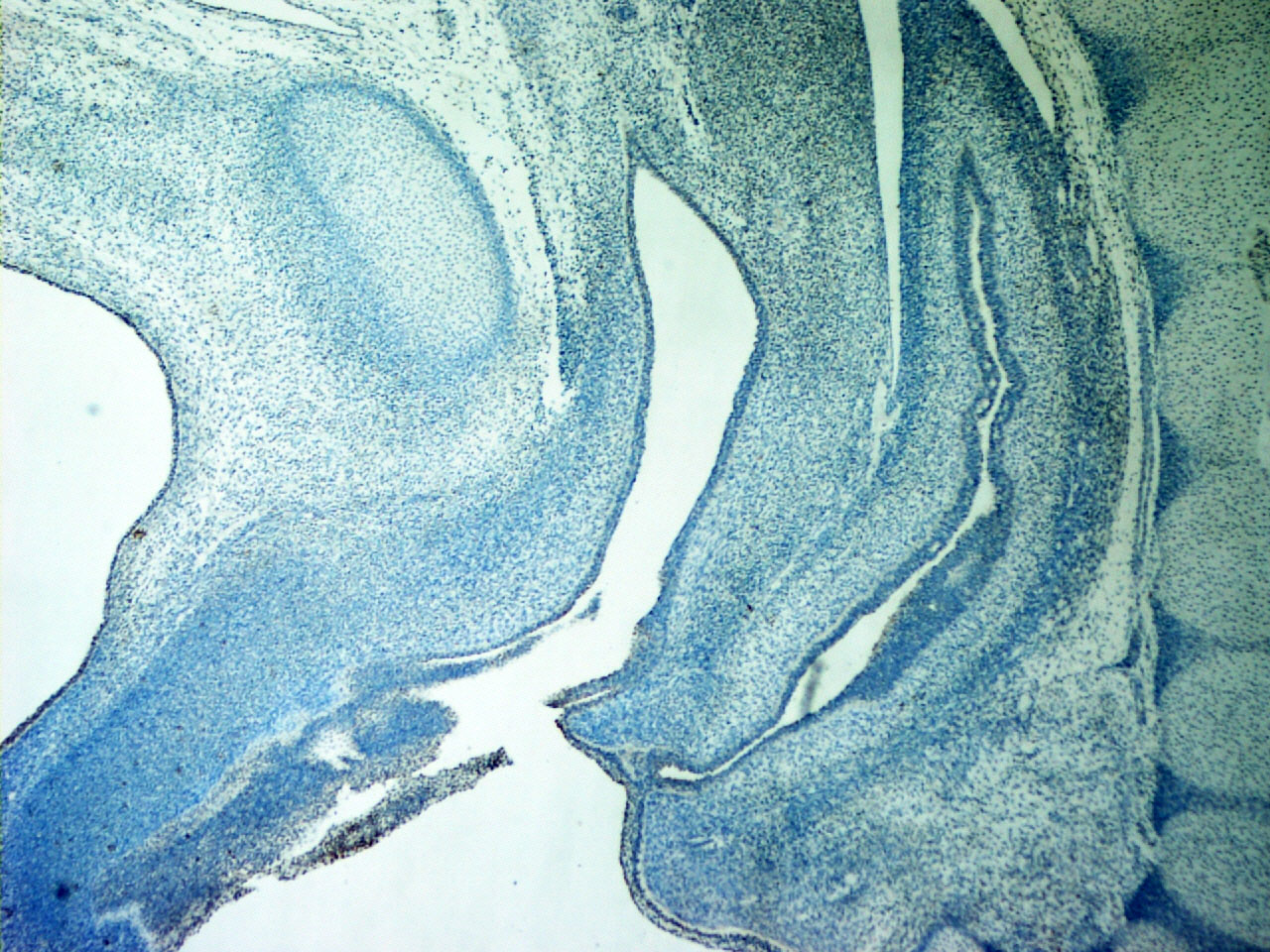

Supplement: Supplemental Information 3 [file peerj-04-1771-s003.zip › 4/c4-50-98-40▒╢╡≈╒√.jpg]

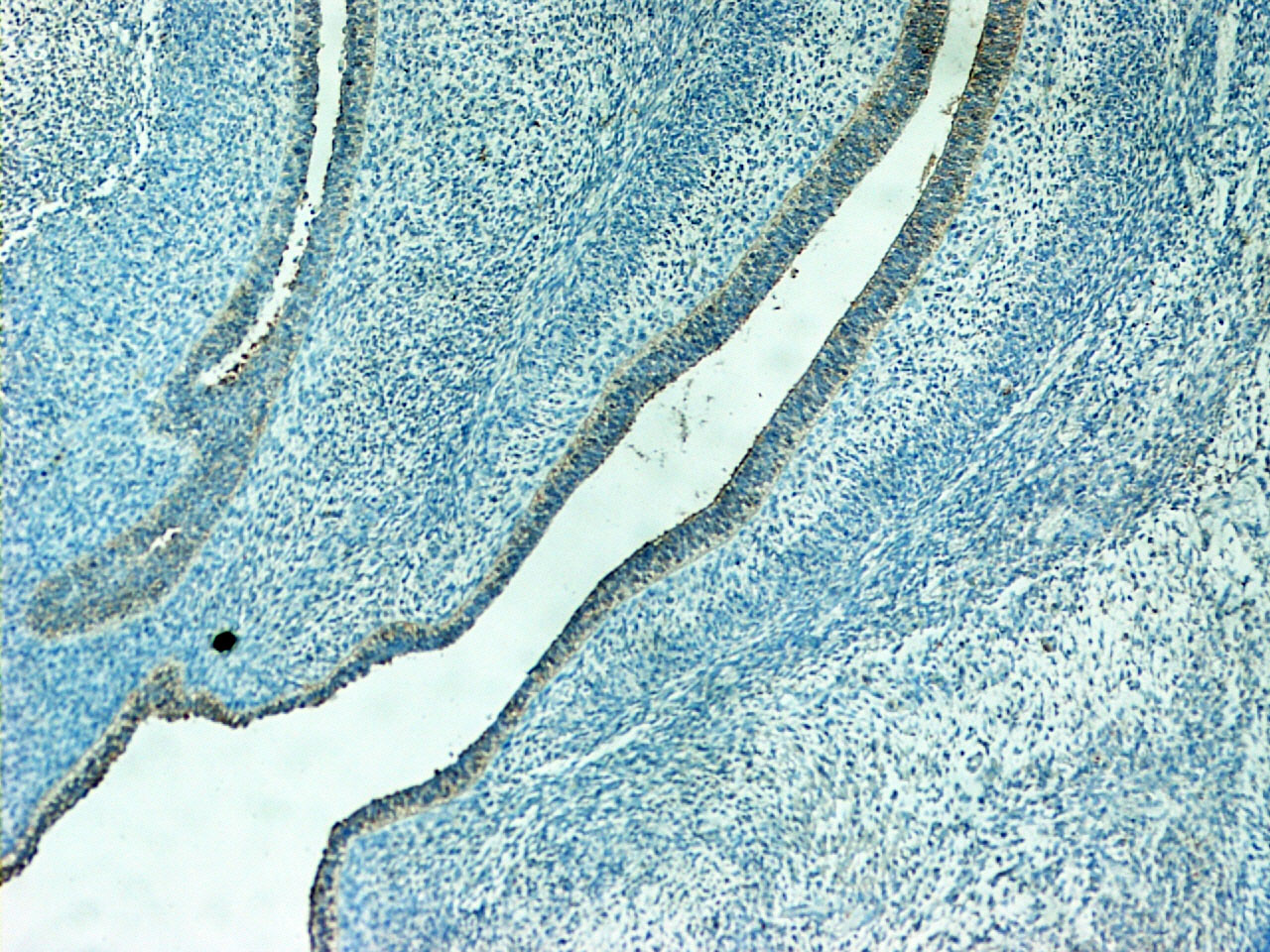

Supplement: Supplemental Information 3 [file peerj-04-1771-s003.zip › 4/c4-51-22-100▒╢''╡≈╒√.jpg]

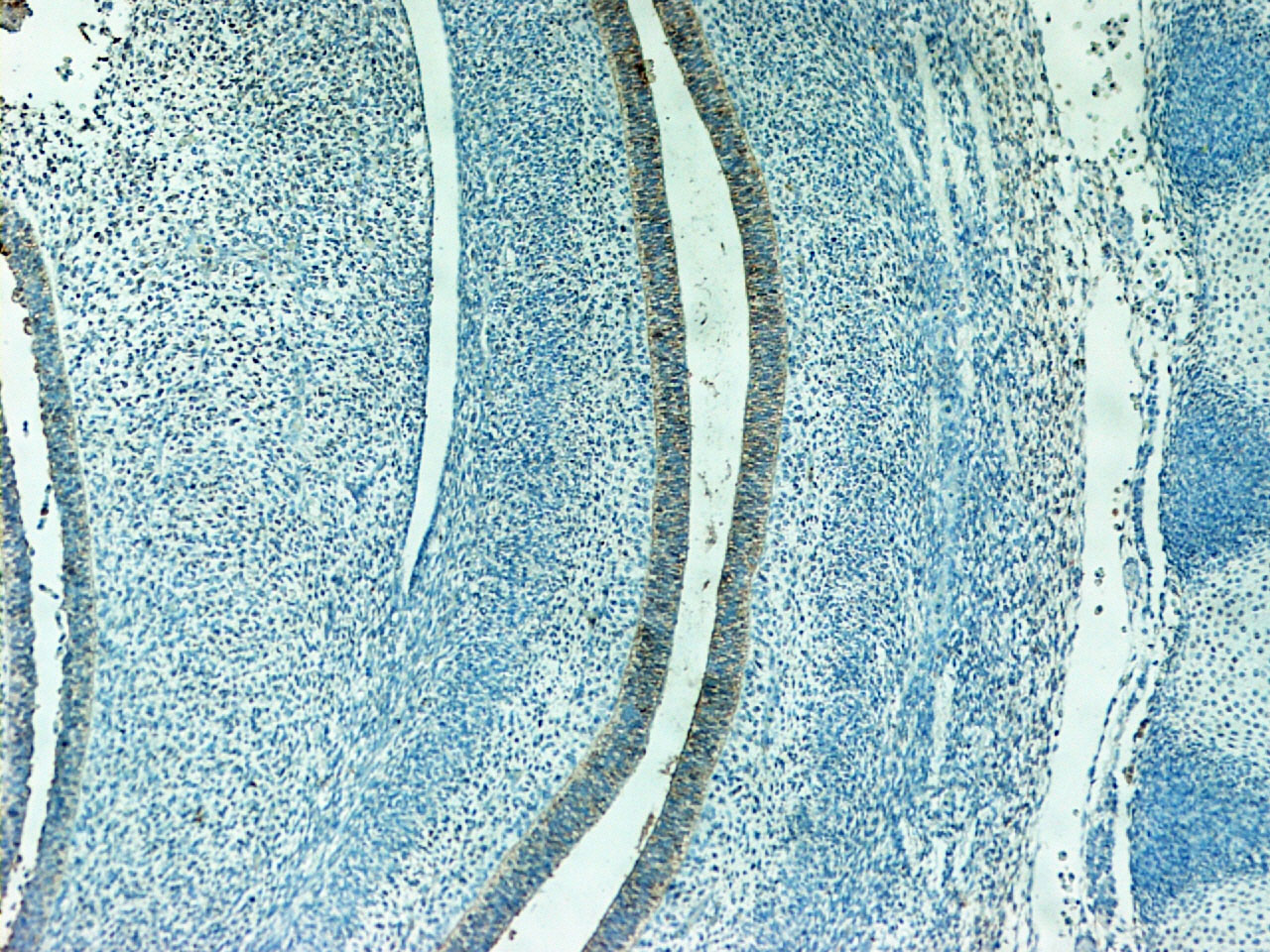

Supplement: Supplemental Information 3 [file peerj-04-1771-s003.zip › 4/c4-51-22-100▒╢'╡≈╒√.jpg]

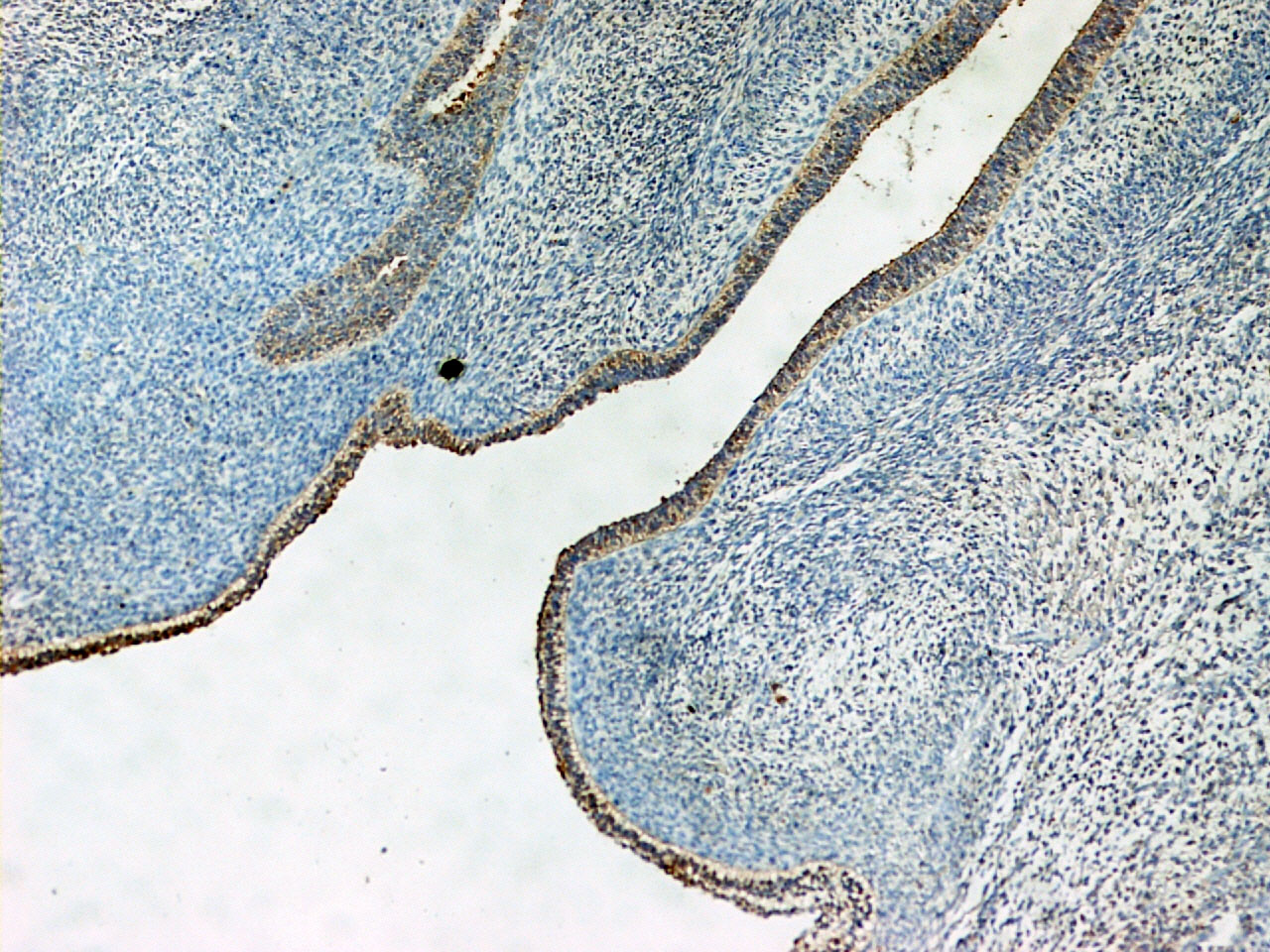

Supplement: Supplemental Information 3 [file peerj-04-1771-s003.zip › 4/c4-51-22-100▒╢╡≈╒√.jpg]

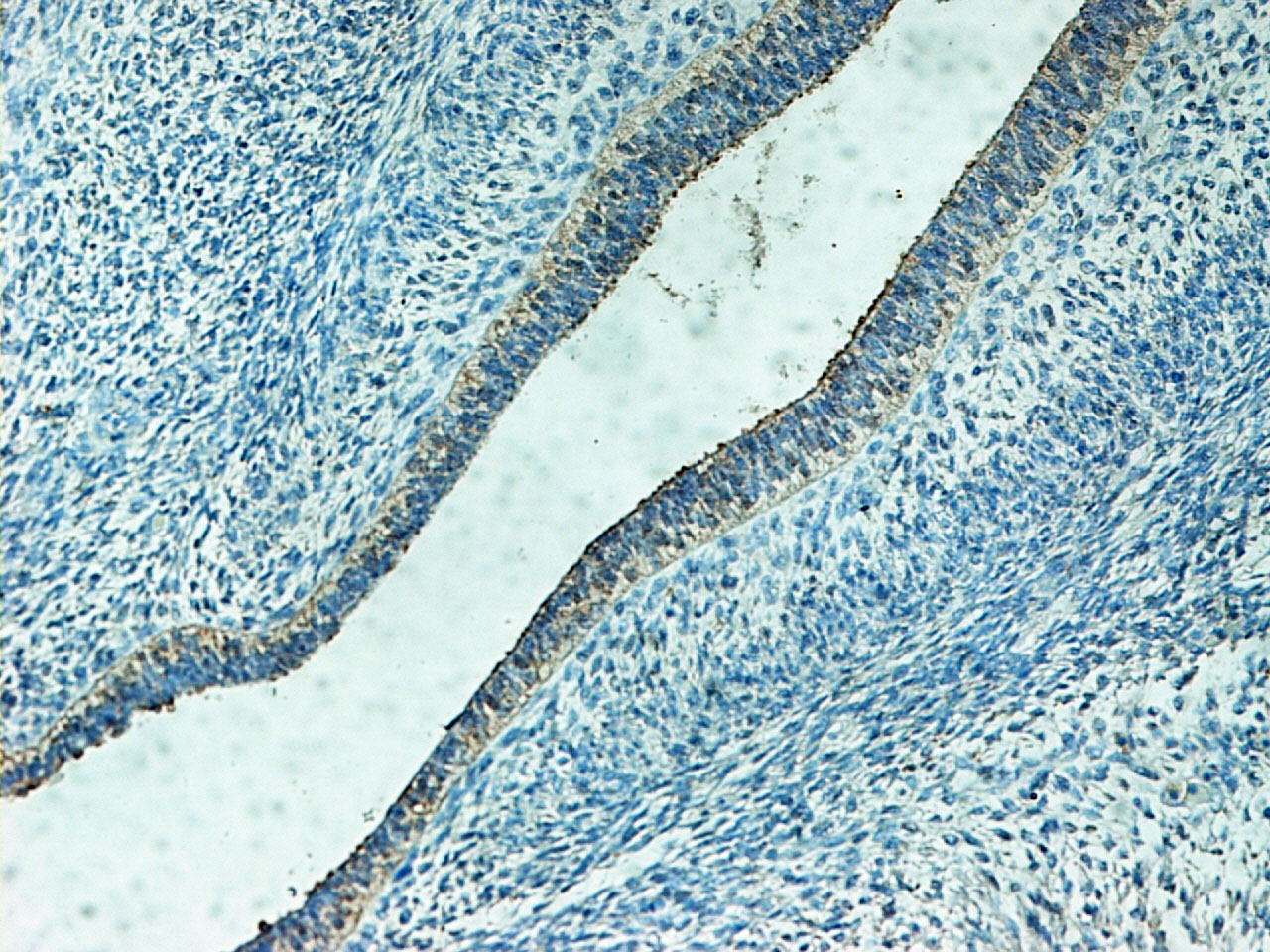

Supplement: Supplemental Information 3 [file peerj-04-1771-s003.zip › 4/c4-51-22-200▒╢''╡≈╒√.jpg]

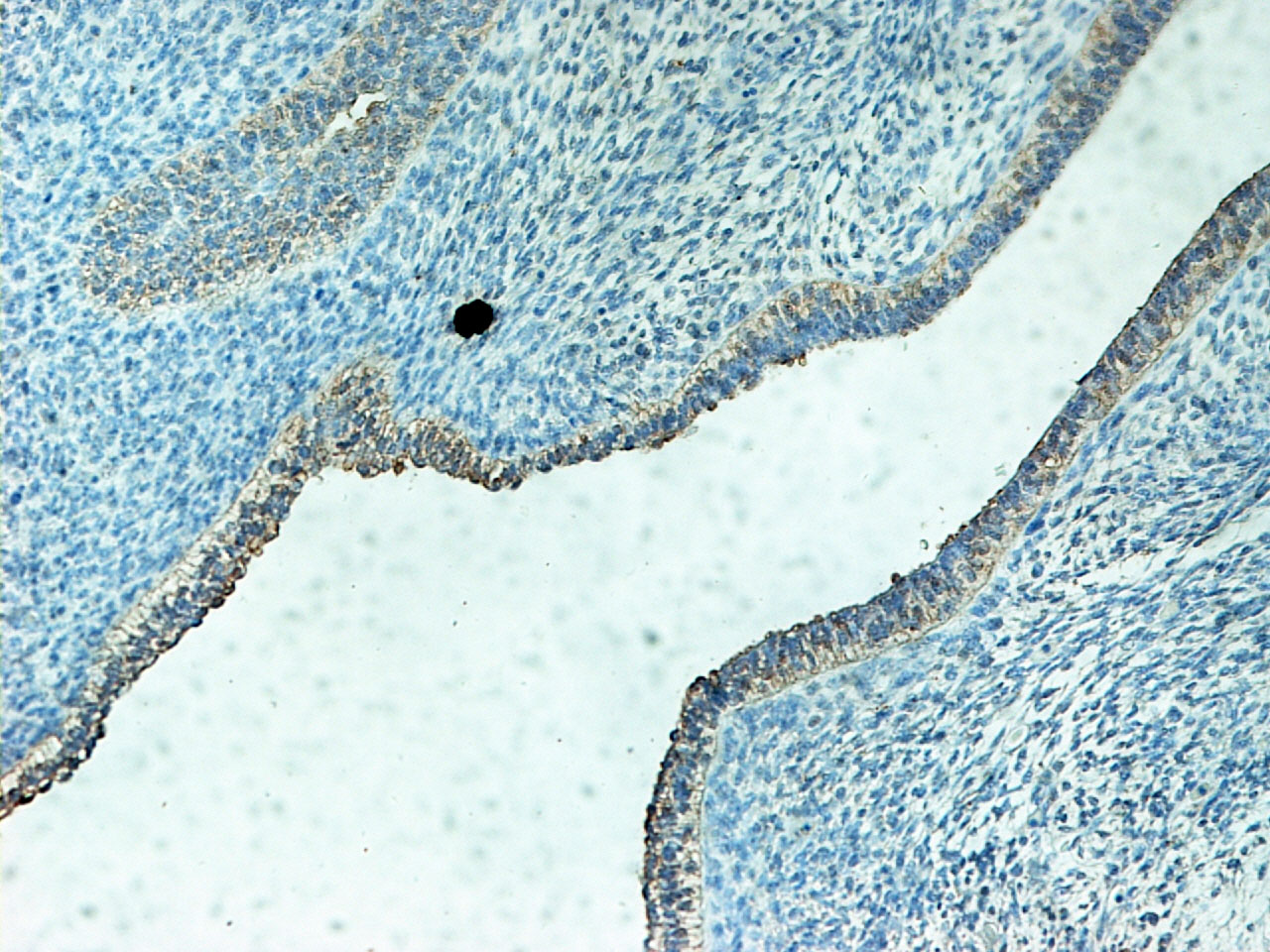

Supplement: Supplemental Information 3 [file peerj-04-1771-s003.zip › 4/c4-51-22-200▒╢╡≈╒√.jpg]

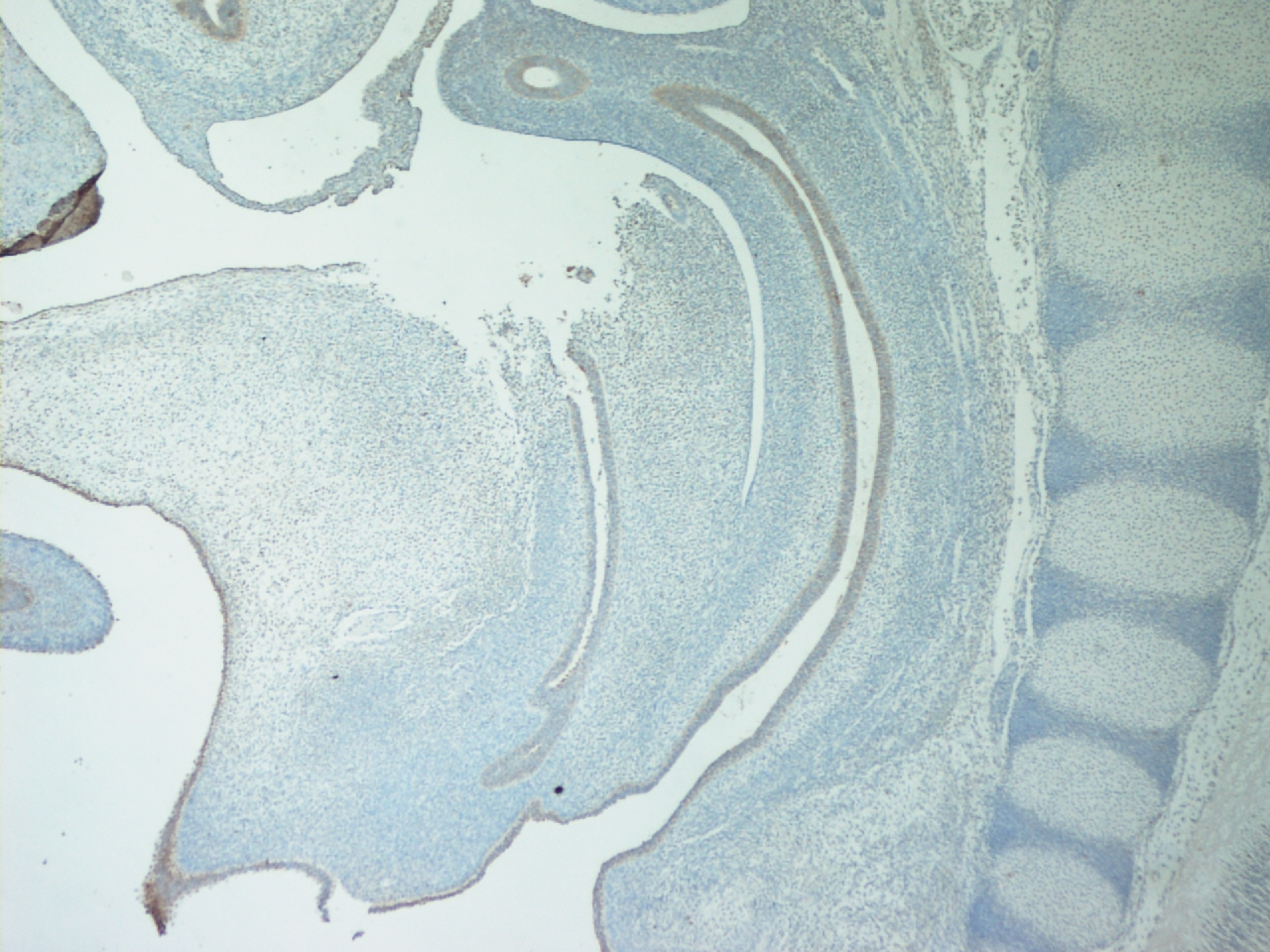

Supplement: Supplemental Information 3 [file peerj-04-1771-s003.zip › 4/c4-51-22-40▒╢.jpg]

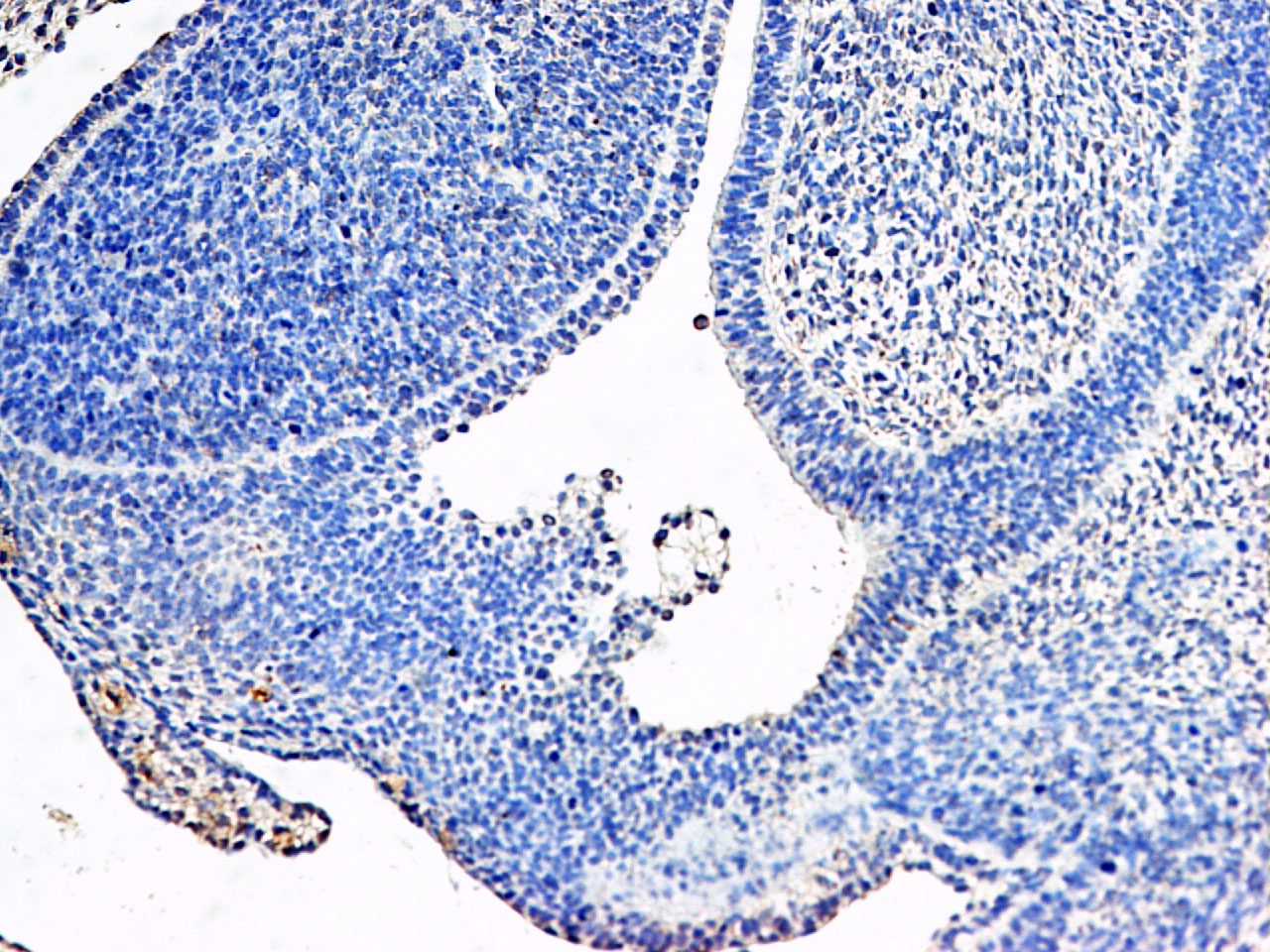

Supplement: Supplemental Information 3 [file peerj-04-1771-s003.zip › 4/C4-7w 200▒╢.jpg]

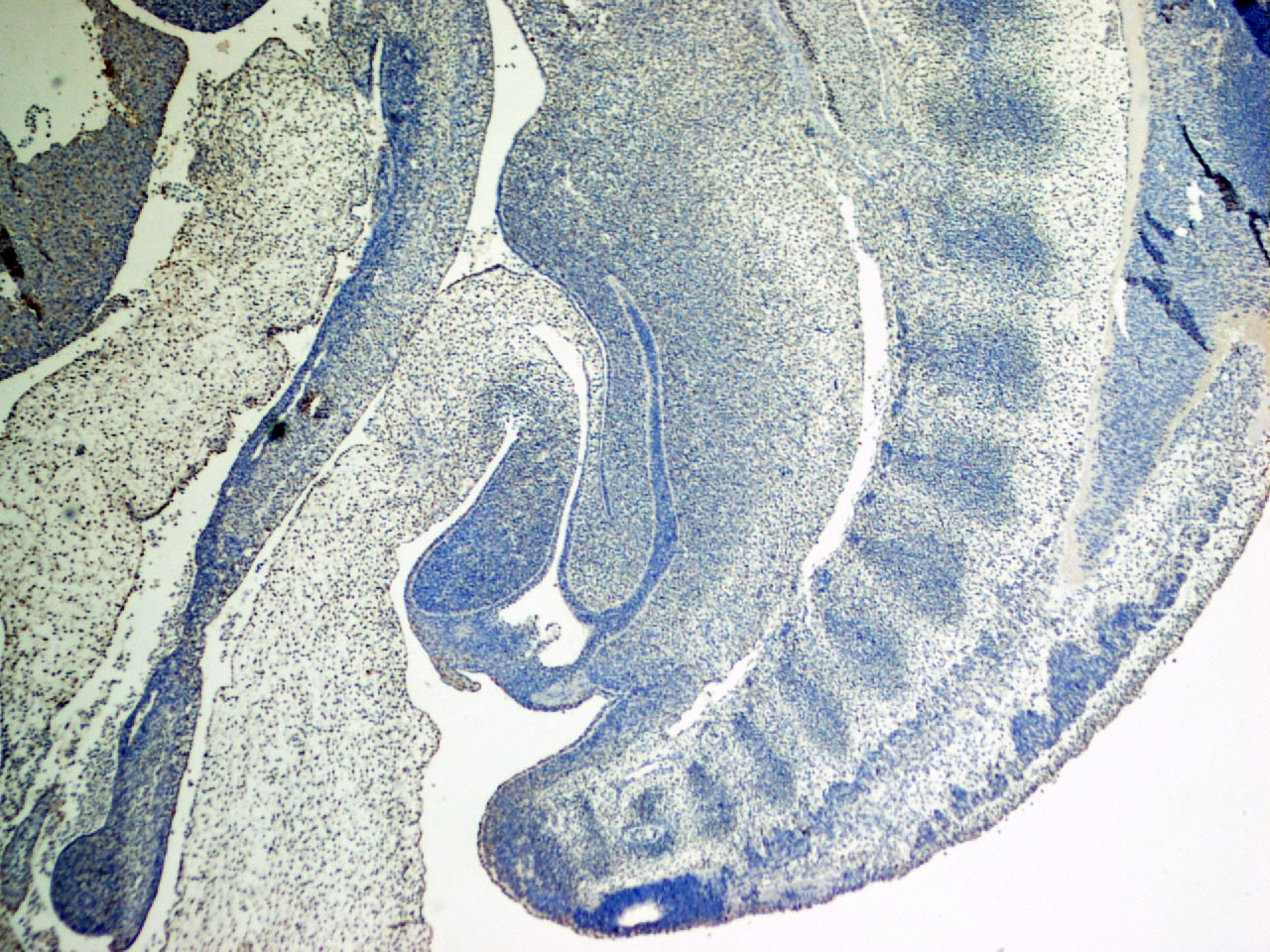

Supplement: Supplemental Information 3 [file peerj-04-1771-s003.zip › 4/C4-7w 40▒╢.jpg]
